# Synthesis and Structural Characterization of Pyridine-2,6-dicarboxamide and Furan-2,5-dicarboxamide Derivatives

**DOI:** 10.3390/molecules27061819

**Published:** 2022-03-10

**Authors:** Anna Puckowska, Magdalena Gawel, Marlena Komorowska, Pawel Drozdzal, Aleksandra Arning, Damian Pawelski, Krzysztof Brzezinski, Marta E. Plonska-Brzezinska

**Affiliations:** 1 Department of Organic Chemistry, Faculty of Pharmacy with the Division of Laboratory Medicine, Medical University of Bialystok, Mickiewicza 2A, 15-222 Bialystok, Poland; damian.pawelski@umb.edu.pl; 2 Department of Structural Biology of Prokaryotic Organisms, Institute of Bioorganic Chemistry, Polish Academy of Sciences, Noskowskiego 12/14, 61-074 Poznan, Poland; mgawel@ibch.poznan.pl (M.G.); mkomorowska@ibch.poznan.pl (M.K.); pdrozdzal@ibch.poznan.pl (P.D.); aleksandra.arning@gmail.com (A.A.); 3 Faculty of Chemistry, A. Mickiewicz University, Uniwersytetu Poznanskiego 8, 60-780 Poznan, Poland

**Keywords:** heterocyclic compounds, dicarboxamide, crystallography, molecular structure, crystal packing, Hirshfeld surface

## Abstract

Derivatives based on pyridine-2-6- and furan-2,5-dicarboxamide scaffolds reveal numerous chemical properties and biological activities. This fact makes them an exciting research topic in supramolecular and coordination chemistry and in discovering new pharmacologically-active compounds. This work aimed to obtain a series of symmetrical pyridine-2-6- and furan-2,5-dicarboxamides through a condensation reaction of the appropriate acyl chlorides and aromatic amides. Successful syntheses were confirmed with NMR spectroscopy. We solved their crystal structures for seven compounds; two pyridine and five furan derivatives. Based on our crystallographic studies, we were able to indicate supramolecular features of the crystals under investigation. Additionally, Hirshfeld surface analysis allowed us to calculate a distribution of intermolecular contacts in the dicarboxamide crystals.

## 1. Introduction

The continuous need for medically and pharmacologically important scaffolds prompts chemists to constantly search for efficient and straightforward pathways to synthesize and modify different heterocycles. This applies mainly to those heterocyclic systems for which some medical or pharmacological activity has already been demonstrated, but their use is still limited due to some structural constraints [1]. The exploitation of multiple binding sites of the pyridine-2,6-dicarboxamide and furan-2,5-dicarboxamide scaffolds makes it possible for the design of various functional materials [2,3], such as sensors [4,5], coordination sites [6,7], structural and functional models of proteins [8,9], derivatives with catalytic and biological activities [10,11,12], derivatives with antibacterial activities [13,14], etc. Pyridine-2,6-dicarboxamide is a chelating ligand for metal cations (Cu, Co, Fe, Ni, Pd), small anions (halides, phosphates and acetates), as well as small not-charged molecules (for example, urea) [1]. Some structurally similar compounds with central *para-* or *meta*-substituted benzene rings can inhibit in vitro proliferation of breast cancer cells [15] or glycogen phosphorylase activity [16]. 2,6-Pyridinecarboxamide derivatives, macrocyclic oligoamides and hydrazides have been screened for their bactericidal and fungicidal potential [17,18]. They exhibit significant antimicrobial activities against Gram-positive, Gram-negative bacteria and fungi comparable to reference antibiotics such as streptomycin, ciprofloxacin and ketoconazole. These properties also give the opportunity to use such heterocycles in catalytic systems in supramolecular and coordination chemistry [19]. In neuroprotection studies employing SH-SY5Y cells, the metal chelator containing the pyridine-2,6-dicarboxamide and quinolone scaffold appeared to exert significant neuroprotection against both Aβ peptide- and H_2_O_2_-induced toxicities [20]. The synthetic derivatives of pyridine-2,6-dicarboxamide exhibit the ability to stabilize telomeric G-quadruplex DNA, which shows the potential of applying these compounds in a senescence-mediated anticancer therapy [21]. 

Although furan derivatives are less prevalent in the literature [22,23,24], several reports also indicate their biological activity. Some of the compounds were reported to improve SIRT1 deacetylase activity, mitochondrial function in murine C2C12 myoblasts, stimulate proliferation in human HaCat cells via NO production and accelerate the skin process repair in an in vivo mouse model [25]. In vitro inhibitory activity against dengue and yellow fever viruses were also reported for the imidazole dicarboxamide derivative [26]. Some first-generation compounds have shown activity against both viruses in the micromolar range.

Crystallography is a powerful method that allows determining the three-dimensional structure of compounds at the atomic or sub-atomic level. It could be applied to small-molecule compounds as well as biological macromolecules. The Cambridge Structural Database (CSD) [27], which serves as the repository for small-molecule organic and metal-organic crystal structures, contains more 1.1 million unique structures. On the other hand, the Protein Data Bank contains almost 190 thousand such macromolecular structures as proteins, nucleic acids and their complexes [28]. Determination of the three-dimensional architecture of a molecule is not the only advantage of crystallography. It also allows a comprehensive study on interactions within molecular crystals that includes, among others, their chemical nature and relation with the packing of molecules within a crystal lattice. These interactions can be easily visualized through Hirshfeld surface analysis [29,30,31,32]. It makes it possible to map close interatomic contacts (longer, equal or shorter than the sum of van der Waals radii) that occur within a crystal lattice on a molecular surface.

Derivatives based on pyridine-2-6- and furan-2,5-dicarboxamide scaffolds are described in material science as model compounds for the study of the effect of intramolecular hydrogen bonding and steric and electronic factors of molecular conformations. Herein, inspired by the numerous applications of these derivatives, symmetrical pyridine-2,6-dicarboxamide derivatives, and for comparison, their analogs containing a furan ring instead of a pyridine core, were synthesized and structurally characterized through X-ray crystallography. Additionally, Hirshfeld surface analysis was used, which allowed us to calculate the distribution of intermolecular contacts in the dicarboxamide crystals. 

## 2. Results and Discussion

### 2.1. Synthesis of Furan-2,5-dicarboxamides and Pyridine-2,6-dicarboxamides

**Scheme 1:**
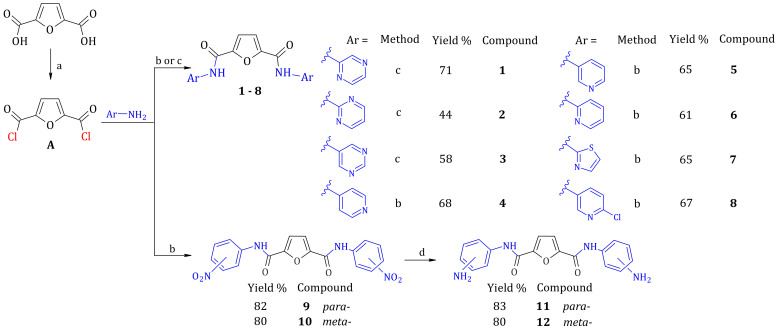
Synthesis of aromatic furan-2,5-dicarboxamides: (a) (CO)_2_Cl_2_ (4–6 eq), DMF (cat.), DCM, RT; (b) 3 or 4-nitroaniline, or 2-, 3-, 4-aminopyridine (2.0 eq), Et_3_N (2.0 eq), DCM/THF (1/1; *v*/*v*), 0 °C ÷ RT, 24 h; (c) 2-aminopyrazine or 2-amino-, or 5-aminopyrimidine (4 eq), DCM/THF (1/1; *v*/*v*), 0 °C ÷ RT, 24 h; (d) N_2_H_4_·H_2_O (7 mL), Pd/C (10% *w*/*w*), MeOH, RT.

**Scheme 2:**
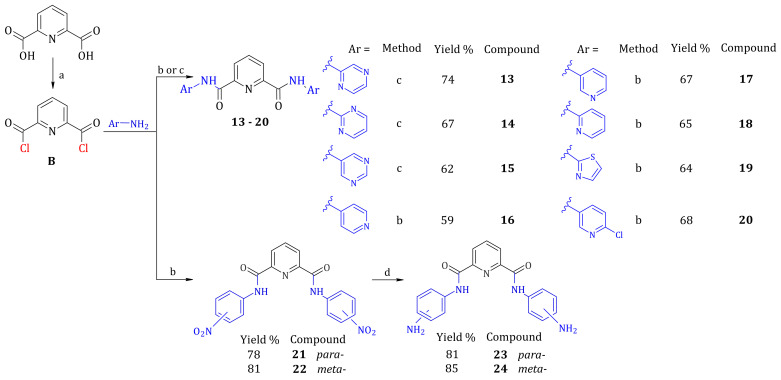
Synthesis of aromatic pyridine-2,6-dicarboxamides: (a) (CO)_2_Cl_2_ (4–6 eq), DMF (cat.), DCM, RT; (b) 3- or 4-nitroaniline, or 2-, 3-, 4-aminopyridine (2.0 eq), Et_3_N (2.0 eq), DCM/THF (1/1; *v*/*v*), 0 °C ÷ RT, 24 h; (c) 2-aminopyrazine or 2-amino-, or 5-aminopyrimidine (4 eq), DCM/THF (1/1; *v*/*v*), 0 °C ÷ RT, 24 h; (d) N_2_H_4_·H_2_O (6 mL), Pd/C (10% *w*/*w*), MeOH, RT.

The synthesis of heterocyclic dicarboxamides (compounds **1**–**8**; **13**–**20**) and *N*-nitrophenyldicarboxamides (compounds **9**, **10**, **21**, **22**) was performed based on a simple condensation of acyl chlorides **A** or **B**, with the appropriate aromatic amines according to Scheme 1 and Scheme 2, starting from furan-2,5-dicarboxylic or pyridine-2,6-dicarboxylic acid, respectively. The pyrimidine and piperazine derivatives were synthesized in the buffer conditions achieved by using an excessive amount of appropriate amines, whereas other compounds were synthesized in the presence of triethylamine. The addition of triethylamine to the reaction mixture effectively prevented the acidification of a reaction environment. Dicarboxamides with nitro groups (compounds **9**, **10**, **21**, **22**) were catalytically reduced to the corresponding diaminodicarboxamides (compounds **11**, **12**, **23**, **24**) with the use of hydrazine monohydrate (100%) and Pd/C (10%). 

### 2.2. Crystallographic Studies of Furan-2,5-dicarboxamides and Pyridine-2,6-dicarboxamides

High-quality monocrystals of the compounds were obtained at room temperature via slow evaporation from their saturated solutions in various solvent systems. Ethyl acetate was used to crystallize compounds **4** and **7**, whereas the compound **7** was crystallized from its methane chloride solution. None of the solvent molecules were present within a crystal lattice of these three compounds. Numerous solvent systems were applied to crystallize other compounds. Finally, crystals were obtained from ethanol/water solution (9.5:0.5, *v*/*v* compound **3**) or acetone/water (9:1, *v*/*v*, compounds **5**, **10** and **16**). It is of note that the presence of water promoted the crystallization of these compounds. 

**Figure 1:**
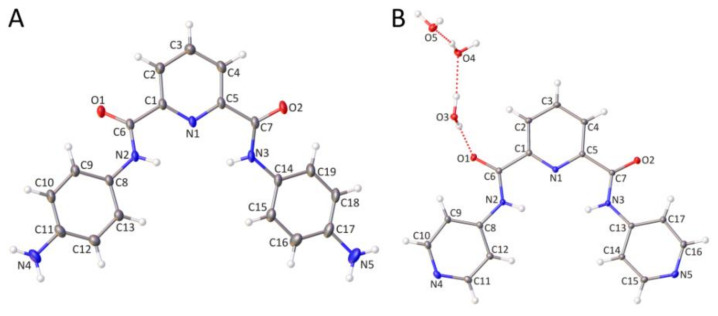
The view of asymmetric unit of compounds (**A**) **23** and (**B**) **16**. Displacement ellipsoids are drawn at the 30% probability level. Possible intermolecular hydrogen bonds are shown as dashed lines.

**Figure 2:**
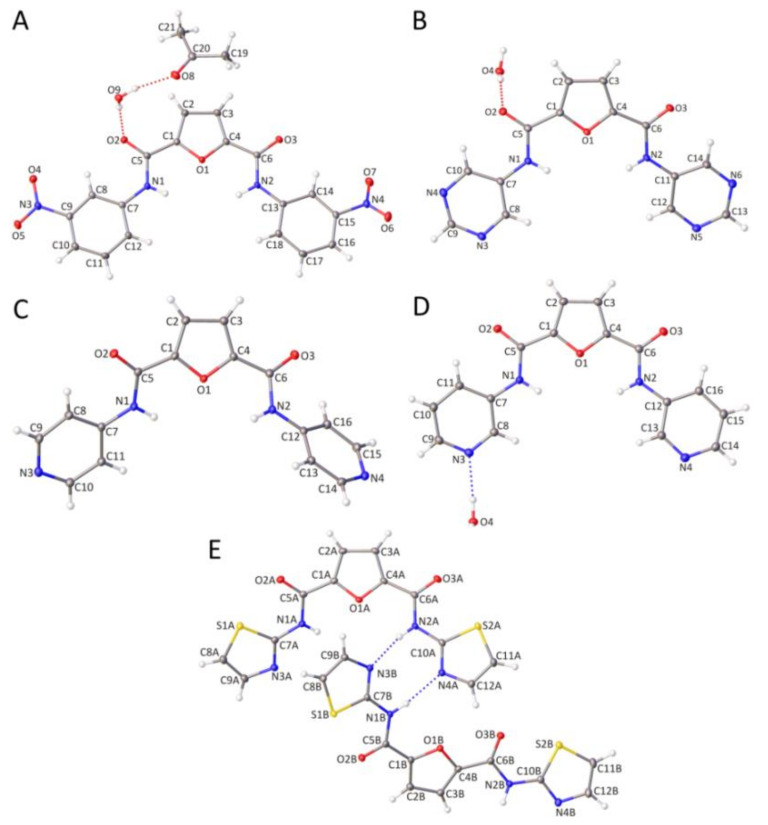
The view of the asymmetric unit (ASU) of compounds (**A**) **10**, (**B**) **3**, (**C**) **4**, (**D**) **5** and (**E**) **7**. For compound **7**, two conformational isomers are present in the ASU. The A-suffix in atom numbering scheme is referred to the *cis*-isomer, whereas the B-suffix to the *trans*-isomer. Displacement ellipsoids are drawn at the 30% probability level. Possible intermolecular hydrogen bonds are shown as dashed lines.

Not surprisingly, these crystals were obtained as hydrates that contained one (compounds **3** and **5**) or three (compound **16**) water molecules in the asymmetric unit (ASU). Additionally, compound **10** crystallized as a solvate with acetone and water in a ratio of 1:1:1. The compounds crystallized in the monoclinic space groups *P*2_1_/*n* or *P*2_1_/*c* with one dicarboxamide molecule in the ASU. An exception was the crystal of compound **7** that contained two configurational isomers in the ASU. The *cis*-isomer is referred to a molecule where two carbonyl oxygen atoms O2A and O3A are on the same side, whereas *trans*-isomer corresponds to a molecule, where carbonyl oxygen atoms O2B and O3B are located on the opposing side. The corresponding crystal structures of pyridine-2,6- and furan-2,5-dicarboxamides are shown in Figure 1 and Figure 2, respectively.

#### 2.2.1. Molecular Structure of Furan-2,5-dicarboxamides and Pyridine-2,6-dicarboxamides

The detailed comparison of the molecular geometry of the analyzed compounds indicates conformational differences between dicarboxamides. This observation is mainly related to deviations from the planarity of carboxamide groups attached to the pyridine or furan rings. Deviations are defined by dihedral angles N2-C6-C1-N1 and N3-C7-C5-N1 for pyridine derivatives or N1-C5-C1-O1 and N2-C6-C4-O1 for furan derivatives, summarized in Table 1. Based on these geometrical parameters, three types of conformation can be observed: planar, semi-skew and skew. For planar conformers (compounds **2**, **3**, and **5**), the deviations from the planarity are relatively small and are in the range from −6.3(2)° to 0.1(4)°. Semi-skew configuration (compound **16**) is characterized by one tiny (around 0°) and one significant planar deviation of carboxamide groups. In contrast, deviations from the planarity for two carboxamide groups are substantial in the skew configuration (compounds **4** and **23**). An interesting situation is observed in the crystal lattice of compound **7**, which contains two dicarboxamide isomers, *cis* (as for other compounds) and *trans* in the asymmetric unit. Herein, the *cis* isomer adopts the semi-skew conformation, whereas the *trans* isomer is the skew conformer.

The planarity’s deviation prevents steric clashes between two amide hydrogen atoms (H2 ∙∙∙ H3 pair for pyridine and H1 ∙∙∙ H2 pair for furan derivatives). Indeed, no too close contacts are observed for these freely refined hydrogen atoms (Table 1). The distance between these two hydrogen atoms range between 2.91(3) and 3.85(2) Å. However, there is no correlation between the conformation of dicarboxamides and the distance between the pair of amide hydrogen atoms. It is of note that slightly closer distances between amide hydrogen atoms are observed in hydrates, where the H–H pair participates in two intermolecular hydrogen bonds with one water molecule. Consequently, the geometry of the H–H interaction might be affected in the structures of hydrates.

#### 2.2.2. Supramolecular Features and Hirshfeld Surface Analysis

A detailed study on intermolecular interactions that occur within crystal lattices of the compounds under investigation was performed. This analysis was supplemented with Hirshfeld surface analysis. Based on that, it was possible to map intermolecular contacts with distances shorter (red color), equal (white color) or more extended (blue color) than the sum of corresponding van der Waals radii. Additionally, two-dimensional (2D) fingerprint plots were generated to evaluate the contribution of close contacts of different natures to the total Hirshfeld surface. 

**Figure 3:**
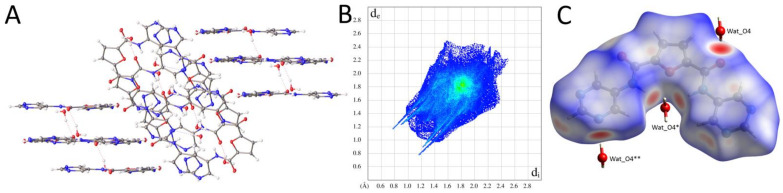
Supramolecular analysis of the crystal of compound **3**: (**A**) crystal packing with hydrogen bond network shown as dashed lines, (**B**) two-dimensional fingerprint plot for all components of the unit cell, and (**C**) Hirshfeld surface mapped over *d_norm_* surrounding the molecules **3** and three symmetry-related water molecules Wat_O4 involved in a formation of hydrogen bond network; water molecules from adjacent assymetric units are marked with * and **.

In the crystal of compound **3**, molecules are packed into nearly perpendicular packs (Figure 3A). The water molecule Wat_O4 is a donor of two hydrogen bonds formed with amide oxygen (Wat_O4 ∙∙∙ O2, 2.826(1) Å) and heterocyclic nitrogen (Wat_O4 ∙∙∙ N5, 2.911(1) Å) atoms and an acceptor of two hydrogen bonds formed with H1 and H2 amide hydrogen atoms, with donor-acceptor distances of 3.079(1) Å for N1 ∙∙∙ Wat_O4 and 3.146(1) Å for N2 ∙∙∙ Wat_O4, respectively. These water-mediated hydrogen bonds are represented by two pairs of sharp spikes on a 2D fingerprint plot (Figure 3B) and correspond to red regions on the Hirshfeld surface (Figure 3C). Additionally, π-stacking interactions are observed between furan and pyrimidine rings with a plane-to-plane centroid distance of 3.442(1) Å. Moreover, pyrimidine rings are also involved in polar π interactions with an amide carbonyl atom (C5) with the carbon atom to plane centroid distance equal to 3.464(1) Å. 

**Figure 4:**
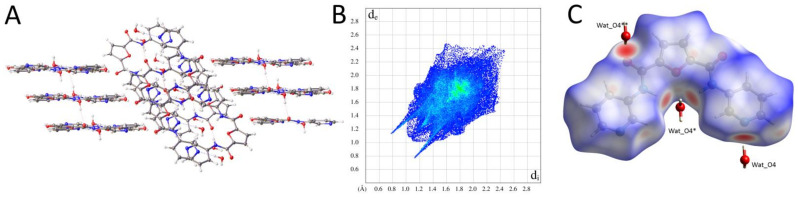
Supramolecular analysis of the crystal of compound **5**: (**A**) crystal packing with hydrogen bond network shown as dashed lines, (**B**) two-dimensional fingerprint plot for all components of the unit cell, and (**C**) Hirshfeld surface mapped over *d_norm_* surrounding the molecule **5** and three symmetry-related water molecules Wat_O4 involved in a formation of hydrogen bond network; water molecules from adjacent assymetric units are marked with * and **.

Crystal packing and the hydrogen bond network facilitated through water molecules is very similar in the crystal of compound **5** to that observed in the crystal of compound **3** (Figure 4A). It includes interactions with amide oxygen (Wat_O4 ∙∙∙ O3, 2.863(1) Å) and heterocyclic nitrogen (Wat_O4 ∙∙∙ N3, 2.854(1) Å) and two amide hydrogen atoms, H1 and H2, with donor-acceptor distances of 3.061(1) Å (N1 ∙∙∙ Wat_O4) and 3.092(1) Å (N2 ∙∙∙ Wat_O4). These contacts are represented by sharp spikes on a 2D fingerprint plot (Figure 4B). Additionally, regions that correspond to these interactions are shown as red regions on the Hirshfeld surface (Figure 4C). Similar to the crystal lattice of compound **3**, π-stacking interactions occur between furan and pyrimidine rings with a plane-to-plane centroid distance of 3.315(1) Å. Additionally, pyrimidine rings are involved in polar π interactions with an amide carbonyl atom (C6) with the carbon atom to plane centroid distance equal to 3.506(1) Å.

**Figure 5:**
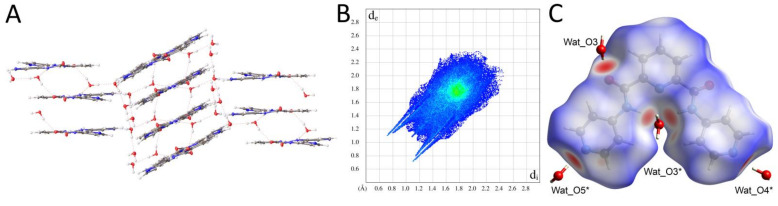
Supramolecular analysis of the crystal of compound **16**: (**A**) crystal packing with hydrogen bond network shown as dashed lines, (**B**) two-dimensional fingerprint plot for all components of the unit cell, and (**C**) Hirshfeld surface mapped over *d_norm_* surrounding the molecule **16** and three water molecules or their symmetry mates involved in a formation of hydrogen bond network; water molecules from adjacent assymetric units are marked with *.

The herring-like motif is observed within the crystal lattice of compound **16** (Figure 5A). The first water molecule (Wat_O3) is a donor of two hydrogen bonds formed with amide oxygen (Wat_O3 ∙∙∙ O2, 2.788(1) Å) and water oxygen (Wat_O3 ∙∙∙ Wat_O4, 2.768(1) Å) atoms and an acceptor of two hydrogen bonds formed with H2 and H3 amide hydrogen atoms with donor-acceptor distances of 2.946(1) Å for N2 ∙∙∙ Wat_O3 and 2.934(1) Å for N3 ∙∙∙ Wat_O3. The Wat_O4 molecule interacts with heterocyclic nitrogen atom N5 (2.802(1) Å). Additionally, the Wat_O4 molecule forms two hydrogen bonds with Wat_O5 (2.790(1) Å) and its symmetrical counterpart Wat_ O5* (2.940(1) Å). The Wat_O5 molecule also serves as a donor in the hydrogen bond formation with the heterocyclic hydrogen atom N4 with a donor-acceptor distance equal to 2.819(2) Å. These hydrogen bonds between water–water and water–**16** molecules correspond to two pairs of sharp spikes on a 2D fingerprint plot (Figure 5B) and are shown as red regions on the Hirshfeld surface (Figure 5C). π-Stacking interactions additionally stabilize the crystal lattice. Herein, a central pyridine ring is sandwiched between two other heterocyclic moieties with a plane-to-plain centroid distance of 3.289(1) Å and 3.527(1) Å. Finally, dipole–dipole interactions between the O2 amide oxygen atom and aromatic hydrogen atoms attached to the pyridine ring are observed.

**Figure 6:**
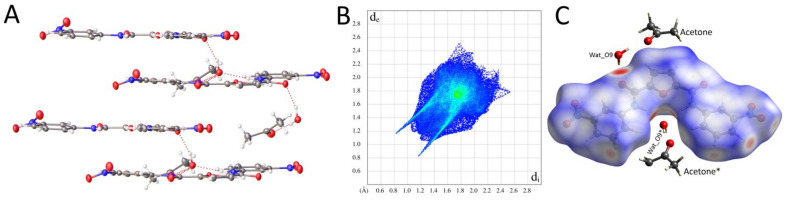
Supramolecular analysis of the crystal of compound **10**: (**A**) crystal packing with hydrogen bond network shown as dashed lines, (**B**) two-dimensional fingerprint plot for all components of the unit cell, and (**C**) Hirshfeld surface mapped over *d_norm_* surrounding the molecule **10** and two symmetry-related water molecules Wat_O9 involved in a formation of hydrogen bond network and two symmetry-related acetone molecules; water molecules from adjacent assymetric units are marked with *.

Compound **10**, together with acetone and water molecules, forms layers within the crystal lattice (Figure 6A). However, molecules of compound **10** are shifted against each other in a manner that prevents any π–π interactions between aromatic rings. Similar to compounds **3** and **4**, a water molecule accepts two amide hydrogen atoms, H1 and H2, with donor-acceptor distances of 2.953(3) Å (N1 ∙∙∙ Wat_O9) or 3.005(3) Å (N2 ∙∙∙ Wat_O9). The water molecule Wat_O9 also serves as a donor of two hydrogen bonds with an amide oxygen atom (Wat_O9 ∙∙∙ O2, 2.795(3) Å) and a carbonyl oxygen atom from acetone molecules (Wat_O9 ∙∙∙ O8, 2.744(3) Å). Like other solvates, the water-mediated hydrogen bonds are shown as sharp spikes on a 2D fingerprint plot (Figure 6B) and are mapped as red regions on the Hirshfeld surface (Figure 6C). The crystal lattice of compound **10** is additionally stabilized by dipole–dipole interactions between nitro groups and aromatic hydrogen atoms attached to furan and phenyl rings, as well as aliphatic hydrogen atoms from acetone molecules. 

**Figure 7:**
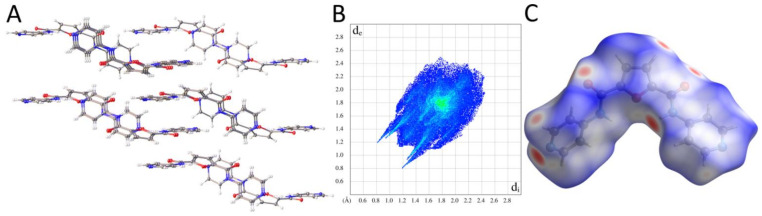
Supramolecular analysis of the crystal of compound **4**: (**A**) crystal packing, (**B**) two-dimensional fingerprint plot, (**C**) Hirshfeld surface mapped over *d_norm_* surrounding the molecule **4**.

An interesting situation is observed within the non-solvate crystal lattice of compound **4**. The molecules adopt the skew conformation and do not stack each other to facilitate π–π interactions (Figure 7A). Herein, each molecule serves as a donor and acceptor of two intermolecular hydrogen bonds between a heterocyclic nitrogen atom (N4 or N3) and an amide nitrogen atom (N1 or N2) with a distance equal to 3.001(1) Å (N1-H1 ∙∙∙ N4) or 3.081(1) Å (N2-H2 ∙∙∙ N3). In addition, the crystal lattice is stabilized by other hydrogen bonds. These include two amide oxygen atoms, O2 and O3, engaged in close contact with aromatic hydrogen atoms attached to pyridine and furan rings. The 2D fingerprint plot (Figure 7B) contains two pairs of sharp spikes that correspond to intermolecular N-H ∙∙∙ N and C-H ∙∙∙ O hydrogen bonds. Additionally, these interactions are mapped on the Hirshfeld surface and are shown as red regions (Figure 7C).

**Figure 8:**
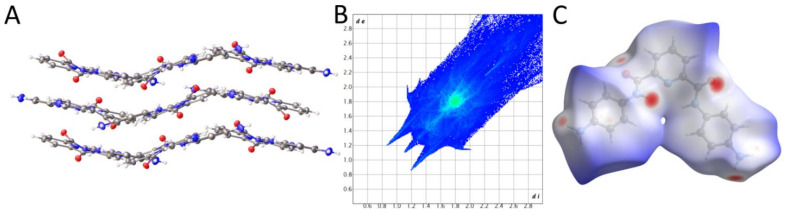
Supramolecular analysis of the crystal of compound **23**: (**A**) crystal packing, (**B**) two-dimensional fingerprint plot, (**C**) Hirshfeld surface mapped over *d_norm_* surrounding the molecule **23**.

The herring-like motif is also observed within the crystal lattice of compound **23** (Figure 8A). Herein, the crystal lattice is stabilized through an intermolecular hydrogen bond network. Each amide oxygen atom is involved in the formation of two hydrogen bonds. The first one corresponds to an interaction with an amide nitrogen atom with a donor-acceptor distance equal to 2.960(2) Å (N3 ∙∙∙ O1) or 3.096(2) Å (N2 ∙∙∙ O2). In comparison, the second hydrogen bond is donated by an amine group attached to a phenyl ring and is characterized by a distance equal to 3.043(3) Å between O1 ∙∙∙ N4 atoms or 3.069(2) Å between O2 ∙∙∙ N5 atoms. These hydrogen bonds are represented by two sharp outer spikes on a 2D fingerprint plot, whereas the central spike corresponds to close contact between aromatic hydrogen atoms H4 and H18 (Figure 8B). Close contacts corresponding to intermolecular hydrogen bonds are shown as red regions on the Hirshfeld surface (Figure 8C). Additionally, an orientation of pyridine and phenyl rings facilitates π-stacking interactions with a plane-to-plane centroid distance of 3.495(1) Å.

**Figure 9:**
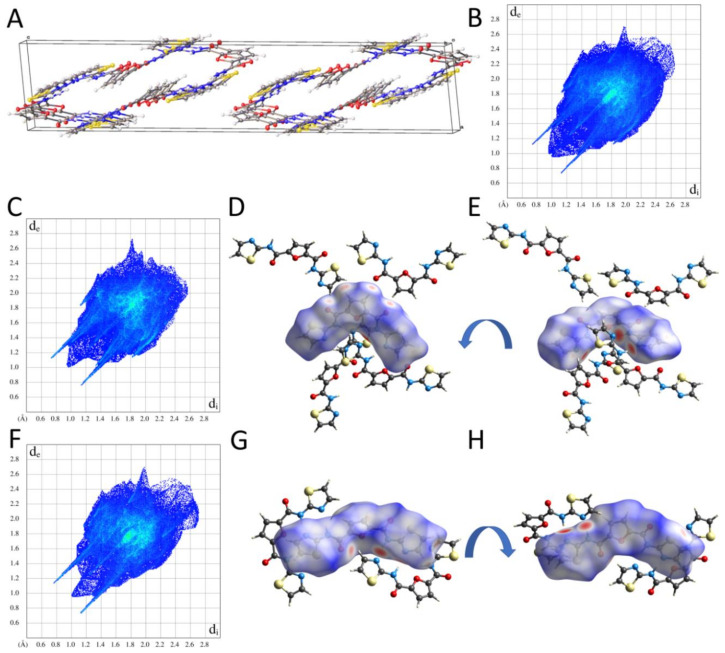
Supramolecular analysis of the crystal of compound **7**: (**A**) crystal packing shown along *b* direction, (**B**) two-dimensional fingerprint plot for all components of the unit cell, (**C**) two-dimensional fingerprint plot for *cis* isomer, (**D**,**E**) Hirshfeld surface mapped over *d_norm_* surrounding the *cis* isomer which forms close contacts with three *trans* and one *cis* isomer molecule/s within the crystal lattice, (**F**) two-dimensional fingerprint plot for *trans* isomer (**G**,**H**) Hirshfeld surface mapped over *d_norm_* surrounding the *trans* isomer which forms close contacts with two *cis* isomer molecules within the crystal lattice.

The crystal of compound **7** is different in comparison to the previously described lattices. A conspicuous difference is the presence of two molecules of dicarboxamide in the asymmetric unit. Closer inspection of the crystal lattice indicates that the asymmetric unit contains two isomers, *cis* and *trans*. Moreover, the isomers adopt two different conformations, namely semi-skew (*cis* isomer) and skew (*trans* isomer). They pack crosswise and form infinite tunnels parallel to the *b* direction (Figure 9A). It is of note that no π-stacking interactions were observed within the crystal lattice of molecule **7**. Dominant intermolecular interactions within the crystal lattice are based on a hydrogen bond network that engages two *cis* and two *trans* isomer molecules. The *cis* isomer (A) donates two hydrogen bonds with two distinct *trans* isomer molecules (B). Within this interaction, each amide group forms a hydrogen bond with a thiazole nitrogen atom with a donor-acceptor distance of 2.873(2) Å (N1A-H1A ∙∙∙ N4B) or 2.834(2) Å (N2A-H ∙∙∙ N3B). Similarly, the *trans* isomer (B) forms two hydrogen bonds with two *cis* isomer molecules (A) via amide groups and heterocyclic nitrogen atoms. The donor-acceptor distances equal 2.896(2) Å for the N1B ∙∙∙ N4A pair and 3.008(2) Å for the N2B ∙∙∙ N3A pair. These close contacts are represented by two sharp spikes on a 2D fingerprint plot, whereas the central highly diffused spike corresponds to numerous close contacts between aromatic hydrogen atoms (Figure 9B). 2D Fingerprint plots, indicating close contacts only for *cis* or *trans* isomers, are shown in Figure 9C or Figure 9F, respectively. Contacts that correspond to intermolecular hydrogen bonds are shown as red regions on the Hirshfeld surface calculated for both isomers separately (Figure 9C–E,G,H). Other close contacts observed within the crystal lattice include interactions between aromatic hydrogen atoms and carbonyl oxygen atoms, as well as thiazole sulfur atoms. It is of note that a 2D fingerprint plot for the *trans* isomer is slightly broader and more diffused than a 2D fingerprint plot for the *cis* isomer. The reason for that is that molecule of the *cis* isomer forms close contacts with four neighboring molecules (three *trans* and one *cis* molecules), whereas the molecule of the *trans* isomer does so with two molecules (two *cis* molecules). As a consequence, the environment of the isomer *cis* is more compact.

#### 2.2.3. Comparison of Crystal Packing and Intermolecular Interactions

The principal interactions observed within crystal lattices of all dicarboxamides under investigation rely on intermolecular hydrogen bonds. It is especially conspicuous for hydrates, where water molecules constitute an essential element of a hydrogen bond network. Not surprisingly, some dicarboxamides could be crystallized only in the presence of water. A comparison of intermolecular interactions between solvate and non-solvate crystals reveals some regularities. In solvate crystals, the hydrogen bond network is based on water molecules involved in close contact with amide oxygen, amide hydrogen, heterocyclic nitrogen atoms and sometimes other solvent molecules (compounds **10** and **16**). On the other hand, hydrogen bonds present in non-solvate crystals are formed between amide nitrogen and heterocyclic nitrogen atoms (compounds **4** and **7**) or amide oxygen and amide nitrogen atoms (compound **23**, where the phenyl ring is present instead of heterocyclic one). 

In most crystals, molecules are packed into nearly perpendicular stacks, facilitating π-stacking interactions. These are observed for the crystals of compounds **3**, **5**, **16** and **23**. On the other hand, no π–π interactions are kept in the crystal of compound **10**, although all molecules are packed into parallel layers formed by compound **2**, water and acetone molecules. Within the crystal of compounds **4** and **7**, molecules are oriented crosswise that prevent any displacement of aromatic rings that could enable π-stacking interactions. Therefore, these are also not present within these two crystal lattices. Additionally, numerous weak interactions are present within all analyzed crystal lattices. These include the dipole–dipole interplay between polar moieties such as nitro groups, amide oxygen atoms, heterocyclic nitrogen and sulfur atoms with aromatic hydrogen atoms.

**Figure 10:**
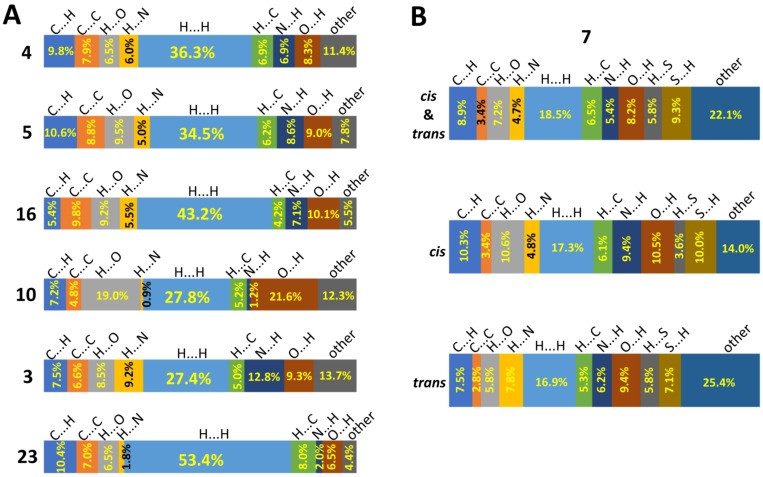
Distribution of intermolecular contacts: (**A**) within crystal lattices of compounds **4**, **5**, **16**, **10**, **3** and **23**; (**B**) within the crystal lattice of compound **7** calculated for the asymmetric unit, which contains one *cis* and one *trans* isomer, as well as for *cis* and *trans* isomers, separately. The distributions are based on Hirshfeld surface analysis; an internal atom of a molecule under investigation is indicated as the first one.

It is of note that the presence or absence of water molecule(s) within the crystal lattice generally does not influence void volumes in analyzed crystals. The percentages of void volumes for solvates are: 7.78% (compound **3**), 7.95% (compound **5**), 8.59% (compound **10**) to 11.96% (compound **16**). Void volumes in two non-solvate crystals are similar: 8.85% (compound **7**) and 9.14% (compound **4**). A similar percentage of void volumes in solvate and two non-solvate crystals results from packing modes within their crystal lattices. In the case of solvates, molecules are packed in crosswise stacks (compounds **3**, **5** and **16**) or form parallel layers (compound **10**). These two kinds of packing might be preferred due to the planar or semi-skew conformation of molecules and, simultaneously, the presence of solvent molecules that fill potential voids and are involved in the intermolecular hydrogen-bond networking. Packaging types in non-solvate crystals are more diverse. Near-perpendicular orientations of molecules are observed in the crystals of compounds **4** and **7**. It ensures a tight packing of molecules and, consequently, facilitates numerous intermolecular interactions of different natures. The comparison of 2D fingerprint plots for solvate and crystals of compounds **4** and **7** reveals they are distributed similarly. However, 2D fingerprint plots for solvate crystals are slightly less diffused. Distances to the nearest atom located intrinsically to the surface (*d_i_*) and exterior to the surface (*d_e_*) reach maximum values of approximately 2.4 Å for solvate crystals and 2.6 Å for tightly-packed non-solvate crystals. In terms of crystal packing, a distinct exception is a non-solvate crystal of molecule **23**, where molecules adopt skew conformation and are packed in layers that form a herring-like motif. Consequently, the percentage of void volume is very high and equal to 26.03%. The Hirshfeld surface mapped for molecule **23** shows that most intermolecular contacts with distances are equal or higher than the sum of van der Waals radii. Additionally, the 2D fingerprint plot is highly diffused. Not surprisingly, a contribution of close contact between hydrogen atoms, equal to 53.5%, is very high compared to other crystals, where a contribution of H ∙∙∙ H interactions ranges from 18.5% to 43.2%. On the other hand, a contribution of close contacts between hydrogen and oxygen or nitrogen or sulfur atoms, equals to 16.8%, is much lower within the crystal lattice of **23**. For the other compounds, these close contacts are an important element of intermolecular interactions, as their contribution ranges from 27.7% to 42.7%. A contribution of non-polar interactions that includes carbon–hydrogen and carbon–carbon close contacts is similar in all analysed crystal lattices and ranges from 17.2% to 25.4%. The distribution of intermolecular contacts in the above-mentioned crystal lattices is shown in Figure 10.

## 3. Materials and Methods

### 3.1. Materials

All substrates for syntheses purchased from Sigma-Aldrich (Merck Life Science, Poznan, Poland) Alfa Aesar (Thermo Fisher GmbH, Kandel, Germany) and TCI Chemicals (TriMen Chemicals S.A., Lodz, Poland) were used as received. All solvents (POCH–Avantor Performance Materials Poland S.A., Gliwice, Poland; Chemopur, Piekary Slaskie, Poland) used for the isolation of the product were distilled and the solvents used for syntheses were purified and dried with the use of standard procedures. Dichloromethane (DCM) was freshly distilled from calcium hydride and tetrahydrofuran (THF) and toluene were distilled over sodium wires. Reaction progress was monitored by thin-layer chromatography (TLC) using silica gel precoated plates (Merck Life Science, Poznan, Poland, 60 mesh, 0.25 mm, aluminum) with UV indicator F-254. TLC chromatograms were visualized with UV light (254 nm) and Ehrlich’s TLC stain (1% solution of *N*,*N*-dimethylaminobenzaldehyde in 96% EtOH). 

Silica gel (Merck, 60 Å, 230–400 mesh) and CHCl_3_/MeOH mixture were prepared with preparative chromatography. The melting point of the synthesized compounds was measured on a Büchi 535 melting point apparatus.

### 3.2. Structural Characterization

#### 3.2.1. NMR Spectra

The NMR spectra were recorded on a Bruker AVANCE II 400 spectrometer operating at 400 MHz for ^1^H NMR and 101 MHz for ^13^C NMR spectra. NMR experiments were performed in deuterodimethyl sulfoxide (DMSO-d_6_), deuterochloroform (CDCl_3_) and deuteromethanol (MeOD) with internal standards at δH = 2.50, 7.26, 3.31 ppm, respectively, for ^1^H NMR and δC = 39.5, 77.2, 49.00 ppm, respectively, for ^13^C NMR. All chemical shifts are reported in ppm, coupling constants J are in Hz and the ^1^H NMR data are reported as s (singlet), d (doublet), t (triplet), dd (doublet of doublet), ddd (doublet of doublet of doublet) or m (multiplet).

#### 3.2.2. X-ray Crystallography and Theoretical Studies

Crystals of compounds **3**, **4**, **5**, **7**, **10**, **16** and **23** were mounted on a loop with paratone. The X-ray diffraction data were measured at 100 K on a SuperNova diffractometer (Rigaku, Tokyo, Japan) with a CCD detector and Cu Kα radiation (compounds **3**, **4**, **5**, **7** and **23**) or Mo Kα radiation (compounds **10** and **16**). All the data were integrated and scaled using the *CrysAlisPro* software package (Rigaku, Tokyo, Japan). The crystal structures were solved using direct methods in the *OLEX2* [33] graphical interface with *SHELXT* [34] and refined with *SHELXL* [35]. All non-hydrogen atoms were refined anisotropically by the full-matrix least-squares method. All hydrogen atoms were initially located in electron-density difference maps. Aromatic hydrogen atoms were constrained to idealized positions with C-H = 0.95 Å and U*iso*(H) = 1.2U*eq*(C). Additionally, due to unresolved disorder in the crystal lattice of compound **23**, amine hydrogen atoms were constrained to idealized positions with N-H = 0.88 Å U*iso*(H) = 1.2U*eq*(N). The water hydrogen atoms (compounds **3**, **5**, **10** and **16**) were freely refined with U*iso*(H) = 1.5U*eq*(O), whereas amide hydrogen atoms were freely refined with U*iso*(H) = 1.2U*eq*(N). The unit cell of molecule **23** contained a solvent accessible void of 220 Å^3^; therefore, the solvent mask procedure implemented in *OLEX2* was applied. Additionally, an analysis of *F*_o_/*F*_c_ data performed with the *TwinRotMat* program from the *PLATON* software package [36] detected a missed twinning for the data of compound **10**. The twin refinement of the crystal structure of compound **10** was performed using *SHELXL*, based on experimental data in the HKLF5 format generated by the *TwinRotMat* program. The *PLATON* software was used to validate the final crystallographic data. The CCDC entries: 2065252 (compound **10**), 2065253 (compound **4**), 2065254 (compound **23**), 2065255 (compound **7**), 2065256 (compound **16**), 2065257 (compound **5**) and 2065258 (compound **3**) contain the supplementary crystallographic data for this paper. These data can be obtained free of charge from the Cambridge Crystallographic Data Centre via www.ccdc.cam.ac.uk/data_request/cif, accessed on 2 January 2022. The final data sets and refinement statistics for all three crystal structures are reported in Supplementary Table S1. 

Hirshfeld surfaces with mapped normalized contact distances *d_norm_* and two-dimensional fingerprint plots showing relationships between distance from the surface to the nearest in the molecule (*d_i_*) and distance from the surface to the nearest atom in neighboring molecule (*d_e_*) were generated using Crystal Explorer 21.5 [37]. 

### 3.3. Synthesis of Compounds

#### 3.3.1. General Procedure for Synthesis of Acid Chlorides (**A**, **B**)

To a suspension of the appropriate dicarboxylic acid in DCM (30 or 50 mL), a catalytic amount of DMF (5–10 drops) was added, followed by the dropwise addition of oxalyl chloride (4–6 eq). The mixture was intensively magnetically stirred at room temperature until a clear solution was formed (3–4 h). Then, solvent and volatile residues were removed on a rotary evaporator under reduced pressure. Additionally, after DCM evaporation, possible residues still remaining in the product were removed by co-azeotropic distillation with anhydrous toluene. The obtained crude acid chloride was then dried in vacuo using a membrane pump (20 °C, 50 mBa, two hours) and used immediately without isolation in the next step. 

#### 3.3.2. General Procedure for Synthesis of Diarylamides

##### General Procedure for Synthesis of Diarylamides **1**–**3** and **13**–**15**

To a 0 °C-cooled solution of the acid chloride in dry DCM (20 mL), the appropriate heterocyclic amine (4.0 eq) was added dropwise as a solution in dry THF (20 mL, 30 min). The resulting mixture was stirred magnetically overnight. The progress of the reaction was monitored by TLC chromatography in the mixture of MeOH/CHCl_3_. After completion of the reaction, the mixture was concentrated by evaporation under reduced pressure, then was dissolved in CHCl_3_ (50 mL) and washed successively with sat. NaHCO_3_ aq (2 × 30 mL), sat. NH_4_Cl aq (2 × 30 mL), sat. NaCl aq (1 × 30 mL). The organic fraction was dried under anhydrous Na_2_SO_4_. The solvent was removed under reduced pressure. If the TLC plate indicated the presence of impurities, the desired product was purified by FCC (flash column chromatography) on silica gel and elution with the mixture of CHCl_3_/MeOH.

##### General Procedure for Synthesis of Diarylamides **4**–**10** and **16**–**22**

To a 0 °C-cooled solution of the acid chloride in dry DCM (30 or 50 mL), a mixture of Et_3_N (2.0 eq) and the appropriate aromatic amine (2.0 eq) was added dropwise as a solution in dry THF (30 mL, 30 min). The resulting mixture was stirred magnetically overnight. The progress of the reaction was monitored by TLC in the mixture of MeOH/CHCl_3_. After completion of the reaction, solvents were removed by evaporation under reduced pressure. The obtained precipitate was suspended in water, filtered off, and washed successively with sat. NaHCO_3_ aq and water. 

#### 3.3.3. General Procedure for Synthesis of Diamines from Dinitro Compounds **11**, **12**, **23** and **24**

To a suspension of the dinitrocompound (0,5 g) in methanol (50 mL), Pd/C (10%, *w*/*w*, 0.05 g) was added, followed by the dropwise addition of hydrazine monohydrate (6–7 mL). The reaction mixture was stirred magnetically at room temperature. The progress of the reaction was monitored by TLC. After the substrate was consumed entirely, the catalyst was removed by filtration through a short, well-tapped pad of Celite^®^545 and washing with methanol. The solvent was evaporated under reduced pressure and the residue was suspended in water. The obtained precipitate was filtered.

##### *N*^2^,*N*^5^-Di(pyrazin-2-yl)furan-2,5-dicarboxamide (Compound **1**)

Compound **1** was prepared from furan-2,5-dicarboxylic acid (0.201 g, 1.28 mmol), oxalyl chloride (0.650 g, 5.12 mmol) and 2-aminopyrazine (0.487 g, 5.12 mmol). The crude product was purified by chromatography to give a white solid (0.282 g, 71%). M.p. 280–281 °C. ^1^H NMR (400 MHz, DMSO-*d_6_*), δ [ppm]: 11.36 (s, 2H), 9.46 (s, 2H), 8.53 (s, 2H), 8.48 (d, *J* = 2.6 Hz, 2H), 7.54 (s, 2H). ^13^C NMR (101 MHz, DMSO-*d_6_*), δ [ppm]: 155.7, 148.2, 147.5, 142.7, 140.6, 137.1, 117.8.

##### *N*^2^,*N*^5^-Di(pyrimidin-2-yl)furan-2,5-dicarboxamide (Compound **2**)

From furan-2,5-dicarboxylic acid (0.2 g, 1.28 mmol), oxalyl chloride (0.437 mL, 0.65 g, 5.12 mmol) and 2-aminopyrimidine (0.487 g, 5.12 mmol), and a white solid of compound **2** (0.176 g, 44.5%) was synthesized after purification by chromatography. M.p. 293–294 °C. ^1^H NMR (400 MHz, DMSO-*d_6_*), δ [ppm]: 10.97 (d, *J* = 9.9 Hz, 2H), 9.37 (d, *J* = 9.9 Hz, 2H), 8.60 (d, *J* = 5.0 Hz, 4H), 7.20 (t, *J* = 4.9 Hz, 2H). ^13^C NMR (101 MHz, DMSO-*d_6_*), δ [ppm]: 163.1, 158.6, 157.6, 117.1.

##### *N*^2^,*N*^5^-Di(pyrimidin-5-yl)furan-2,5-dicarboxamide (Compound **3**)

From furan-2,5-dicarboxylic acid (0.2 g, 1.28 mmol), oxalyl chloride (0.437 mL, 0.65 g, 5.12 mmol) and 5-aminopyrimidine (0.487 g, 5.12 mmol), and a white solid of compound **3** (0.230, 58%) was synthesized after purification by chromatography. M.p. > 300 °C. ^1^H NMR (400 MHz, DMSO-*d_6_*), δ [ppm]: 10.80 (s, 2H), 9.16 (s, 4H), 8.98 (s, 2H), 7.57 (s, 2H). ^13^C NMR (101 MHz, DMSO-*d_6_*), δ [ppm]: 156.0, 153.8, 148. 6, 148.0, 133.6, 116.9.

##### *N*^2^,*N*^5^-Di(pyridin-4-yl)furan-2,5-dicarboxamide (Compound **4**)

From furan-2,5-dicarboxylic acid (0.2 g, 1.28 mmol), oxalyl chloride (0.437 mL, 0.65 g, 5.12 mmol), 4-aminopyridine (0.241 g, 2.56 mmol) and Et_3_N (0.36 mL), and a colorless solid of compound **4** (0.268 g, 68%) was synthesized. M.p. 197–198 °C. ^1^H NMR (400 MHz, DMSO-*d_6_*), δ [ppm]: 10.69 (s, 2H), 8.52 (d, *J* = 4.9 Hz, 4H), 7.77 (d, *J* = 5.0 Hz, 4H), 7.55 (s, 2H). ^13^C NMR (101 MHz, DMSO-*d_6_*), δ [ppm]: 156.4, 150.5, 148.2, 145.4, 116.8, 114.2.

##### *N*^2^,*N*^5^-Di(pyridin-3-yl)furan-2,5-dicarboxamide (Compound **5**)

From furan-2,5-dicarboxylic acid (0.2 g, 1.28 mmol), oxalyl chloride (0.545 mL, 0.813 g, 6.41 mmol), 3-aminopyridine (0.241 g, 2.56 mmol) and Et_3_N (0.36 mL), and a colorless solid of compound **5** (0.256 g, 65%) was synthesized. M.p. 150–151 °C [38]. ^1^H NMR (400 MHz, DMSO-*d_6_*), δ [ppm]: 10.53 (s, 2H), 8.92 (d, *J* = 2.6 Hz, 2H), 8.37 (dd, *J* = 4.8, 1.4 Hz, 2H), 8.16 (ddd, *J* = 8.4, 2.6, 1.5 Hz, 2H), 7.51 (s, 2H), 7.45 (dd, *J* = 8.3, 4.6 Hz, 2H). ^13^C NMR (101 MHz, DMSO-*d_6_*), δ [ppm]: 155.9, 148.2, 145.2, 142.2, 134.7, 127.8, 123.7, 116.4.

##### *N*^2^,*N*^5^-Di(pyridin-2-yl)furan-2,5-dicarboxamide (Compound **6**)

From furan-2,5-dicarboxylic acid (0.2 g, 1.28 mmol), oxalyl chloride (0.437 mL, 0.65 g, 5.12 mmol), 2-aminopyridine (0.241 g, 2.56 mmol) and Et_3_N (0.40 mL), and a colorless solid of compound **6** (0.240 g, 61%) was synthesized. M.p. 144–145 °C. ^1^H NMR (400 MHz, DMSO-*d_6_*), δ [ppm]: 11.20 (s, 2H), 8.44 (m, 2H), 8.21 (d, *J* = 8.3 Hz, 2H), 7.88 (m, 2H), 7.46 (s, 2H), 7.22 (dd, *J* = 7.3, 4.9 Hz, 2H). ^13^C NMR (101 MHz, DMSO-*d_6_*), δ [ppm]: 155.8, 151.3, 148.1, 147.8, 138.5, 120.3, 117.1, 114.5. 

##### *N*^2^,*N*^5^-Di(1,3-thiazol-2-yl)furan-2,5-dicarboxamide (Compound **7**)

From furan-2,5-dicarboxylic acid (0.2 g, 1.28 mmol), oxalyl chloride (0.437 mL, 0.65 g, 5.12 mmol), 1,3-thiazol-2-amine (0.257 g, 2.57 mmol) and Et_3_N (0.36 mL), and a light beige powder of compound **7** (0.256 g, 65%) was synthesized. M.p. 206–207 °C. ^1^H NMR (400 MHz, DMSO-*d_6_*), δ [ppm]: 12.62 (s, 2H), 7.61 (d, *J* = 3.6 Hz, 2H), 7.58 (s, 2H), 7.35 (d, *J* = 3.5 Hz, 2H). ^13^C NMR (101 MHz, DMSO-*d_6_*), δ [ppm]: 157.6, 155.1, 146.9, 137.9, 117.8, 114.6.

##### *N*^2^,*N*^5^-Bis(5-chloropyridin-2-yl)furan-2,5-dicarboxamide (Compound **8**)

From furan-2,5-dicarboxylic acid (0.2 g, 1.28 mmol), oxalyl chloride (0.437 mL, 0.65 g, 5.12 mmol), 5-chloropyridin-2-amine (0.33 g, 2.56 mmol) and Et_3_N (0.36 mL), and a colorless powder of compound **8** (0.325 g, 67%) was synthesized. M.p. 186–187 °C. ^1^H NMR (400 MHz, DMSO-*d_6_*), δ [ppm]: 11.33 (s, 2H), 8.50 (d, *J* = 2.6 Hz, 2H), 8.24 (d, *J* = 8.8 Hz, 2H), 8.01 (dd, *J* = 8.9, 2.7 Hz, 2H), 7.48 (s, 2H). ^13^C NMR (101 MHz, DMSO-*d_6_*), δ [ppm]: 155.8, 150.1, 147.7, 146.5, 138.2, 126.0, 117.3, 115.6.

##### *N*^2^,*N*^5^-Di(4-nitrophenyl)furan-2,5-dicarboxamide (Compound **9**)

From furan-2,5-dicarboxylic acid (0.5 g, 3.2 mmol), oxalyl chloride (1.64 mL, 2.44 g, 19.2 mmol), 4-nitroaniline (0.88 g, 6.4 mmol) and Et_3_N (0.9 mL), and compound **9** (1.04 g, 82%) was synthesized as a light-yellow solid. M.p. 231–232 °C [39]. ^1^H NMR (400 MHz, DMSO-*d_6_*), δ [ppm]: 10.89 (s, 2H), 8.29 (d, *J* = 9.1Hz, 4H), 8.06 (d, *J* = 9.1 Hz, 4H), 7.56 (s, 2H). ^13^C NMR (101 MHz, DMSO-*d_6_*), δ [ppm]: 155.7, 147.9, 144.5, 142.7, 124.4, 120.0, 117.0.

##### *N*^2^,*N*^5^-Di(3-nitrophenyl)furan-2,5-dicarboxamide (Compound **10**)

From furan-2,5-dicarboxylic acid (0.5 g, 3.2 mmol), oxalyl chloride (1.64 mL, 2.44 g, 19.2 mmol), 3-nitroaniline (0.88 g, 6.4 mmol) and Et_3_N (0.9 mL), and compound **10** (1.015 g, 80%) was synthesized as a light-yellow solid. M.p. 286–287 °C [39]. ^1^H NMR (400 MHz, DMSO-*d_6_*), δ [ppm]: 10.84 (s, 2H), 8.75 (t, J = 2.0 Hz, 2H), 8.23 (d, *J* = 9.1 Hz, 4H), 8.00 (dd, *J* = 8.1 Hz, 1.8 Hz, 2H), 7.68 (t, *J* = 8.2 Hz, 2H), 7.53 (s, 2H). ^13^C NMR (101 MHz, DMSO-*d_6_*), δ [ppm]: 155.9, 148.1, 147.9, 139.2, 130.3, 126.3, 118.6, 116.6, 114.5.

##### *N*^2^,*N*^5^-Di(4-aminophenyl)furan-2,5-dicarboxamide (Compound **11**)

From dinitrodicarboxamide (compound **9**) (0.5 g, 1.26 mmol) and NH_2_NH_2_·H_2_O (7 mL) diaminodicarboxamide (compound **11**) (0.352 g, 83%) was synthesized as a yellow solid. M.p. 146–147 °C. ^1^H NMR (400 MHz, DMSO-*d_6_*), δ [ppm]: 9.93 (s, 2H), 7.34 (d, *J* = 8.6 Hz, 4H), 7.27 (s, 2H), 6.59 (d, *J* = 8.6 Hz, 4H), 5.17 (s, 4H). ^13^C NMR (101 MHz, DMSO-*d_6_*), δ [ppm]: 155.0, 148.3, 145.5, 126.6, 122.7, 115.2, 113.9.

##### *N*^2^,*N*^5^-Di(3-aminophenyl)furan-2,5-dicarboxamide (Compound **12**)

From dinitrodicarboxamide (compound **10**) (0.5 g, 1.26 mmol) and NH_2_NH_2_·H_2_O (7 mL) diaminodicarboxamide (compound **12**) (0.34 g, 80%) was synthesized as a yellow solid. M.p. 235–236 °C [39]. ^1^H NMR (400 MHz, DMSO-*d_6_*), δ [ppm]: 9.97 (s, 2H), 7.34 (s, 2H), 7.03 (m, 4H); 6.85 (m, 2H), 6.36 (m, 2H), 5.17 (s, 4H). ^13^C NMR (101 MHz, DMSO-*d_6_*), δ [ppm]: 155.2, 149.1, 148.2, 138.4, 129.0, 115.7, 110.4, 108.4, 106.2.

##### *N*^2^,*N*^6^-Di(pyrazin-2-yl)pyridine-2,6-dicarboxamide (Compound **13**)

From pyridine-2,6-dicarboxylic acid (0.2 g, 1.2 mmol), oxalyl chloride (0.41 mL, 0.61 g, 4.8 mmol) and 2-aminopyrazine (0.455 g, 4.8 mmol), a white solid of compound **13** (0.286 g, 74%) was synthesized. M.p. 206–207 °C. ^1^H NMR (400 MHz, DMSO-*d_6_*), δ [ppm]: 12.08 (s, 2H), 9.55 (s, 2H), 8.58 (s, 2H), 8.51 (d, *J* = 2.3 Hz, 2H), 8.45 (d, *J* = 7.7 Hz, 2H), 8.33 (m, 1H). ^13^C NMR (101 MHz, DMSO-*d_6_*), δ [ppm]: 162.9, 148.4, 148.3, 142.7, 140.6, 140.1, 137.6, 126.4.

##### *N*^2^,*N*^6^-Di(pyrimidin-2-yl)pyridine-2,6-dicarboxamide (Compound **14**)

From pyridine-2,6-dicarboxylic acid (0.2 g, 1.2 mmol), oxalyl chloride (0.41 mL, 0.61 g, 4.8 mmol), and 2-aminopyrimidine (0.455 g, 4.8 mmol), a white solid of compound **14** (0.260 g, 67.6%) was synthesized. M.p. 214–215 °C [40]. ^1^H NMR (400 MHz, DMSO-*d_6_*), δ [ppm]: 11.93 (s, 2H), 8.83 (d, *J* = 4.7 Hz, 4H), 8.40 (d, *J* = 7.6 Hz, 2H), 8.30 (m, 1H), 7.35 (t, *J* = 4.7 Hz, 2H). ^13^C NMR (101 MHz, DMSO-*d_6_*), δ [ppm]: 161.9, 158.6, 157.7, 148.94, 140.0, 126.2, 117.9.

##### *N*^2^,*N*^6^-Di(pyrimidin-5-yl)pyridine-2,6-dicarboxamide (Compound **15**)

From pyridine-2,6-dicarboxylic acid (0.2 g, 1.2 mmol), oxalyl chloride (0.41 mL, 0.61 g, 4.8 mmol), and 5-aminopyrimidine (0.455 g, 4.8 mmol), a white solid of compound **15** (0.238 g, 62%) was synthesized. M.p. 283–284 °C. ^1^H NMR (400 MHz, DMSO-*d_6_*), δ [ppm]: 11.24 (s, 2H), 9.34 (s, 4H), 9.03 (s, 2H), 8.45 (d, J = 7.5 Hz, 2H), 8.37 (dd, J = 8.4, 7.0 Hz, 1H). ^13^C NMR (101 MHz, DMSO-*d_6_*), δ [ppm]: 162.3, 154.0, 148.9, 147.8, 140.5, 133.4, 125.9.

##### *N*^2^,*N*^6^-Di(pyridin-4-yl)pyridine-2,6-dicarboxamide (Compound **16**)

From pyridine-2,6-dicarboxylic acid (0.2 g, 1.2 mmol), oxalyl chloride (0.437 mL, 0.65 g, 5.12 mmol), 4-aminopyridine (0.225 g, 2.38 mmol), and Et_3_N (0.34 mL), a colorless powder of compound **16** (0.225 g, 59%) was synthesized. M.p. > 300 °C [41]. ^1^H NMR (400 MHz, DMSO-*d_6_*), δ [ppm]: 11.26 (s, 2H), 8.59 (d, *J* = 6.3 Hz, 4H), 8.45 (d, *J* = 8.1 Hz, 2H), 8.35 (dd, *J* = 8.5, 6.9 Hz, 1H), 7.97 (d, *J* = 6.3 Hz, 4H). ^13^C NMR (101 MHz, DMSO-*d_6_*), δ [ppm]: 162.6, 150.5, 148.2, 144.9, 140.3, 126.1, 114.4.

##### *N*^2^,*N*^6^-Di(pyridin-3-yl)pyridine-2,6-dicarboxamide (Compound **17**)

From pyridine-2,6-dicarboxylic acid (0.2 g, 1.2 mmol), oxalyl chloride (0.437 mL, 0.65 g, 5.12 mmol), 3-aminopyridine (0.225 g, 2.38 mmol), and Et_3_N (0.34 mL), a colorless powder of compound **17** (0.255 g, 67%) was synthesized. M.p. 238–239 °C [42]. ^1^H NMR (400 MHz, DMSO-*d_6_*), δ [ppm]: 11.16 (s, 2H), 9.11 (s, 2H), 8.43 (m, 4H), 8.33 (m, 3H), 7.50 (dd, *J* = 8.1, 4.6 Hz, 2H). ^13^C NMR (101 MHz, DMSO-*d_6_*), δ [ppm]: 162.1, 148.4, 145.2, 142.5, 140.2, 134.8, 128.2, 125.6, 123.8. 

##### *N*^2^,*N*^6^-Di(pyridin-2-yl)pyridine-2,6-dicarboxamide (Compound **18**)

From pyridine-2,6-dicarboxylic acid (0.2 g, 1.2 mmol), oxalyl chloride (0.437 mL, 0.65 g, 5.12 mmol), 2-aminopyridine (0.225 g, 2.38 mmol), and Et_3_N (0.34 mL), a colorless powder of compound **18** (0.248 g, 65%) was synthesized. M.p. 216–217 °C [43]. ^1^H NMR (400 MHz, DMSO-*d_6_*), δ [ppm]: 11.83 (s, 2H), 8.49 (m, 2H), 8.40 (d, *J* = 7.6 Hz, 2H), 8.31 (m, 3H), 7.91 (t, *J* = 7.4 Hz, 2H), 7.26 (m, 2H). ^13^C NMR (101 MHz, DMSO-*d_6_*), δ [ppm]: 162.7, 151.6, 148.9, 148.0, 139.8, 138.4, 125.9, 120.3, 114.9.

##### *N*^2^,*N*^6^-Di(1,3-thiazol-2-yl)pyridine-2,6-dicarboxamide (Compound **19**)

From pyridine-2,6-dicarboxylic acid (0.2 g, 1.2 mmol), oxalyl chloride (0.437 mL, 0.65 g, 5.12 mmol), 1,3-thiazol-2-amine (0.24 g, 2.4 mmol), and Et_3_N (0.34 mL), a light beige powder of compound **19** (0.254 g, 64%) was synthesized. M.p. 282–283 °C. ^1^H NMR (400 MHz, DMSO-*d_6_*), δ [ppm]: 13.39 (s, 2H), 8.46 (m, 2H), 8.36 (dd, *J* = 8.5, 6.9 Hz, 1H), 7.67 (d, *J* = 3.5 Hz, 2H), 7.41 (d, *J* = 3.5 Hz, 2H). ^13^C NMR (101 MHz, DMSO-*d_6_*), δ [ppm]: 162.1, 158.1, 147.6, 140.4, 138.1, 126.6, 114.7.

##### *N*^2^,*N*^6^-Bis(5-chloropyridin-2-yl)pyridine-2,6-dicarboxamide (Compound **20**)

From pyridine-2,6-dicarboxylic acid (0.2 g, 1.2 mmol), oxalyl chloride (0.437 mL, 0.65 g, 5.12 mmol), 5-chloropyridin-2-amine (0.24 g, 2.4 mmol), and Et_3_N (0.34 mL), a colorless powder of compound **20** (0.316 g, 68%) was synthesized. M.p. 212–213 °C. ^1^H NMR (400 MHz, DMSO*-d_6_*), δ [ppm]: 12.00 (s, 2H), 8.55 (d, *J* = 2.5 Hz, 2H), 8.40 (d, *J* = 7.5 Hz, 2H), 8.36 (d, *J* = 8.9 Hz, 2H), 8.30 (dd, *J* = 8.4, 7.1 Hz, 1H), 8.04 (dd, *J* = 8.9, 2.6 Hz, 2H). ^13^C NMR (101 MHz, DMSO*-d_6_*), δ [ppm]: 162.9, 150.3, 148.7, 146.5, 140.0, 138.3, 126.2, 116.1.

##### *N*^2^,*N*^6^-Di(4-nitrophenyl)pyridine-2,6-dicarboxamide (Compound **21**)

From pyridine-2,6-dicarboxylic acid (0.5 g, 2.99 mmol), oxalyl chloride (1.53 mL, 2.28 g, 18 mmol), 4-nitroaniline (0.83 g, 5.98 mmol), and Et_3_N (0.85 mL), a light-yellow solid of compound **21** (0.95 g, 78%) was synthesized. M.p. > 300 °C [44]. ^1^H NMR (400 MHz, DMSO-*d_6_*), δ [ppm]: 11.38 (s, 2H), 8.44 (d, *J* = 7.6 Hz, 2H), 8.34 (m, 5H), 8.23 (d, *J* = 9.0 Hz, 4H) ppm. ^13^C NMR (101 MHz, DMSO-*d_6_*), δ [ppm]: 162.2, 148.2, 144.2, 143.0, 140.3, 126.1, 124.8, 120.4.

##### *N*^2^,*N*^6^-Di(3-nitrophenyl)pyridine-2,6-dicarboxamide (Compound **22**)

From pyridine-2,6-dicarboxylic acid (0.5 g, 2.99 mmol), oxalyl chloride (1.53 mL, 2.28 g, 18 mmol), 3-nitroaniline (0.83 g, 5.98 mmol) and Et_3_N (0.85 mL), and compound **22** (0.98 g, 81%) as a light-yellow solid, was synthesized. M.p. > 300 °C [44]. ^1^H NMR (400 MHz, DMSO-*d_6_*), δ [ppm]: 11.35 (s, 2H), 8.97(t, *J* = 2.1 Hz, 2H), 8.44 (d, *J* = 7.6 Hz, 2H), 8.34 (m, 3H); 8.04 (m, 2H), 7.75 (t, *J* = 8.1 Hz, 2H). ^13^C NMR (101 MHz, DMSO-*d_6_*), δ [ppm]: 162.2, 148.3, 148.0, 140.3, 139.3, 130.3 126.9, 125.9, 118.9, 114.9.

##### *N*^2^,*N*^6^-Di(4-aminophenyl)pyridine-2,6-dicarboxamide (Compound **23**)

From dinitrodicarboxamide (compound **21**) (0.5 g, 1.23 mmol) and NH_2_NH_2_·H_2_O (6 mL), diaminodicaroboxamide (compound **23**) (0.346 g, 81%) as a yellow solid was synthesized. M.p. 226–227 °C [39]. ^1^H NMR (400 MHz, DMSO-*d_6_*), δ [ppm]: 10.73 (s, 2H), 8.31 (d, *J* = 7.5 Hz, 2H), 8.23 (dd, *J* = 8.4 Hz, 6.8 Hz, 1H), 7.46 (d, *J* = 8.5 Hz, 4H), 6.61 (d, *J* = 8.5, 4H), 5.03 (s, 4H). ^13^C NMR (101 MHz, DMSO-*d_6_*), δ [ppm]: 161.0, 149.2, 145.8, 139.6, 126.7, 124.5, 123.2, 113.7.

##### *N*^2^,*N*^6^-Di(3-aminophenyl)pyridine-2,6-dicarboxamide (Compound **24**)

From dinitrodicarboxamide (compound **22**) (0.5 g, 1.23 mmol) and NH_2_NH_2_·H_2_O (6 mL), diaminodicarboxamide (compound **24**) (0.362 g, 85%) as a light-yellow solid was synthesized. M.p. 247–248 °C. ^1^H NMR (400 MHz, DMSO-*d_6_*), δ [ppm]: 10.79 (s, 2H), 8.36 (d, *J* = 7.2 Hz, 2H), 8.25 (dd, *J* = 8,5 Hz, 6.8 Hz, 1H), 7.20 (t, *J* = 2,0 Hz, 2H), 7.05 (t, *J* = 7,8 Hz, 2H), 6.98 (m, 2H), 6.39 (ddd, *J* = 1,1 Hz, 2,1 Hz, 7, 8 Hz, 2H), 5.17 (s, 4H). ^13^C NMR (101 MHz, DMSO-*d_6_*), δ [ppm]: 161.4, 149.1, 149.1, 139.8, 138.6, 128.9, 125.1, 110.4, 109.1, 106.8.

## 4. Conclusions

In this work, we successfully synthesized symmetrical pyridine-2-6- and furan-2,5-dicarboxamides and confirmed their structures through NMR spectroscopy. In addition, we determined crystal structures for seven compounds, including **3**, **4**, **5**, **7**, **10**, **16** and **23**. A comparison of their molecular geometry indicated that these compounds could adopt three distinct conformations within a crystal lattice, namely nearly planar, semi-skew or skew. We suggest that more minor or significant deviations from the planarity prevent intramolecular steric clashes. Crystallographic studies allowed us to analyze supramolecular features and intermolecular interactions above crystal lattices. Herein, the main observation is that the principal interactions rely on intermolecular hydrogen bonds present in all crystals under investigation. It is evident, especially for hydrates, where water molecules promoted their crystallization. Furthermore, the orientation and conformation of molecules promote π-stacking interactions within crystal lattices of four compounds (**3**, **5**, **16** and **23**). Additionally, numerous weak interactions, based mainly on dipole–dipole interplay, are present in all analyzed crystal lattices.

## Figures and Tables

**Table 1:** Selected geometry parameters for furan-2,5-dicarboxamides and pyridine-2,6-dicarboxamides.

Pyridine Derivative	N2-C6-C1-N1 [°]	N3-C7-C5-N1 [°]	N2-H2 [Å]	N3-H3 [Å]	H2∙∙∙H3 [Å]	Conformation
**23**	20.4(3)	21.5(3)	0.86(2)	0.84(2)	3.39(3)	skew
**16** *	−1.89(1)	−10.1(1)	0.86(2)	0.90(2)	2.91(3)	semi-skew
**Furan derivative**	**N1-C5-C1-O1 [** **°** **]**	**N2-C6-C4-O1 [** **°** **]**	**N1-H1 [Å]**	**N2-H2 [Å]**	**H1∙∙∙H2 [Å]**	
**7** (*cis*)	−19.9(2)	0.8(2)	0.84(2)	0.83(2)	3.85(2)	semi-skew
**7** (*trans*)	17.7(2)	10.2(1)	0.84(2)	0.86(2)	-	skew
**10** *	−6.3(4)	0.1(4)	0.85(4)	0.94(3)	3.25(4)	planar
**3** *	5.0(2)	0.1(2)	0.87(2)	0.84(2)	3.38(2)	planar
**4**	−18.3(2)	−19.2(2)	0.94(1)	0.89(2)	3.82(2)	skew
**5** *	−0.9(2)	−2.5(2)	0.89(2)	0.89(2)	3.39(2)	planar

* solvates.

## Data Availability

Crystallographic data can be obtained free of charge from the Cambridge Crystallographic Data Centre via www.ccdc.cam.ac.uk/data_request/cif (accessed on 2 January 2022).

## Supplementary Materials

The following supporting information can be downloaded at: https://www.mdpi.com/article/10.3390/molecules27061819/s1, Figures S1–S48: ^1^H NMR and ^13^C NMR spectra of compounds **1**–**24**, Table S1: Crystal data and structure refinement details for compounds: **3**, **4**, **5**, **7**, **10**, **16** and **23**.

## Sample Availability

Samples of the compounds are not available from the authors.
