# Supplementary material for: Synthesis and Structural Characterization of Pyridine-2,6-dicarboxamide and Furan-2,5-dicarboxamide Derivatives
Source: Molecules. 2022 Mar 10;27(6):1819. doi: 10.3390/molecules27061819 (PMC8948770; doi:10.3390/molecules27061819)
Supplement: Supplementary file 1 [file molecules-27-01819-s001.zip › molecules-1608746-supplementary.pdf]

Supplementary Material

Synthesis and Structural Characterization of Pyridine-2,6-dicarboxamide and Furan-2,5-dicarboxamide Derivatives

Anna Puckowska [1,*], Magdalena Gawel [2], Marlena Komorowska [2], Pawel Drozdzal [2], Aleksandra Arning [2,3], Damian Pawelski [1], Krzysztof Brzezinski [2,*] and Marta E. Plonska-Brzezinska [1,*]

[1] Department of Organic Chemistry, Faculty of Pharmacy with the Division of Laboratory Medicine, Medical University of Bialystok, Mickiewicza 2A, 15-222 Bialystok, Poland; damian.pawelski@umb.edu.pl
[2] Department of Structural Biology of Prokaryotic Organisms, Institute of Bioorganic Chemistry, Polish Academy of Sciences, Noskowskiego 12/14, 61-074 Poznan, Poland; mgawel@ibch.poznan.pl (M.G.); mkomorowska@ibch.poznan.pl (M.K.); pdrozdzal@ibch.poznan.pl (P.D.); aleksandra.arning@gmail.com (A.A.)
[3] Faculty of Chemistry, A. Mickiewicz University, Uniwersytetu Poznanskiego 8, 60-780 Poznan, Poland
* Correspondence: anna.puckowska@umb.edu.pl (A.P.); kbrzezinski@ibch.poznan.pl (K.B.); marta.plonska-brzezinska@umb.edu.pl (M.E.P.-B.)

Contents:

Figure S1: ^{1}H NMR spectrum of *N*^{2},*N*^{5}-di(pyrazin-2-yl)furan-2,5-dicarboxamide (**1**).
Figure S2: ^{13}C NMR spectrum of *N*^{2},*N*^{5}-di(pyrazin-2-yl)furan-2,5-dicarboxamide (**1**).
Figure S3: ^{1}H NMR spectrum of *N*^{2},*N*^{5}-di(pyrimidin-2-yl)furan-2,5-dicarboxamide (**2**).
Figure S4: ^{13}C NMR spectrum of *N*^{2},*N*^{5}-di(pyrimidin-2-yl)furan-2,5-dicarboxamide (**2**).
Figure S5: ^{1}H NMR spectrum of *N*^{2},*N*^{5}-di(pyrimidin-5-yl)furan-2,5-dicarboxamide (**3**).
Figure S6: ^{13}C NMR spectrum of *N*^{2},*N*^{5}-di(pyrimidin-5-yl)furan-2,5-dicarboxamide (**3**).
Figure S7: ^{1}H NMR spectrum of *N*^{2},*N*^{5}-di(pyridin-4-yl)furan-2,5-dicarboxamide (**4**).
Figure S8: ^{13}C NMR spectrum of *N*^{2},*N*^{5}-di(pyridin-4-yl)furan-2,5-dicarboxamide (**4**).
Figure S9: ^{1}H NMR spectrum of *N*^{2},*N*^{5}-di(pyridin-3-yl)furan-2,5-dicarboxamide (**5**).
Figure S10: ^{13}C NMR spectrum of *N*^{2},*N*^{5}-di(pyridin-3-yl)furan-2,5-dicarboxamide (**5**).
Figure S11: ^{1}H NMR spectrum of *N*^{2},*N*^{5}-di(pyridin-2-yl)furan-2,5-dicarboxamide (**6**).
Figure S12: ^{13}C NMR spectrum of *N*^{2},*N*^{5}-di(pyridin-2-yl)furan-2,5-dicarboxamide (**6**).
Figure S13: ^{1}H NMR spectrum of *N*^{2},*N*^{5}-di(1,3-thiazol-2-yl)furan-2,5-dicarboxamide (**7**).
Figure S14: ^{13}C NMR spectrum of *N*^{2},*N*^{5}-di(1,3-thiazol-2-yl)furan-2,5-dicarboxamide (**7**).

Figure S15: ^{1}H NMR spectrum of N^2,N^5-bis(5-chloropyridin-2-yl)furan-2,5-dicarboxamide (**8**).
Figure S16: ^{13}C NMR spectrum of N^2,N^5-bis(5-chloropyridin-2-yl)furan-2,5-dicarboxamide (**8**).
Figure S17: ^{1}H NMR spectrum of N^2,N^5-di(4-nitrophenyl)furan-2,5-dicarboxamide (**9**).
Figure S18: ^{13}C NMR spectrum of N^2,N^5-di(4-nitrophenyl)furan-2,5-dicarboxamide (**9**).
Figure S19: ^{1}H NMR spectrum of N^2,N^5-di(3-nitrophenyl)furan-2,5-dicarboxamide (**10**).
Figure S20: ^{13}C NMR spectrum of N^2,N^5-di(3-nitrophenyl)furan-2,5-dicarboxamide (**10**).
Figure S21: ^{1}H NMR spectrum of N^2,N^5-di(4-aminophenyl)furan-2,5-dicarboxamide (**11**).
Figure S22: ^{13}C NMR spectrum of N^2,N^5-di(4-aminophenyl)furan-2,5-dicarboxamide (**11**).
Figure S23: ^{1}H NMR spectrum of N^2,N^5-di(3-aminophenyl)furan-2,5-dicarboxamide (**12**).
Figure S24: ^{13}C NMR spectrum of N^2,N^5-di(3-aminophenyl)furan-2,5-dicarboxamide (**12**).
Figure S25: ^{1}H NMR spectrum of N^2,N^6-di(pyrazin-2-yl)pyridine-2,6-dicarboxamide (**13**).
Figure S26: ^{13}C NMR spectrum of N^2,N^6-di(pyrazin-2-yl)pyridine-2,6-dicarboxamide (**13**).
Figure S27: ^{1}H NMR spectrum of N^2,N^6-di(pyrimidin-2-yl)pyridine-2,6-dicarboxamide (**14**).
Figure S28: ^{13}C NMR spectrum of N^2,N^6-di(pyrimidin-2-yl)pyridine-2,6-dicarboxamide (**14**).
Figure S29: ^{1}H NMR spectrum of N^2,N^6-di(pyrimidin-5-yl)pyridine-2,6-dicarboxamide (**15**).
Figure S30: ^{13}C NMR spectrum of N^2,N^6-di(pyrimidin-5-yl)pyridine-2,6-dicarboxamide (**15**).
Figure S31: ^{1}H NMR spectrum of N^2,N^6-di(pyridin-4-yl)pyridine-2,6-dicarboxamide (**16**).
Figure S32: ^{13}C NMR spectrum of N^2,N^6-di(pyridin-4-yl)pyridine-2,6-dicarboxamide (**16**).
Figure S33: ^{1}H NMR spectrum of N^2,N^6-di(pyridin-3-yl)pyridine-2,6-dicarboxamide (**17**).
Figure S34: ^{13}C NMR spectrum of N^2,N^6-di(pyridin-3-yl)pyridine-2,6-dicarboxamide (**17**).
Figure S35: ^{1}H NMR spectrum of N^2,N^6-di(pyridin-2-yl)pyridine-2,6-dicarboxamide (**18**).
Figure S36: ^{13}C NMR spectrum of N^2,N^6-di(pyridin-2-yl)pyridine-2,6-dicarboxamide (**18**).
Figure S37: ^{1}H NMR spectrum of N^2,N^6-di(1,3-thiazol-2-yl)pyridine-2,6-dicarboxamide (**19**).
Figure S38: ^{13}C NMR spectrum of N^2,N^6-di(1,3-thiazol-2-yl)pyridine-2,6-dicarboxamide (**19**).
Figure S39: ^{13}C NMR spectrum of N^2,N^6-bis(5-chloropyridin-2-yl)pyridine-2,6-dicarboxamide (**20**).
Figure S40: ^{13}C NMR spectrum of N^2,N^6-bis(5-chloropyridin-2-yl)pyridine-2,6-dicarboxamide (**20**).
Figure S41: ^{1}H NMR spectrum of N^2,N^6-di(4-nitrophenyl)pyridine-2,6-dicarboxamide (**21**).
Figure S42: ^{13}C NMR spectrum of N^2,N^6-di(4-nitrophenyl)pyridine-2,6-dicarboxamide (**21**).
Figure S43: ^{1}H NMR spectrum of N^2,N^6-di(3-nitrophenyl)pyridine-2,6-dicarboxamide (**22**).
Figure S44: ^{13}C NMR spectrum of N^2,N^6-di(3-nitrophenyl)pyridine-2,6-dicarboxamide (**22**).
Figure S45: ^{1}H NMR spectrum of N^2,N^6-di(4-aminophenyl)pyridine-2,6-dicarboxamide (**23**).
Figure S46: ^{13}C NMR spectrum of N^2,N^6-di(4-aminophenyl)pyridine-2,6-dicarboxamide (**23**).
Figure S47: ^{1}H NMR spectrum of N^2,N^6-di(3-aminophenyl)pyridine-2,6-dicarboxamide (**24**).
Figure S48: ^{13}C NMR spectrum of N^2,N^6-di(3-aminophenyl)pyridine-2,6-dicarboxamide (**24**).
Table S1. Crystal data and structure refinement details for compounds: **3**, **4**, **5**, **7**, **10**, **16** and **23.**

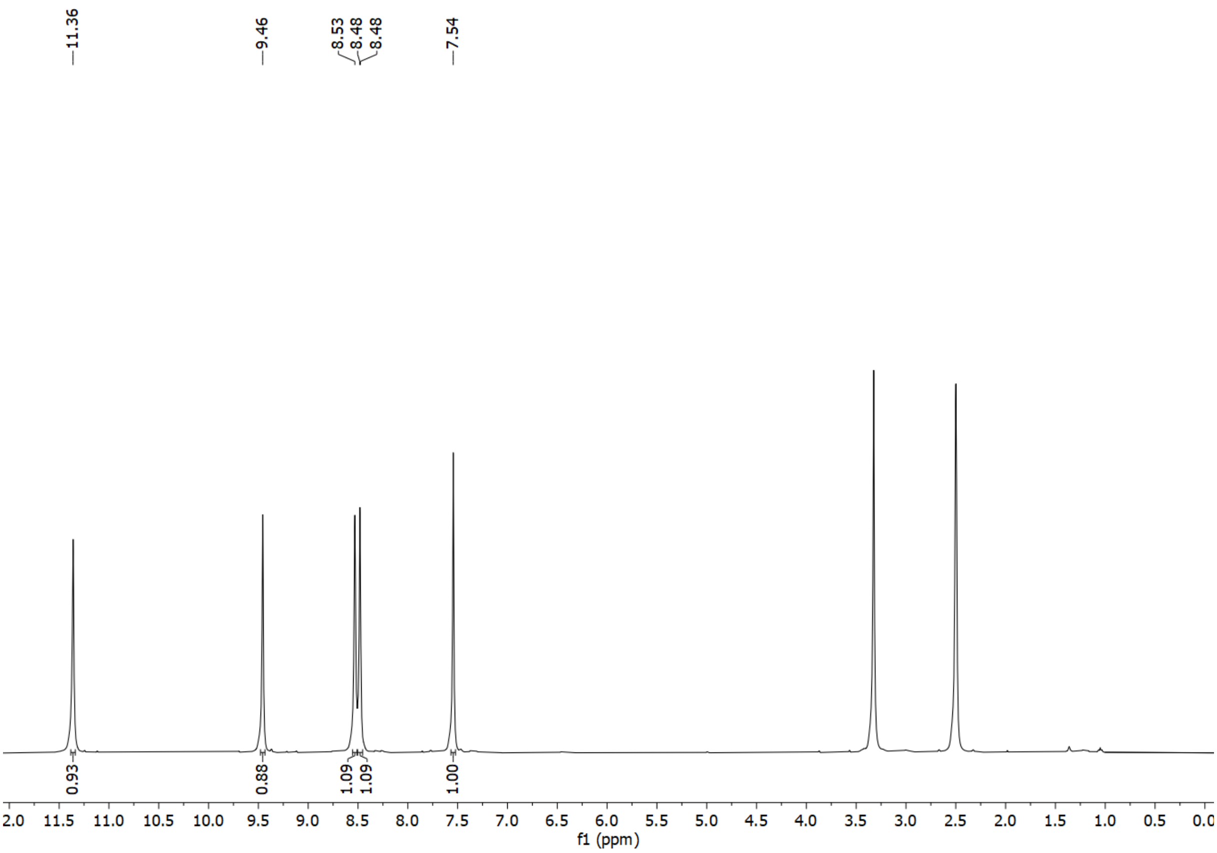

Figure S1. ^{1}H NMR spectrum of *N*^{2},*N*^{5}-di(pyrazin-2-yl)furan-2,5-dicarboxamide (**1**).

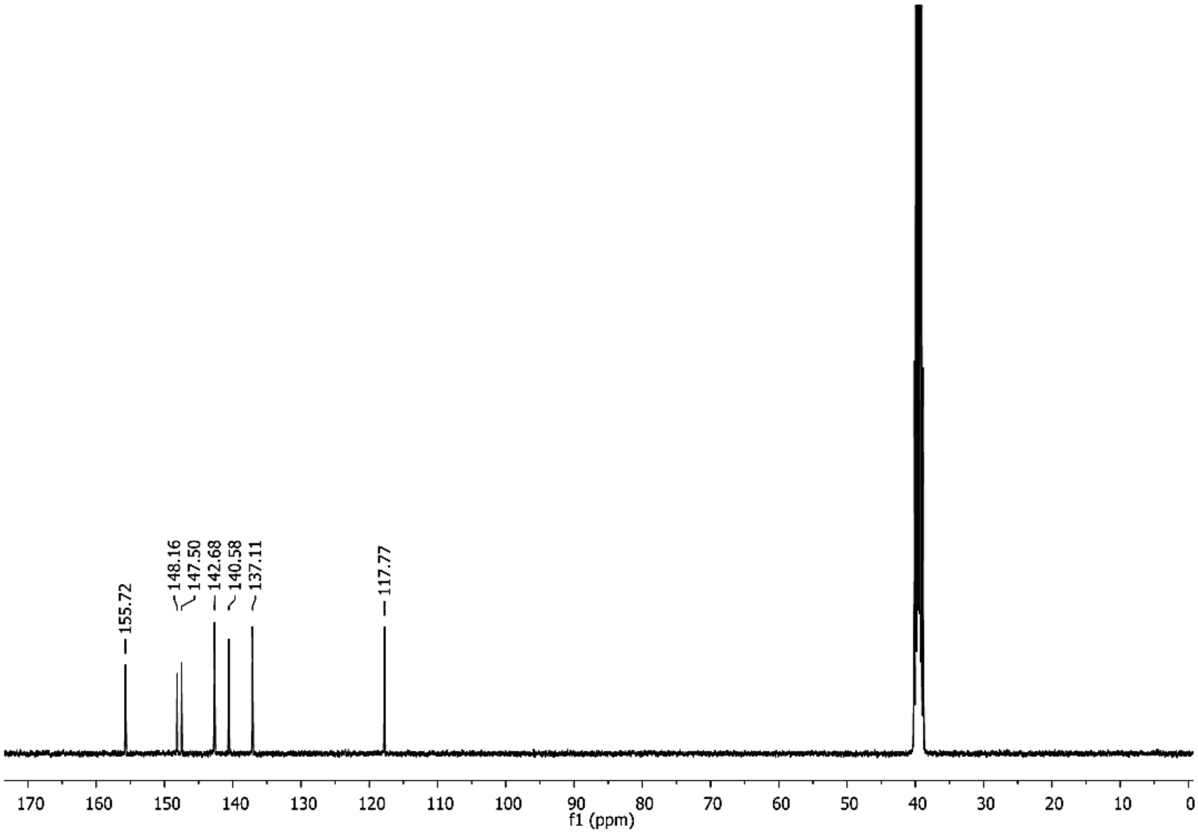

Figure S2. ^{13}C NMR spectrum of N^{2},N^{5}-di(pyrazin-2-yl)furan-2,5-dicarboxamide (**1**).

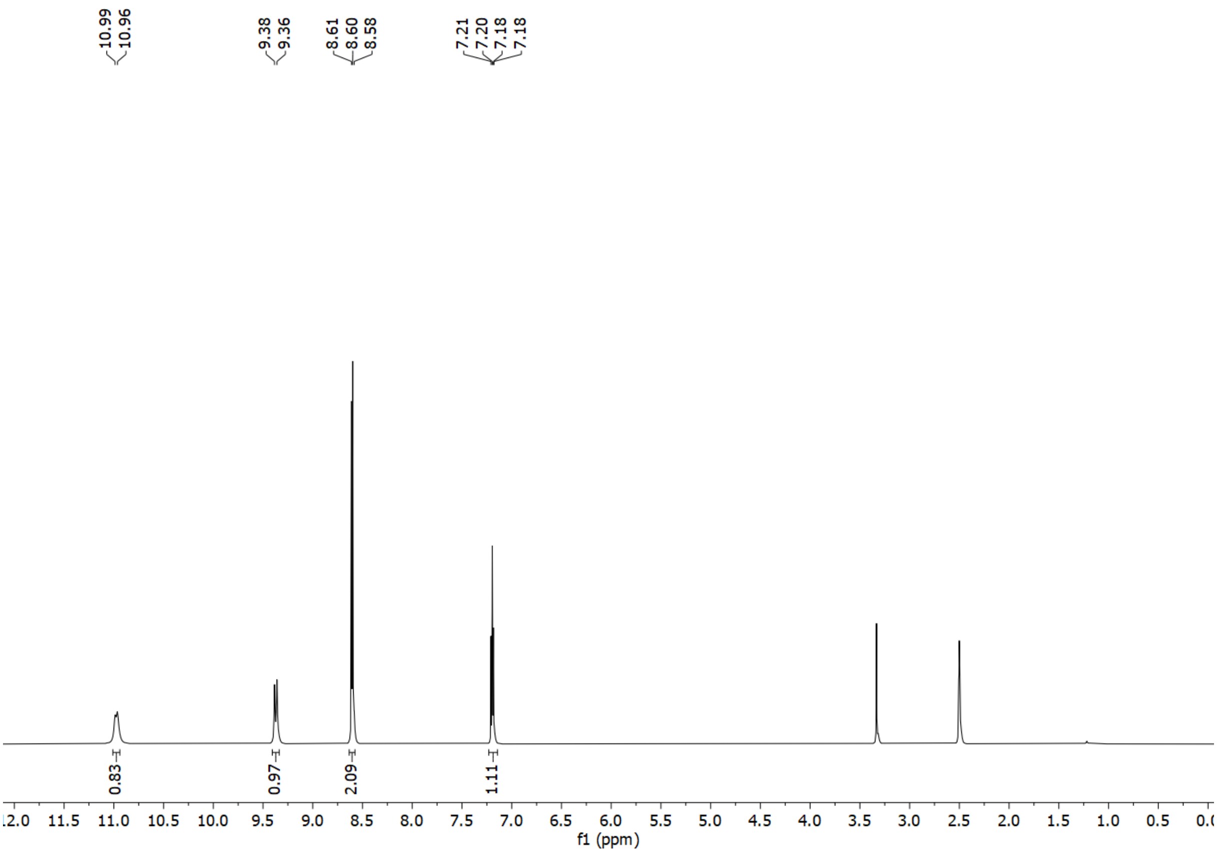

Figure S3. ^{1}H NMR spectrum of *N*[2],*N*[5]-di(pyrimidin-2-yl)furan-2,5-dicarboxamide (**2**).

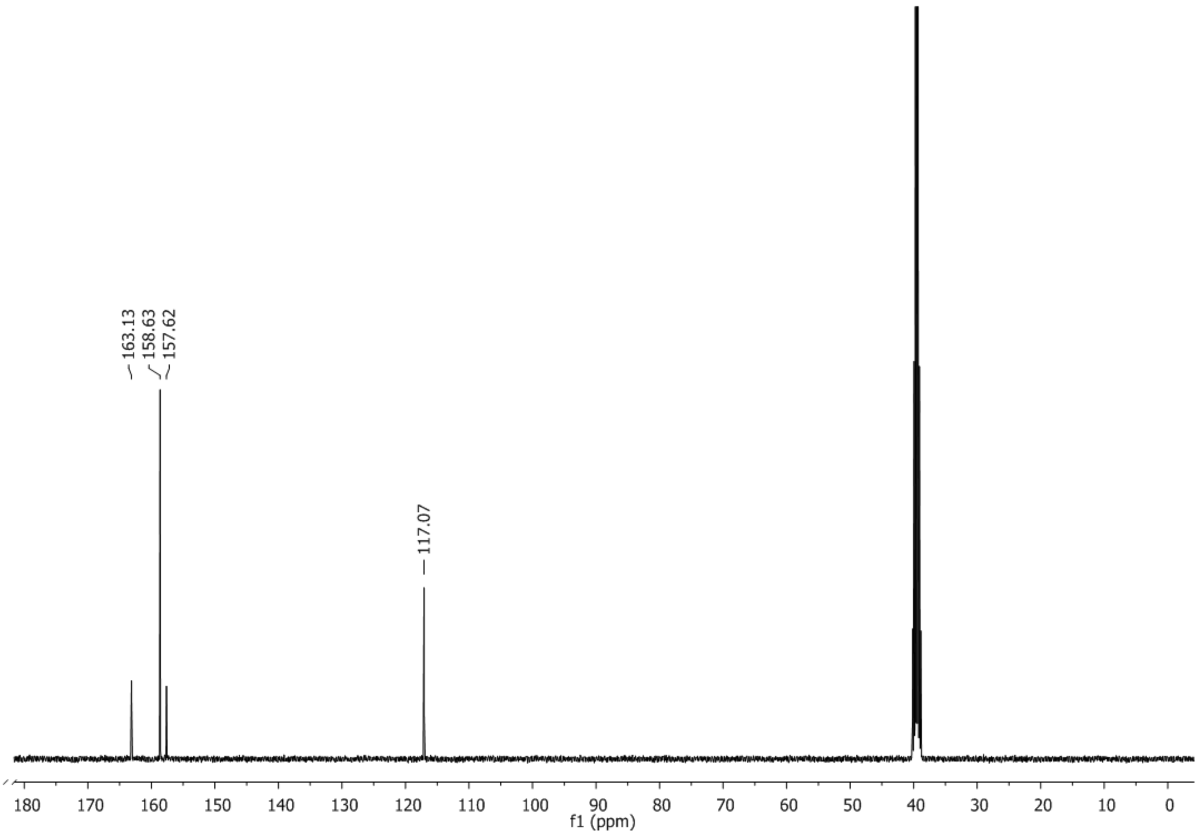

Figure S4. ^{13}C NMR spectrum of *N*[2],*N*[5]-di(pyrimidin-2-yl)furan-2,5-dicarboxamide (**2**).

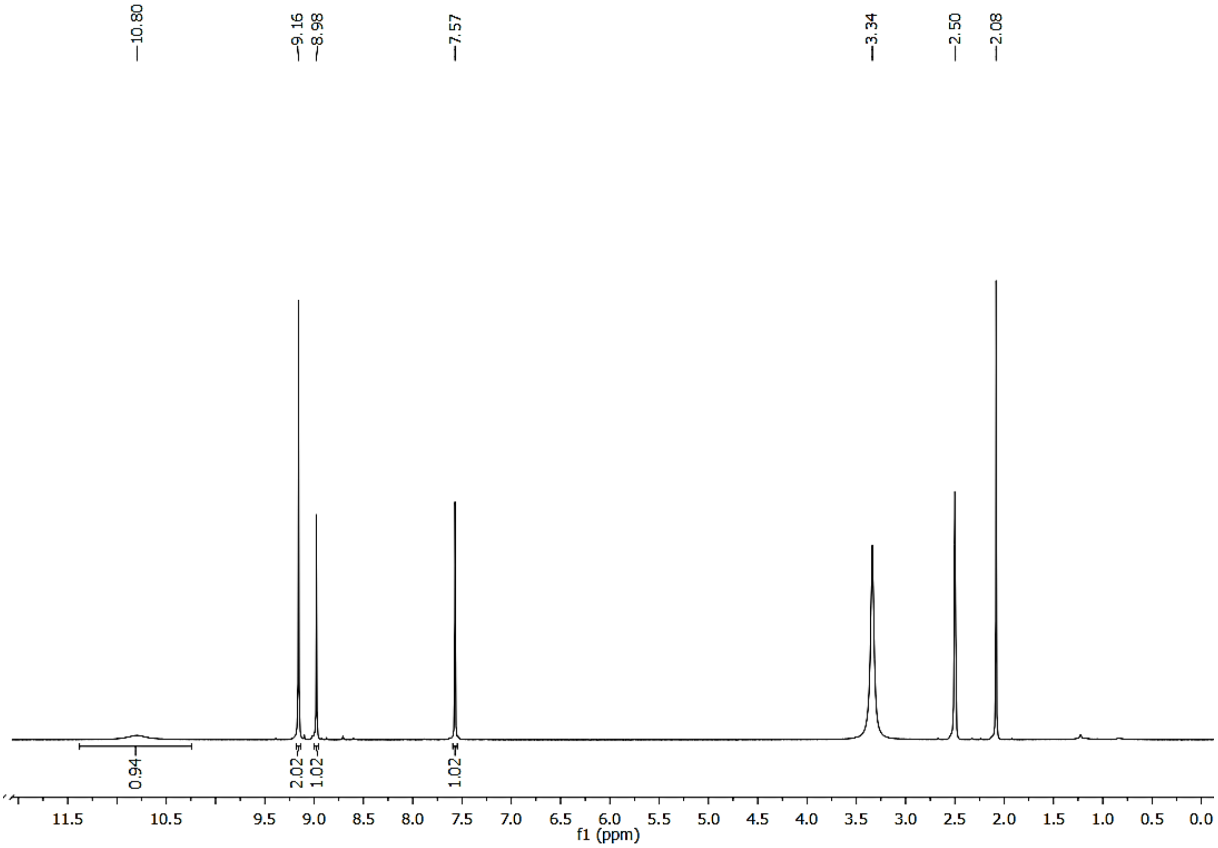

Figure S5. ^{1}H NMR spectrum of N^2,N^5-di(pyrimidin-5-yl)furan-2,5-dicarboxamide (**3**).

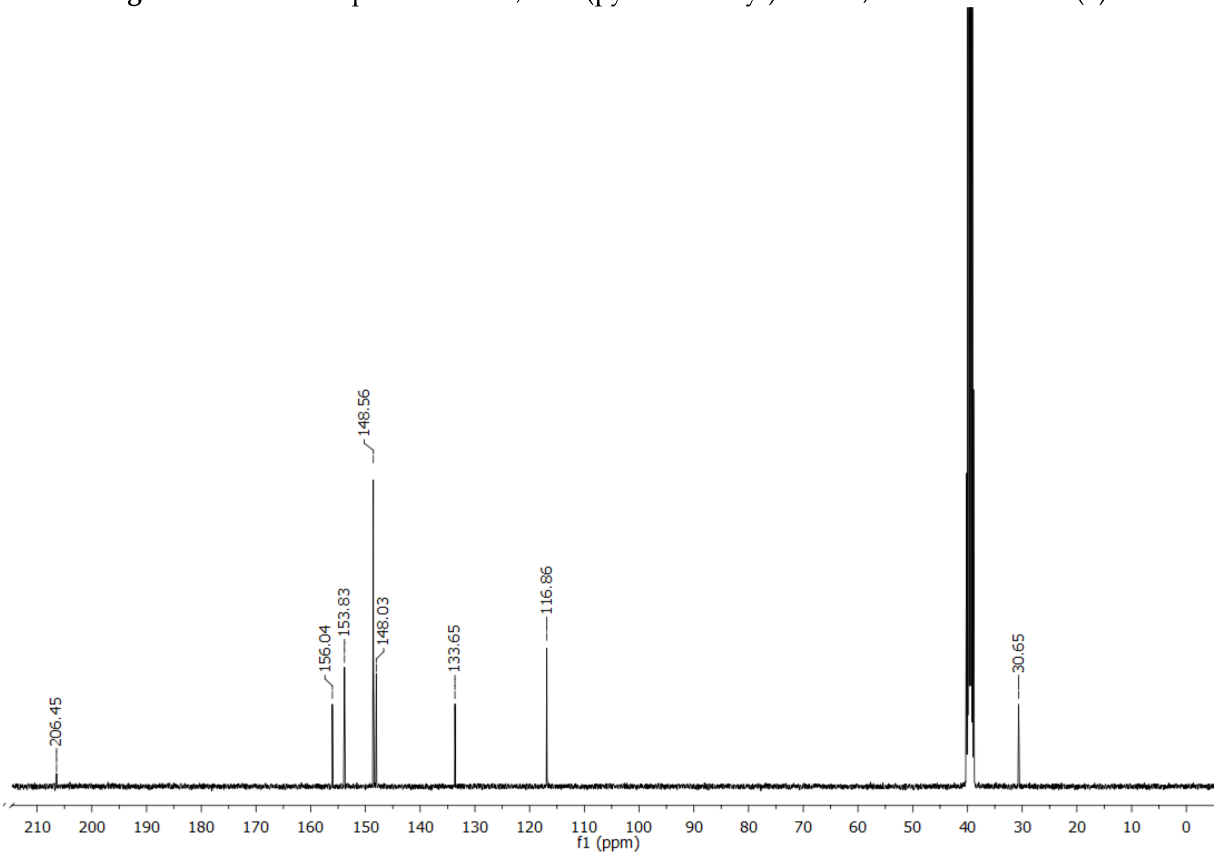

Figure S6. ^{13}C NMR spectrum of N^2,N^5-di(pyrimidin-5-yl)furan-2,5-dicarboxamide (**3**).

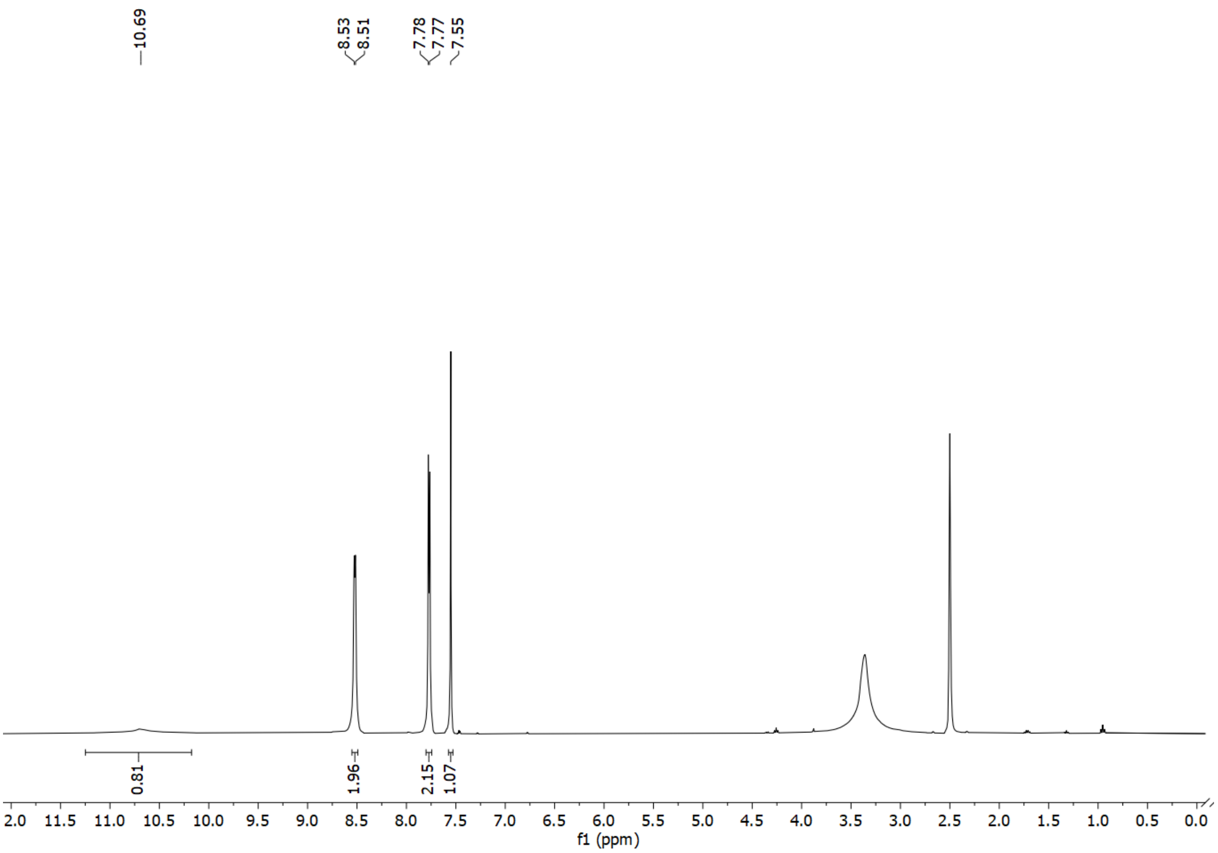

Figure S7. ^{1}H NMR spectrum of N^2,N^5-di(pyridin-4-yl)furan-2,5-dicarboxamide (**4**).

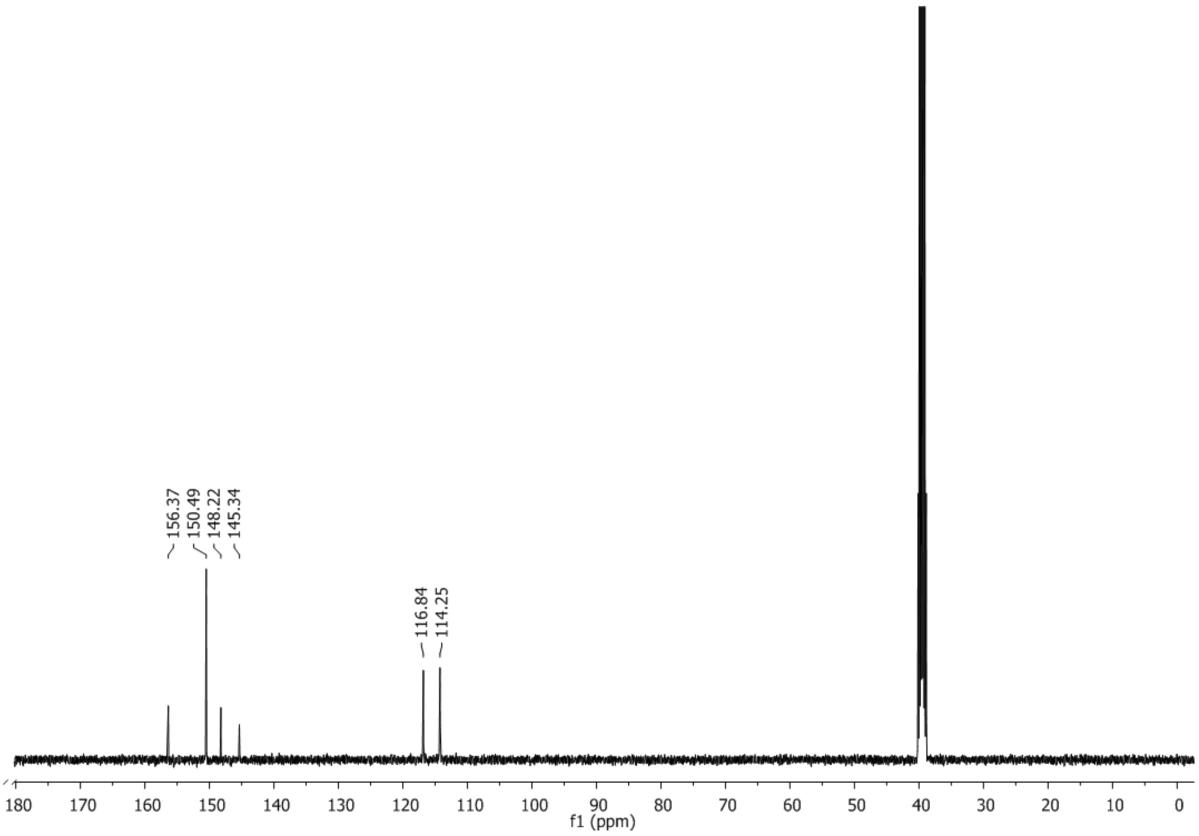

Figure S8. ^{13}C NMR spectrum of N^2,N^5-di(pyridin-4-yl)furan-2,5-dicarboxamide (**4**).

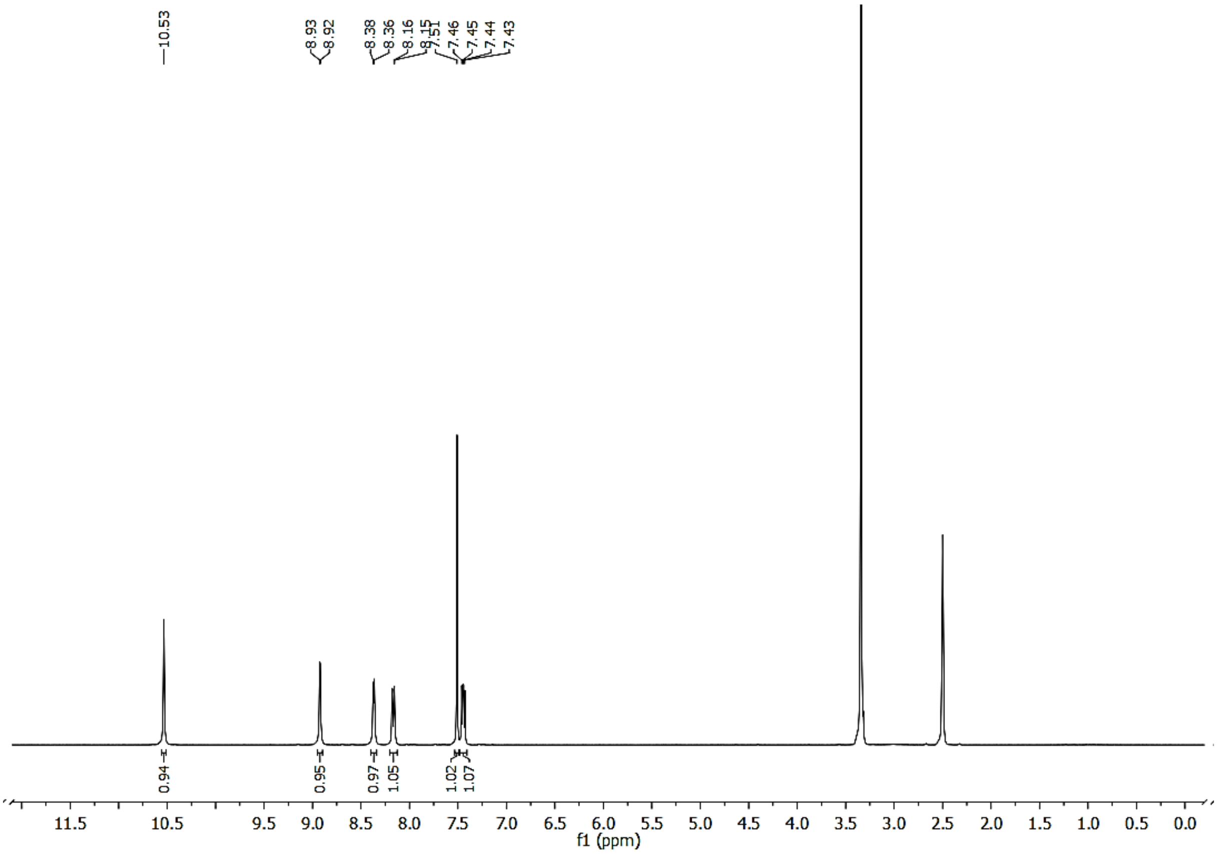

Figure S9. ^{1}H NMR spectrum of *N*2,*N*5-di(pyridin-3-yl)furan-2,5-dicarboxamide (**5**).

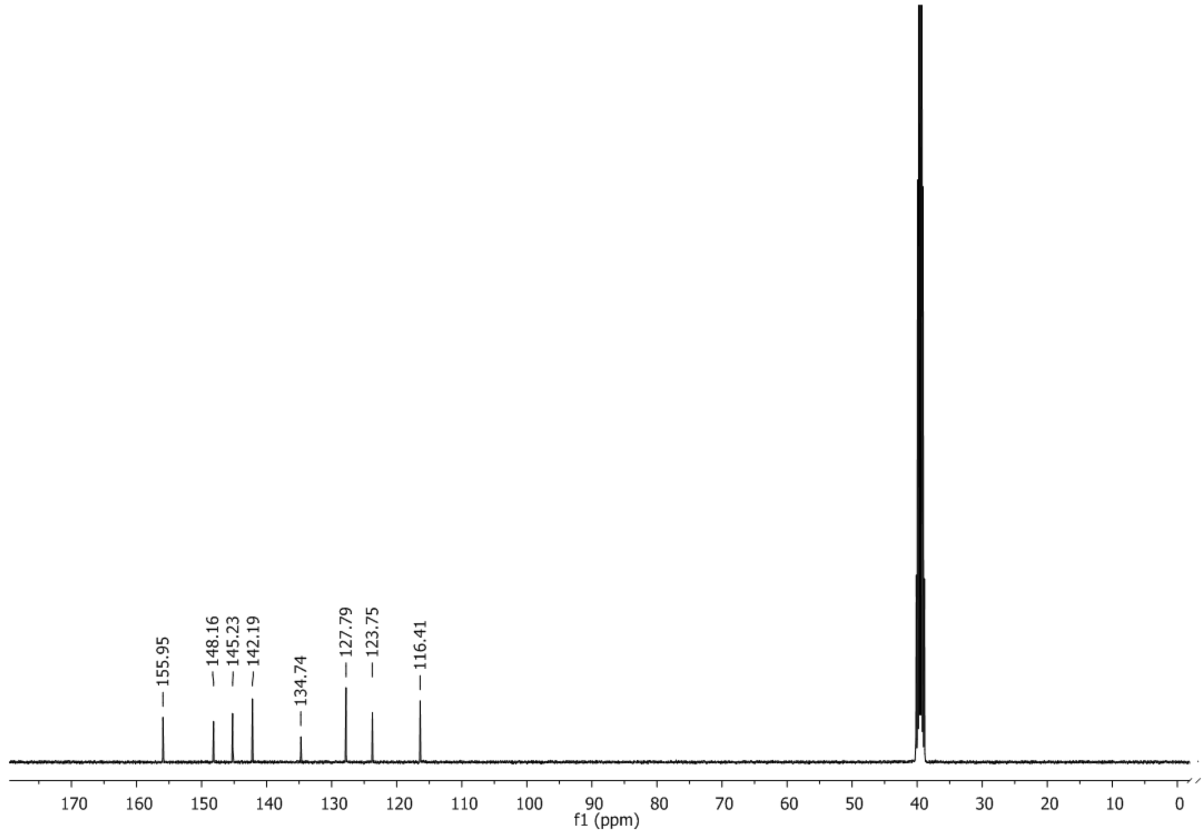

Figure S10. ^{13}C NMR spectrum of *N*2,*N*5-di(pyridin-3-yl)furan-2,5-dicarboxamide (**5**).

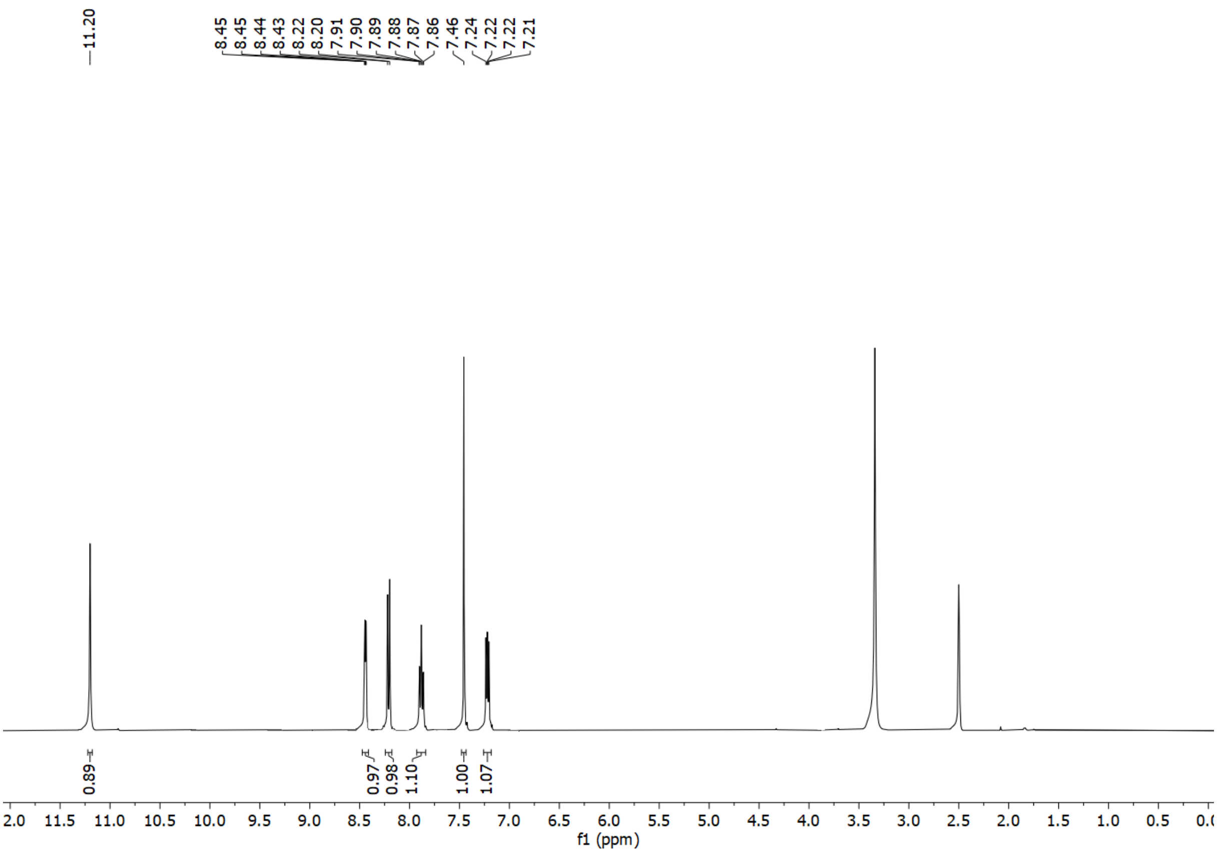

Figure S11. ^{1}H NMR spectrum of N^2,N^5-di(pyridin-2-yl)furan-2,5-dicarboxamide (**6**).

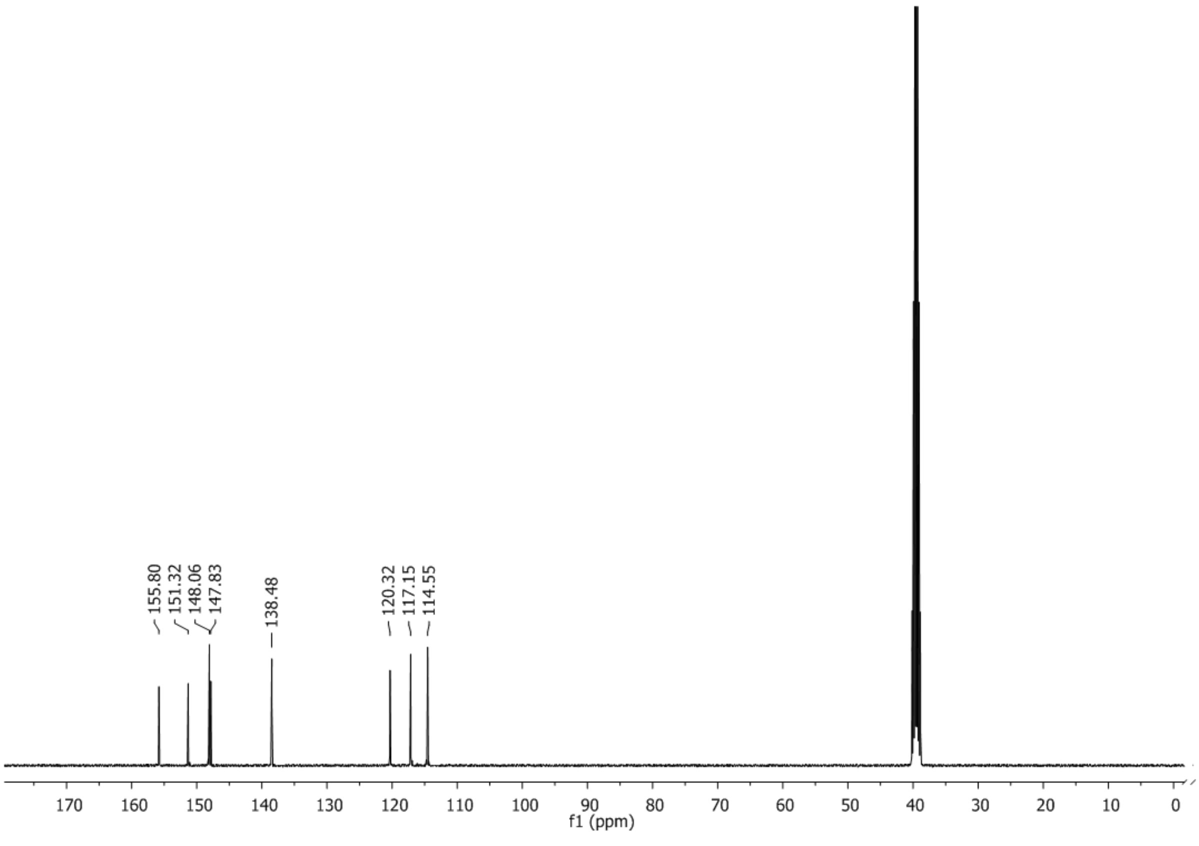

Figure S12. ^{13}C NMR spectrum of N^2,N^5-di(pyridin-2-yl)furan-2,5-dicarboxamide (**6**).

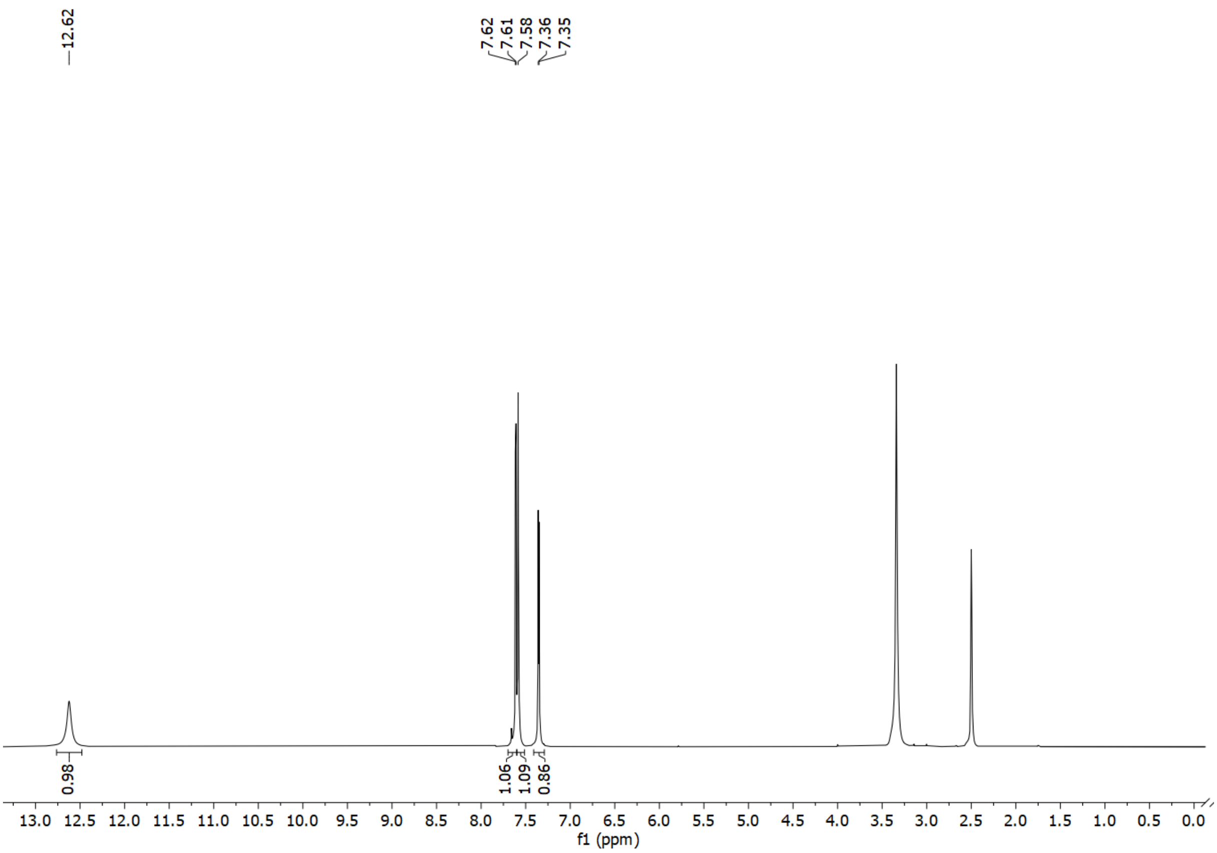

Figure S13. ^{1}H NMR spectrum of N^2,N^5-di(1,3-thiazol-2-yl)furan-2,5-dicarboxamide (7).

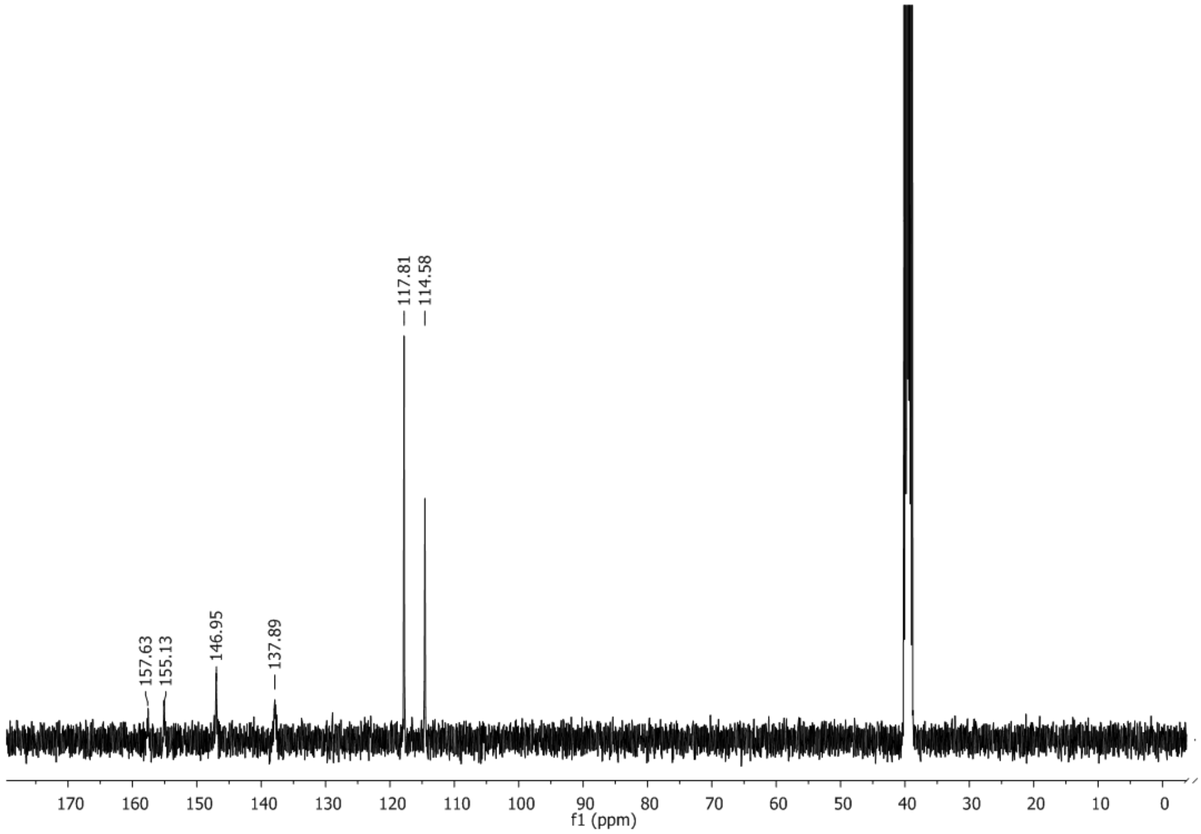

Figure S14. ^{13}C NMR spectrum of N^2,N^5-di(1,3-thiazol-2-yl)furan-2,5-dicarboxamide (**7**).

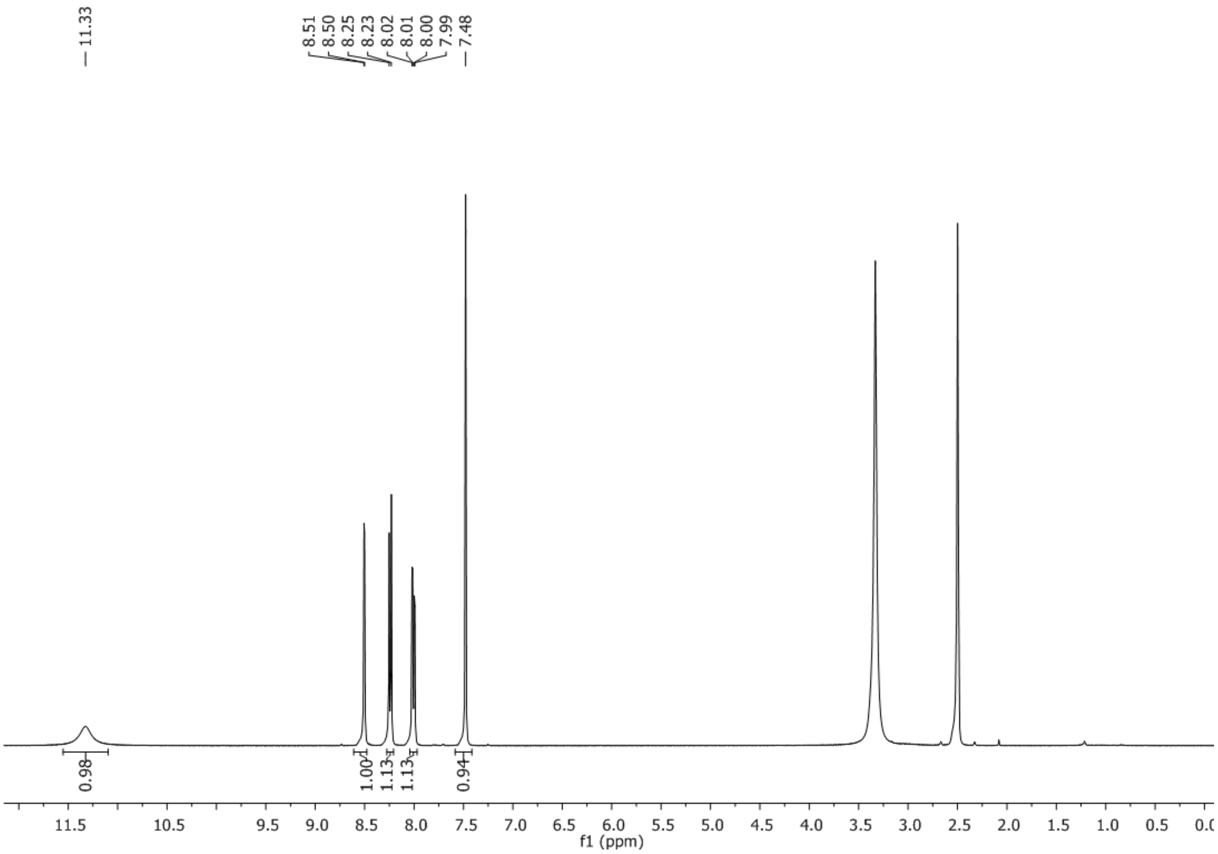

Figure S15. ^{1}H NMR spectrum of N^2,N^5-bis(5-chloropyridin-2-yl)furan-2,5-dicarboxamide (**8**).

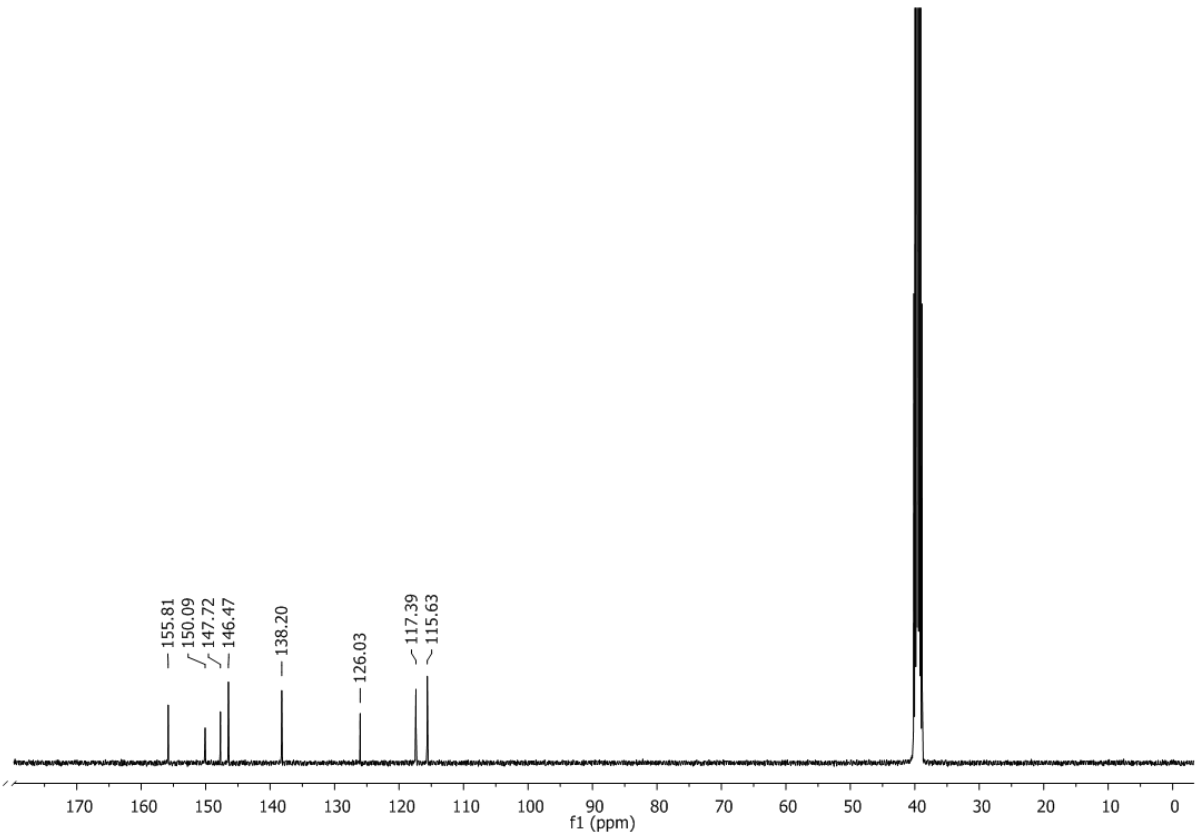

Figure S16. ^{13}C NMR spectrum of N^2,N^5-bis(5-chloropyridin-2-yl)furan-2,5-dicarboxamide (**8**).

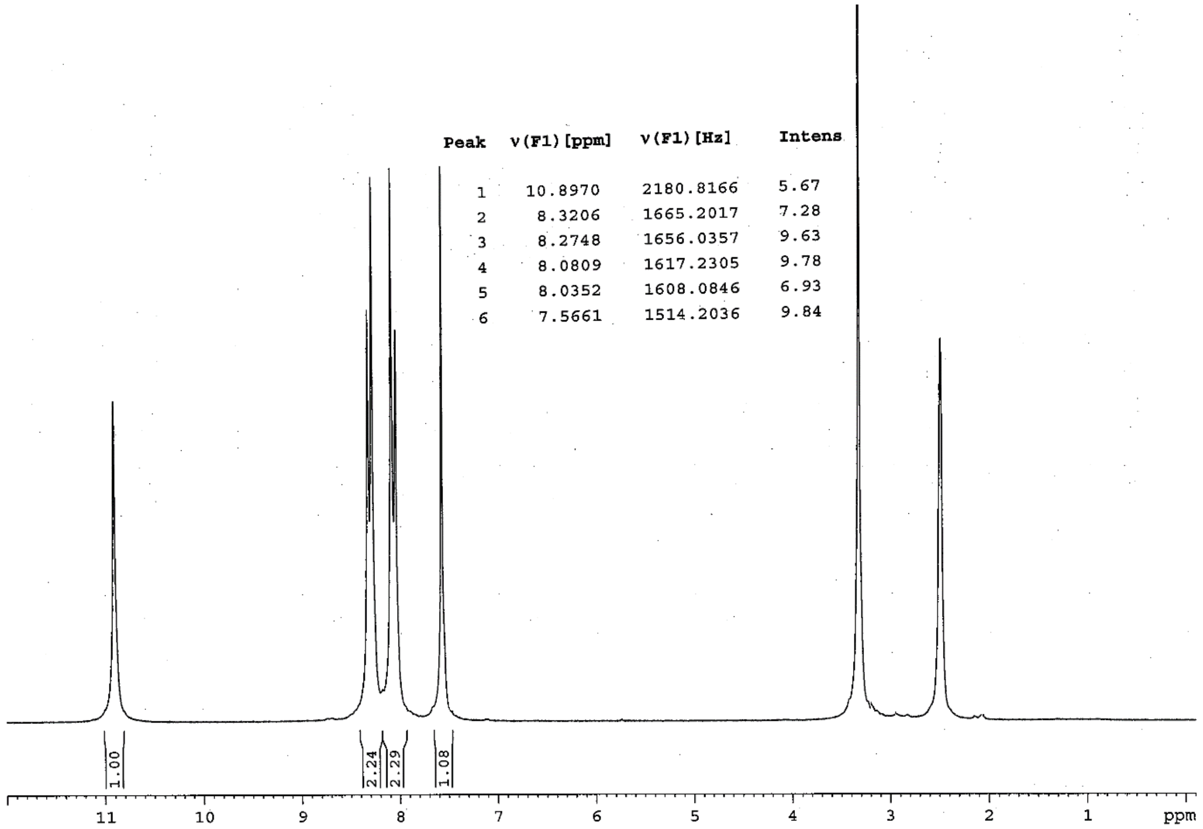

Figure S17. ^{1}H NMR spectrum of *N*[2],*N*[5]-di(4-nitrophenyl)furan-2,5-dicarboxamide (**9**).

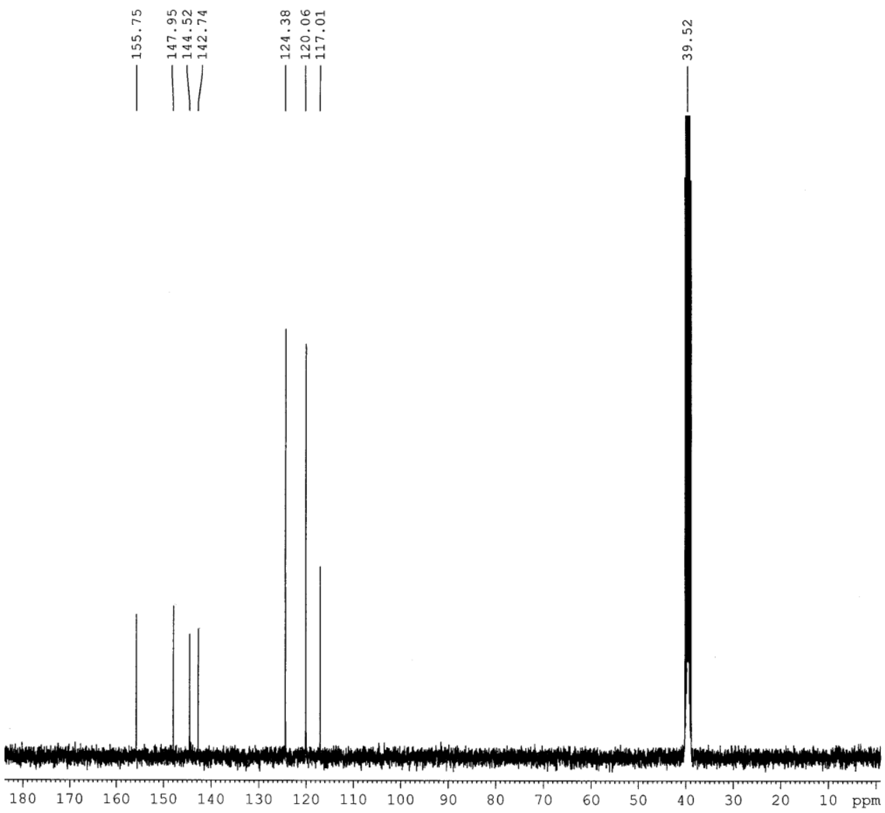

Figure S18. ^{13}C NMR spectrum of *N*[2],*N*[5]-di(4-nitrophenyl)furan-2,5-dicarboxamide (**9**).

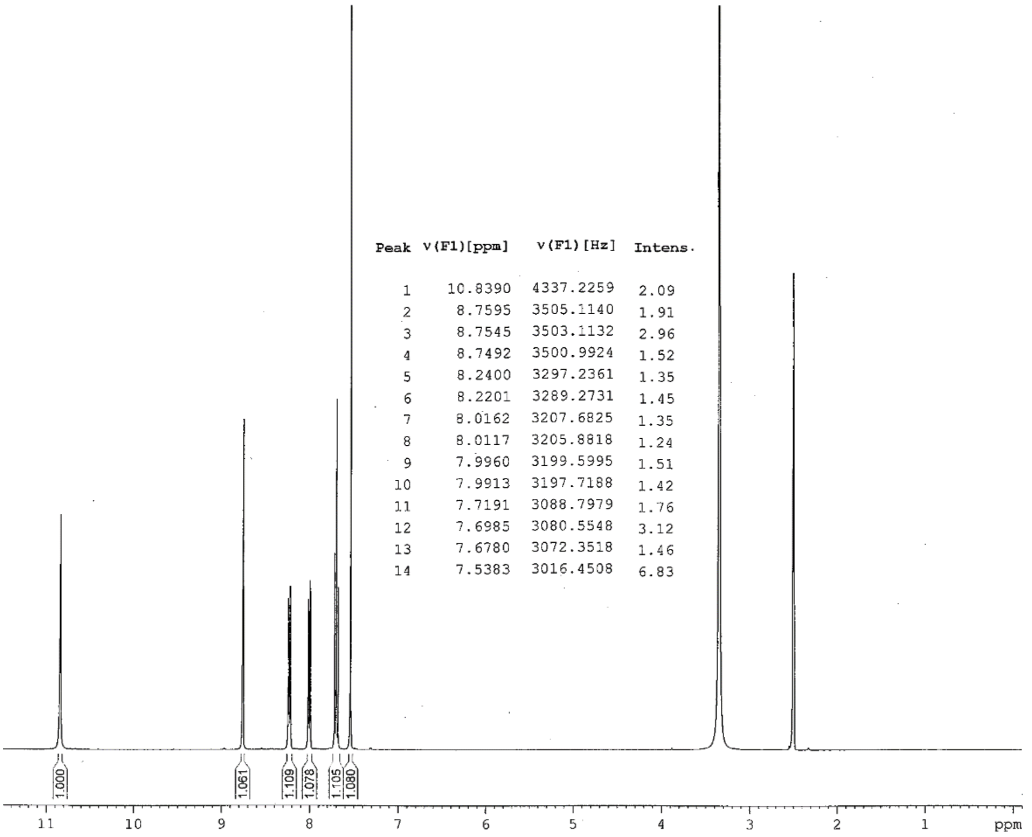

Figure S19. ^{1}H NMR spectrum of *N*[2],*N*[5]-di(3-nitrophenyl)furan-2,5-dicarboxamide (**10**).

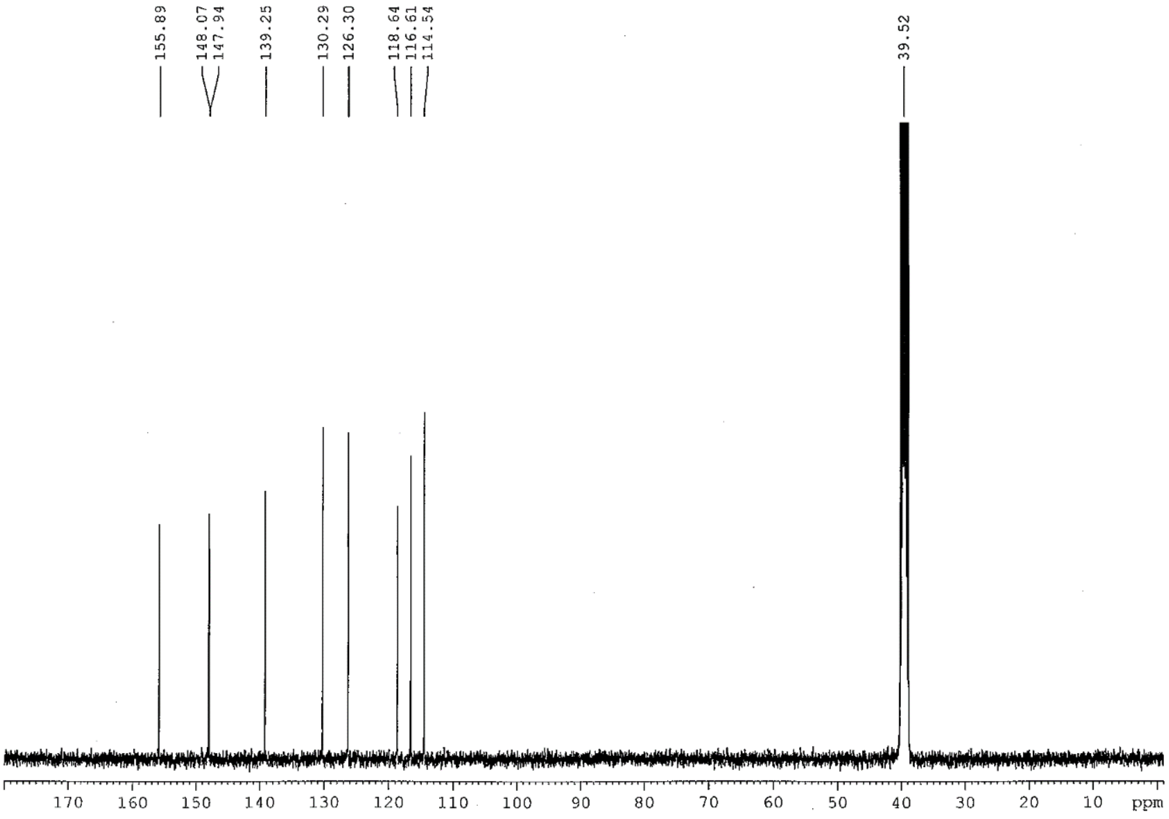

Figure S20. ^{13}C NMR spectrum of *N*[2],*N*[5]-di(3-nitrophenyl)furan-2,5-dicarboxamide (**10**).

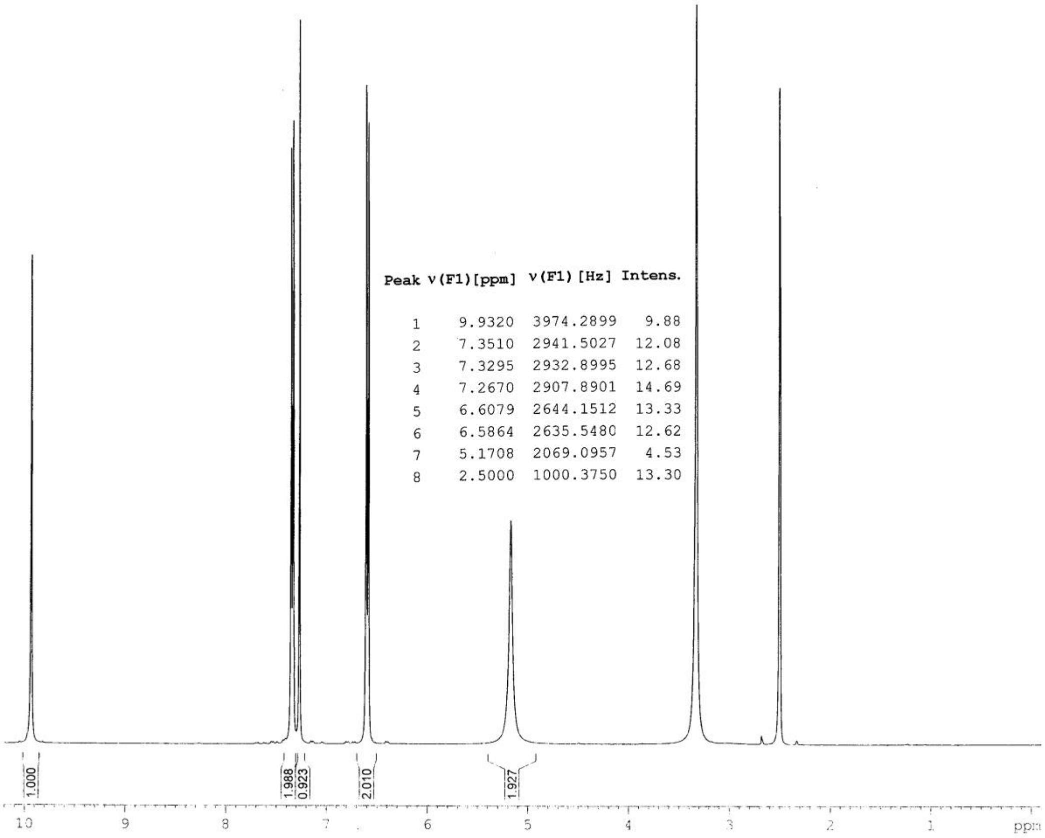

Figure S21. ^{1}H NMR spectrum of *N*[2],*N*[5]-di(4-aminophenyl)furan-2,5-dicarboxamide (**11**).

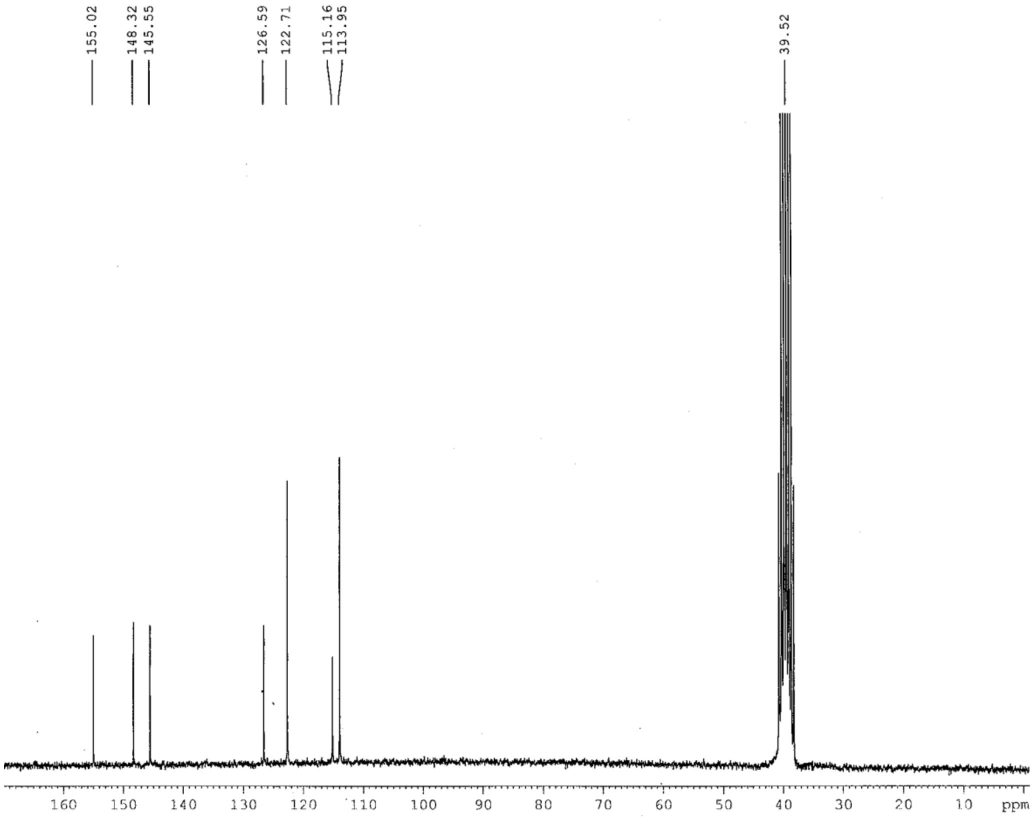

Figure S22. ^{13}C NMR spectrum of *N*[2],*N*[5]-di(4-aminophenyl)furan-2,5-dicarboxamide (**11**).

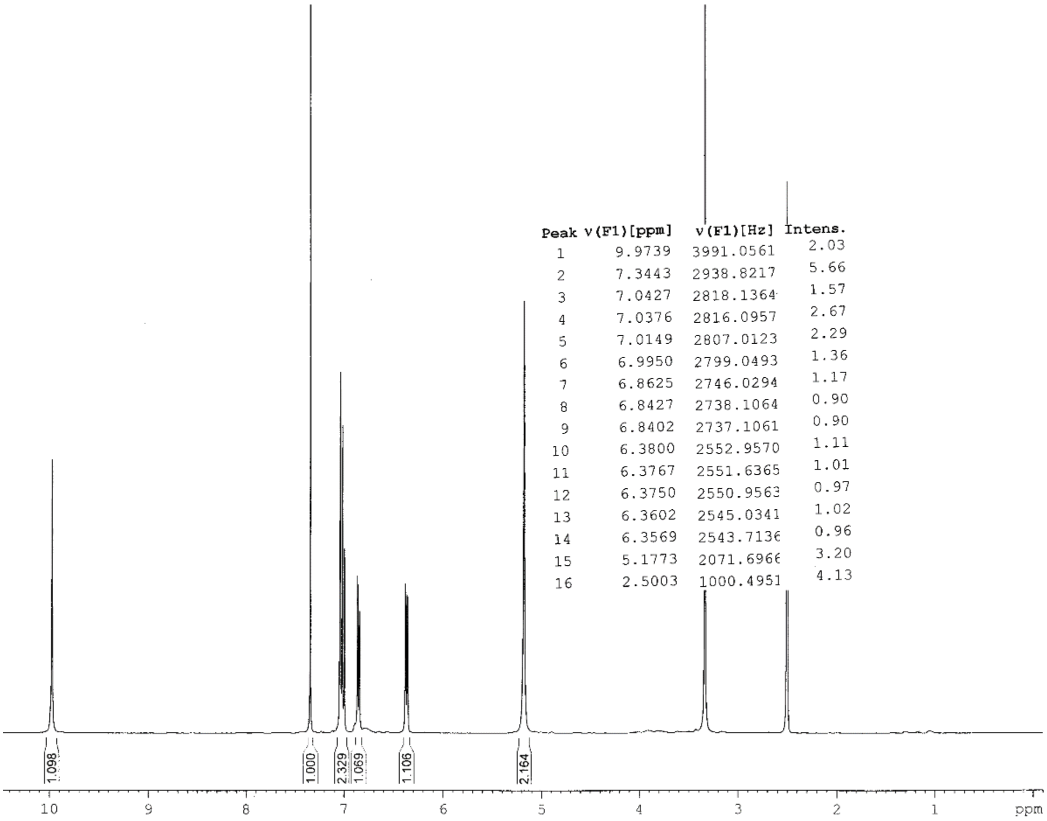

Figure S23. ^{1}H NMR spectrum of *N*^{2},*N*^{5}-di(3-aminophenyl)furan-2,5-dicarboxamide (**12**).

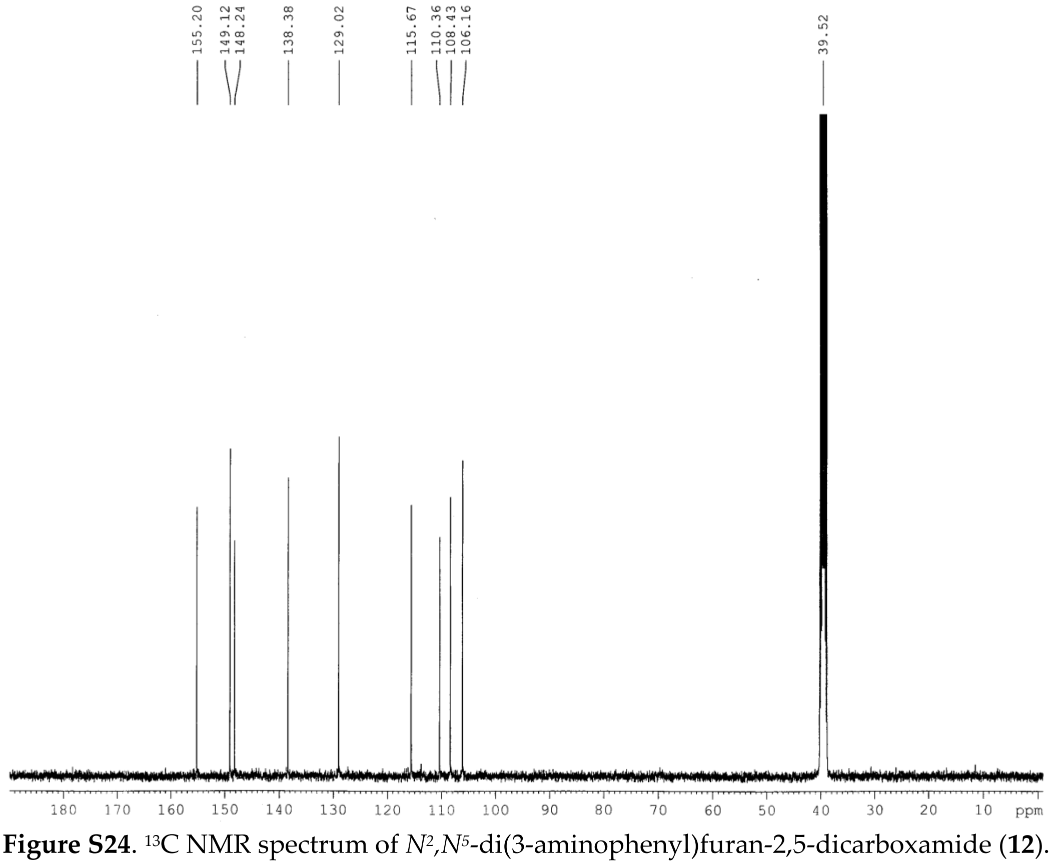

Figure S24. ^{13}C NMR spectrum of *N*^{2},*N*^{5}-di(3-aminophenyl)furan-2,5-dicarboxamide (**12**).

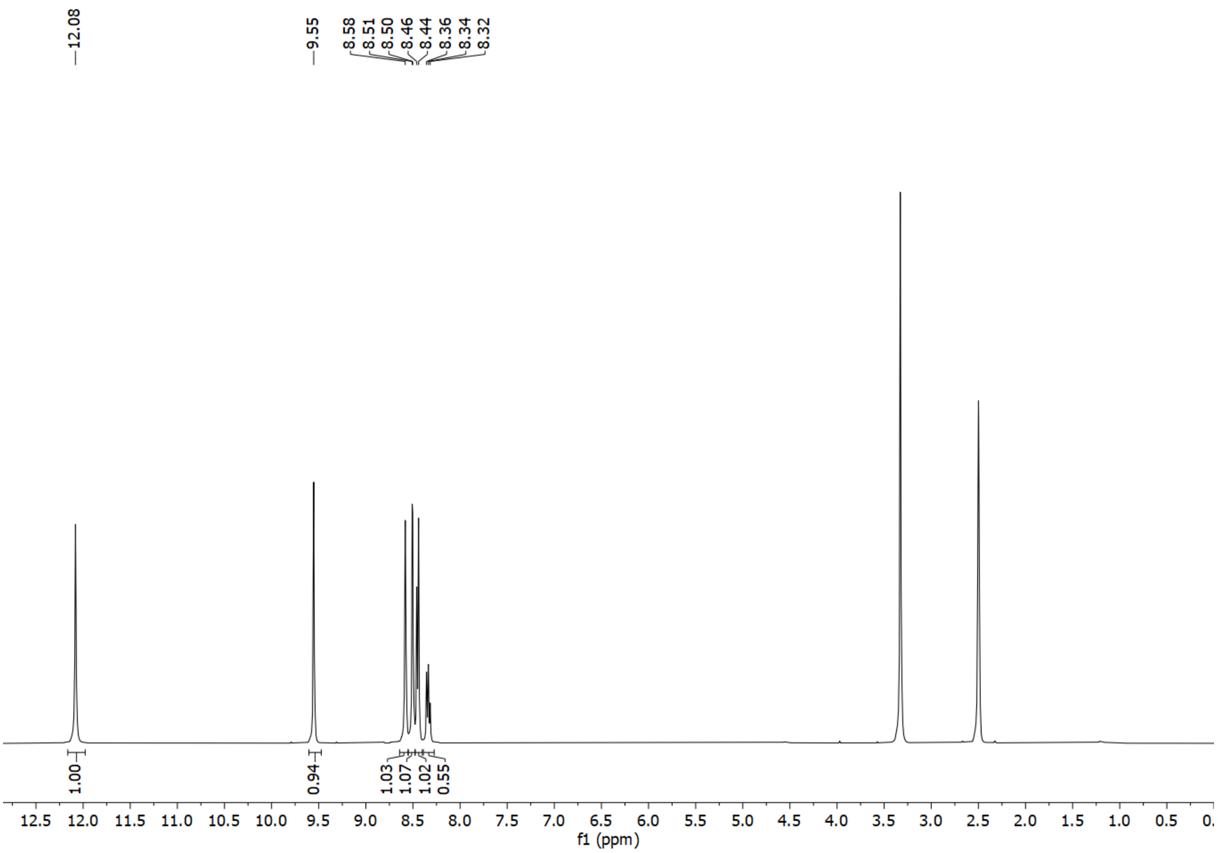

Figure S25. ^{1}H NMR spectrum of N^2,N^6-di(pyrazin-2-yl)pyridine-2,6-dicarboxamide (**13**).

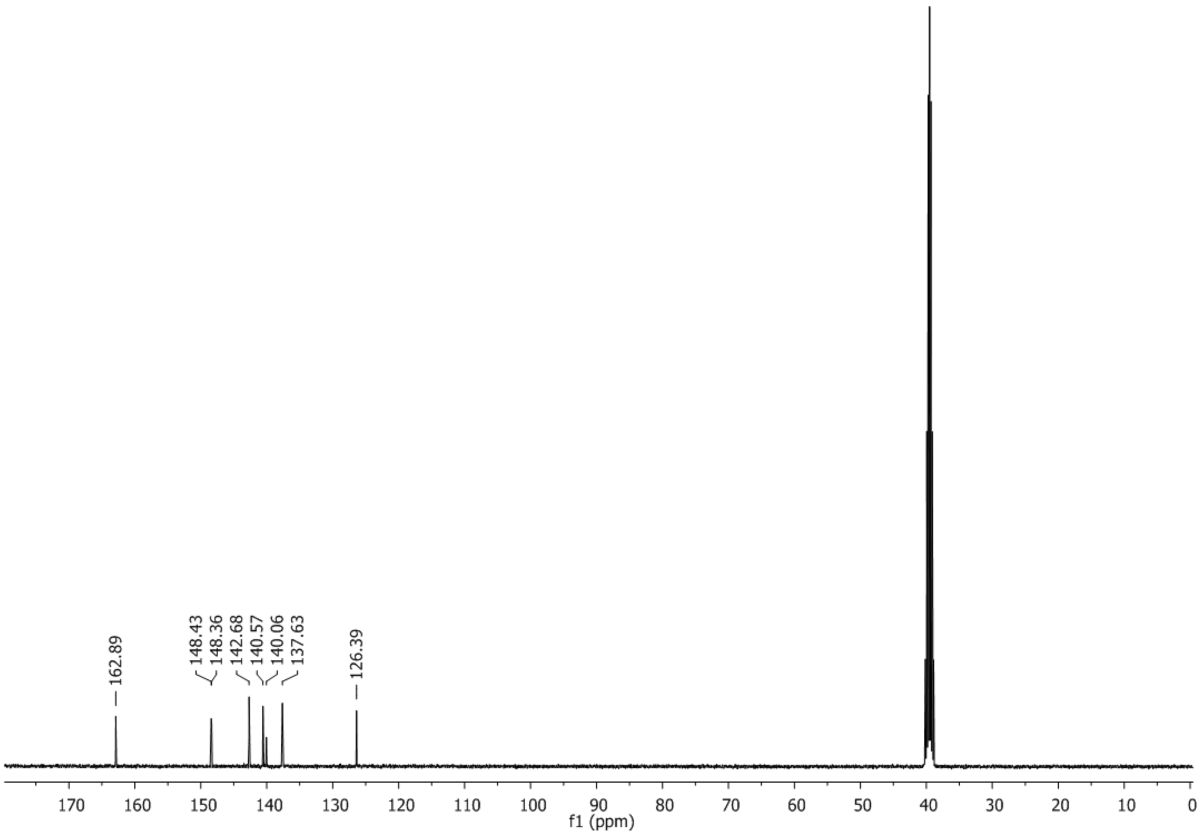

Figure S26. ^{13}C NMR spectrum of N^2,N^6-di(pyrazin-2-yl)pyridine-2,6-dicarboxamide (**13**).

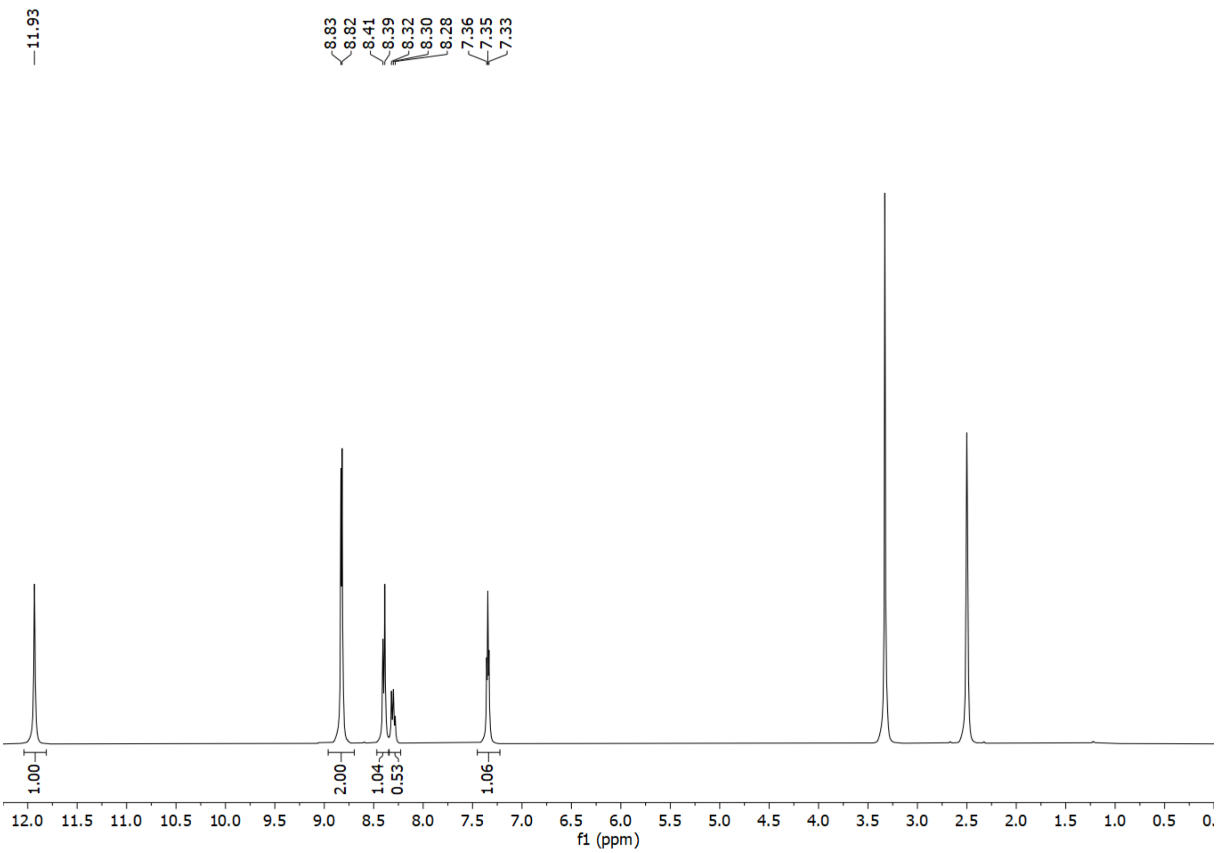

Figure S27. ^{1}H NMR spectrum of *N*[2],*N*[6]-di(pyrimidin-2-yl)pyridine-2,6-dicarboxamide (**14**).

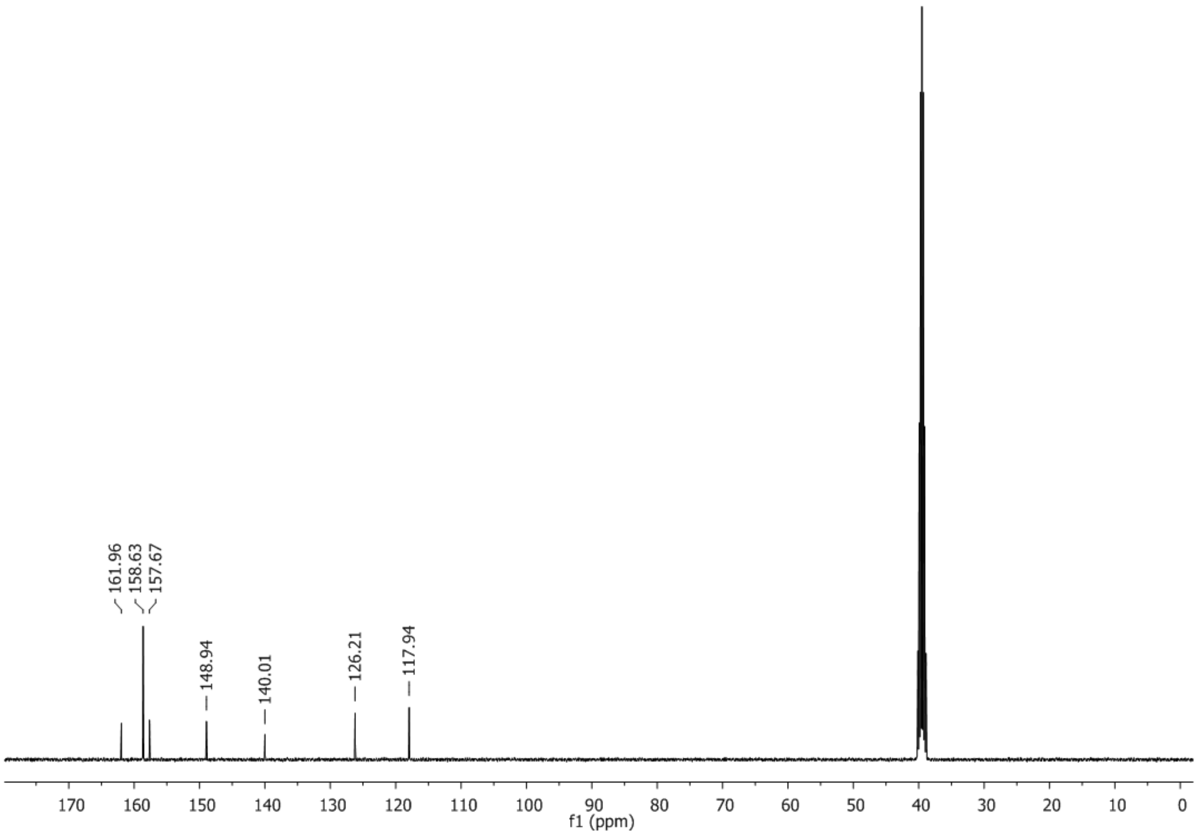

Figure S28. ^{13}C NMR spectrum of *N*[2],*N*[6]-di(pyrimidin-2-yl)pyridine-2,6-dicarboxamide (**14**).

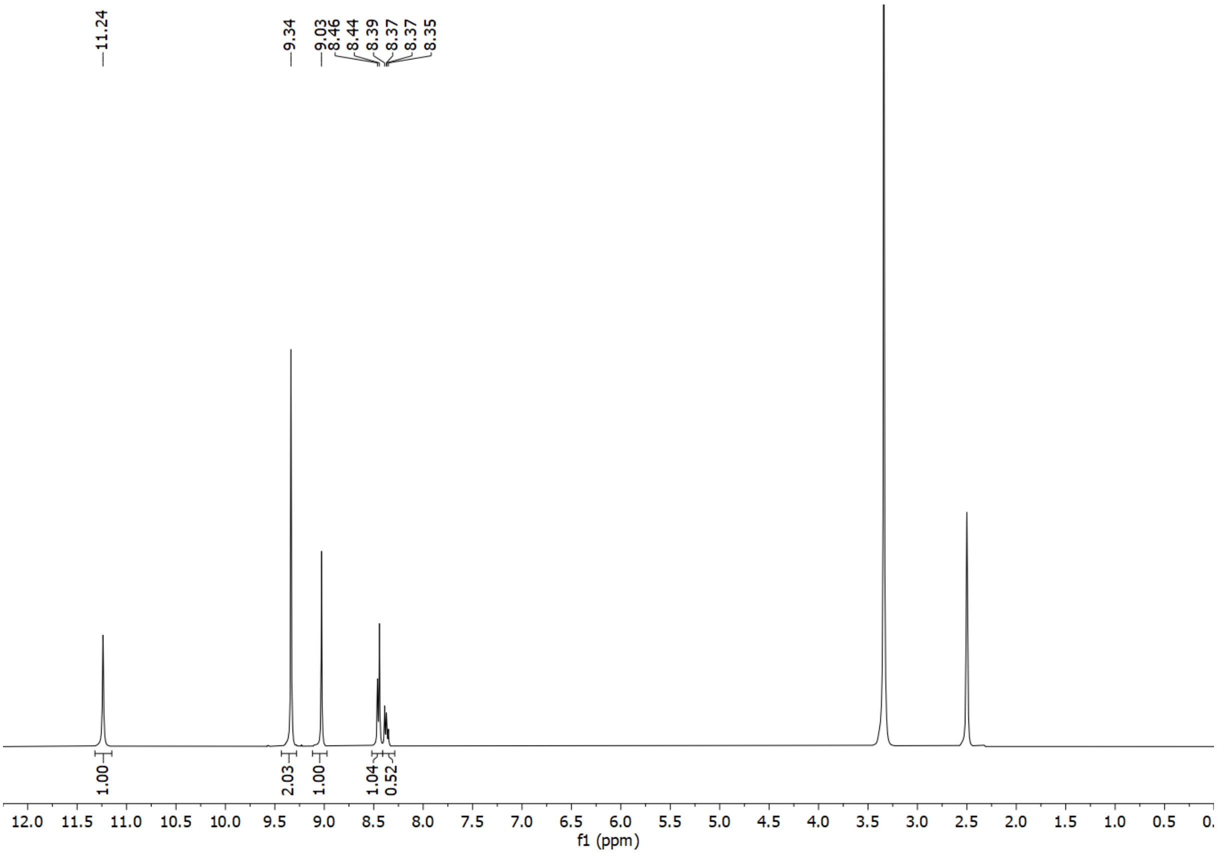

Figure S29. ^{1}H NMR spectrum of *N*^{2},*N*^{6}-di(pyrimidin-5-yl)pyridine-2,6-dicarboxamide (**15**).

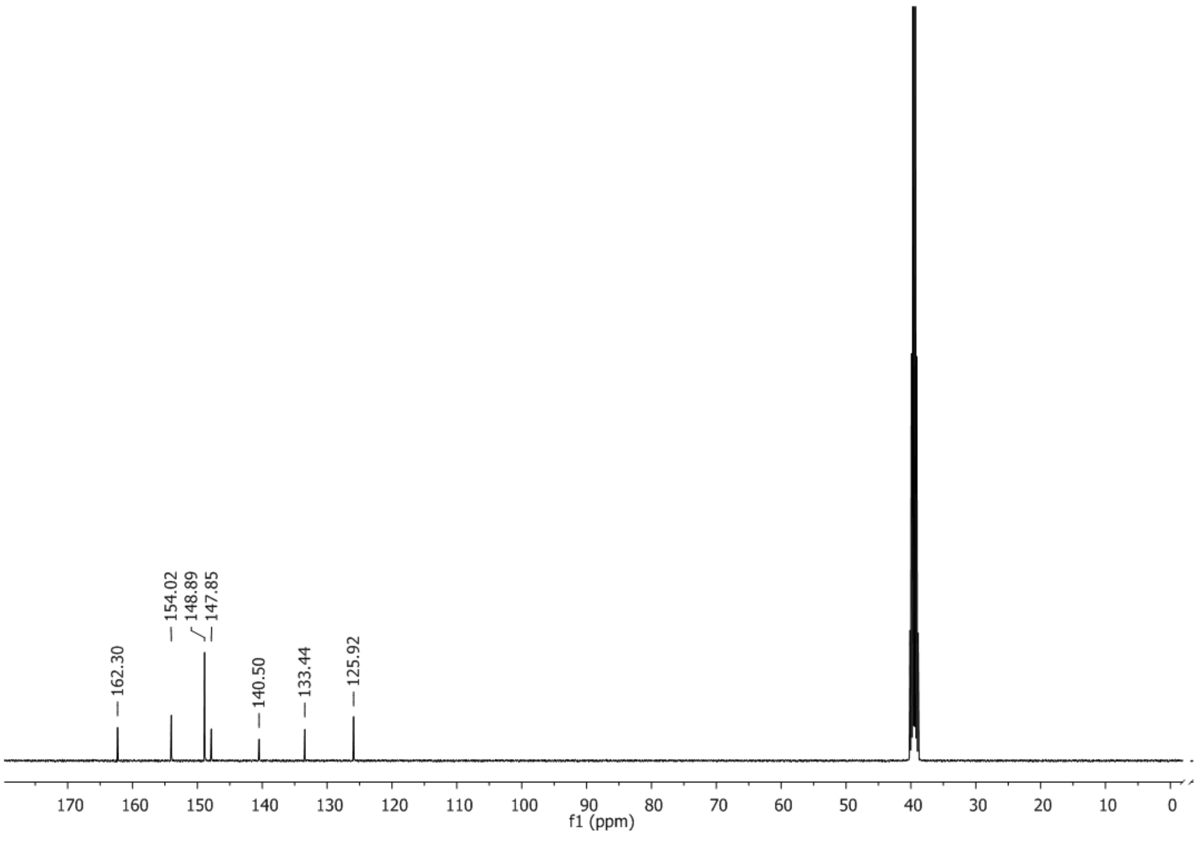

Figure S30. ^{13}C NMR spectrum of *N*^{2},*N*^{6}-di(pyrimidin-5-yl)pyridine-2,6-dicarboxamide (**15**).

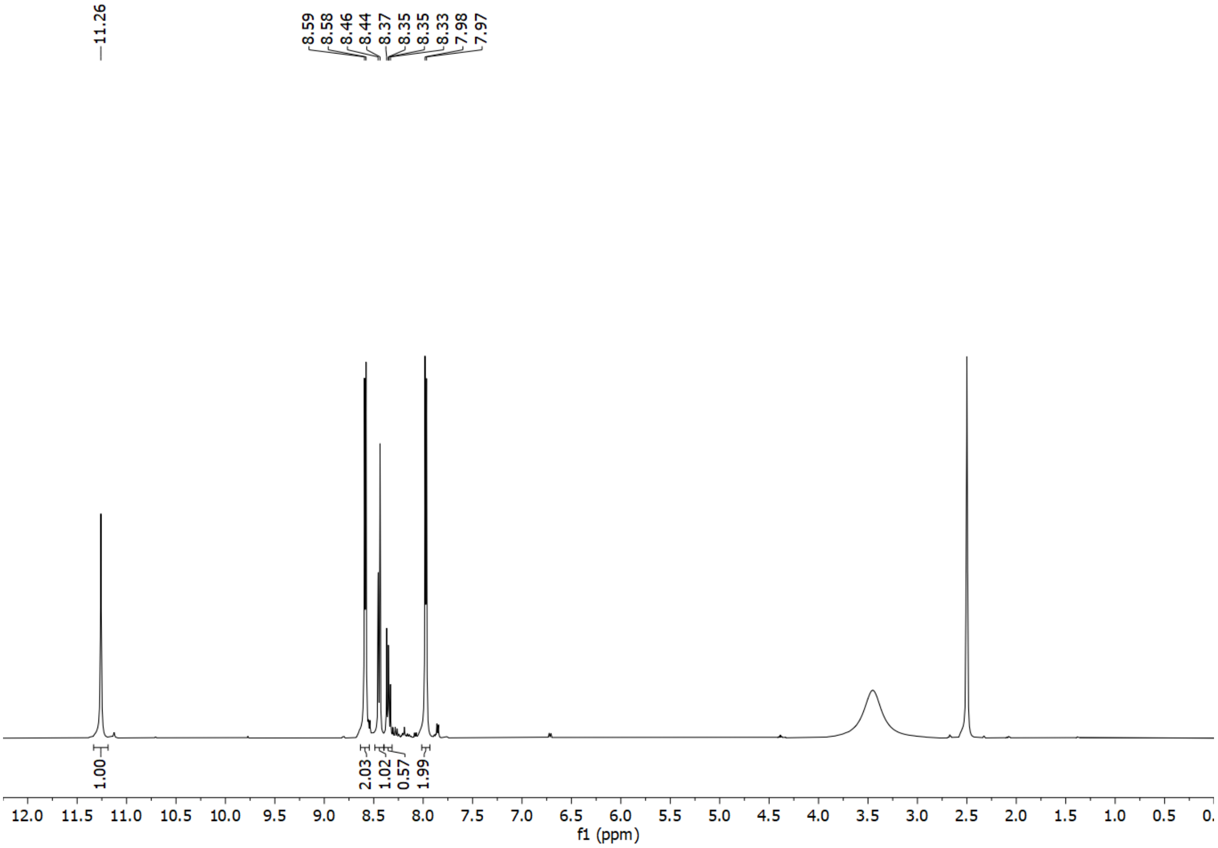

Figure S31. ^{1}H NMR spectrum of N^2,N^6-di(pyridin-4-yl)pyridine-2,6-dicarboxamide (**16**).

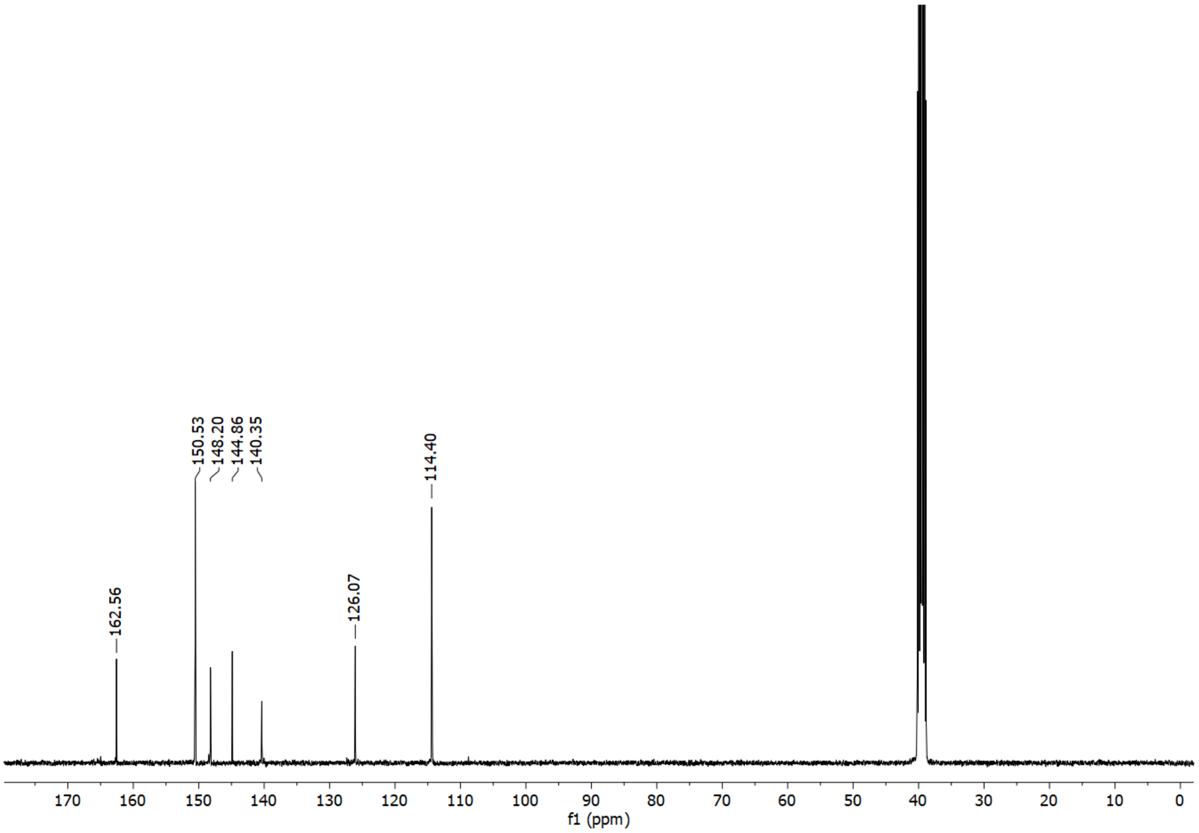

Figure S32. ^{13}C NMR spectrum of N^2,N^6-di(pyridin-4-yl)pyridine-2,6-dicarboxamide (**16**).

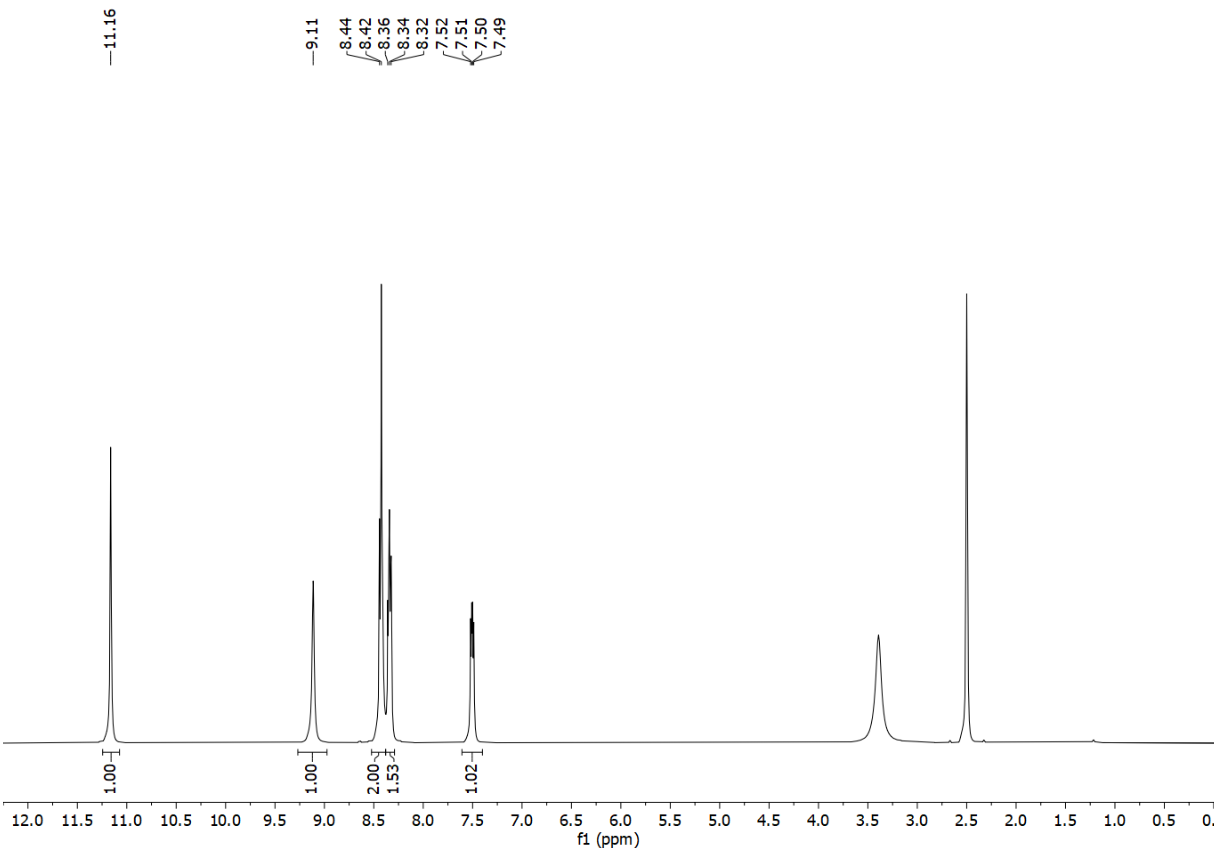

Figure S33. ¹H NMR spectrum of N^2,N^6-di(pyridin-3-yl)pyridine-2,6-dicarboxamide (**17**).

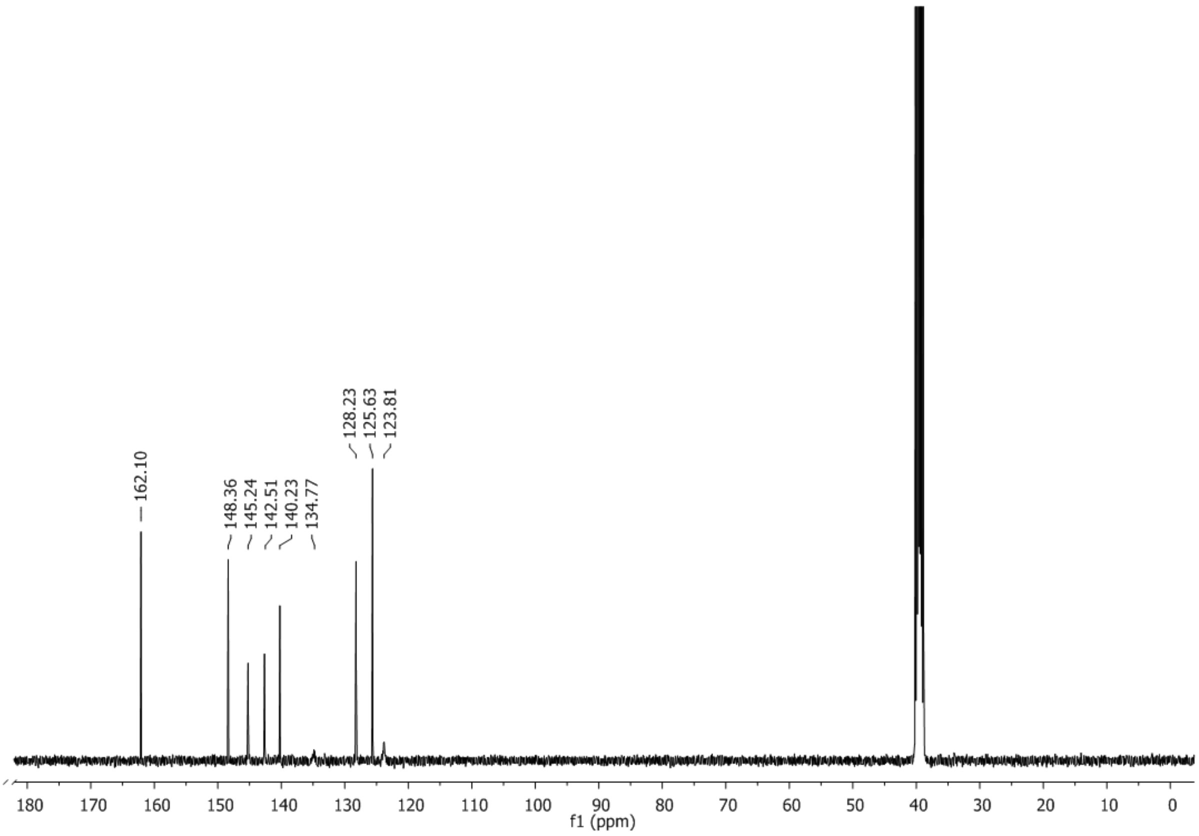

Figure S34. ¹³C NMR spectrum of N^2,N^6-di(pyridin-3-yl)pyridine-2,6-dicarboxamide (**17**).

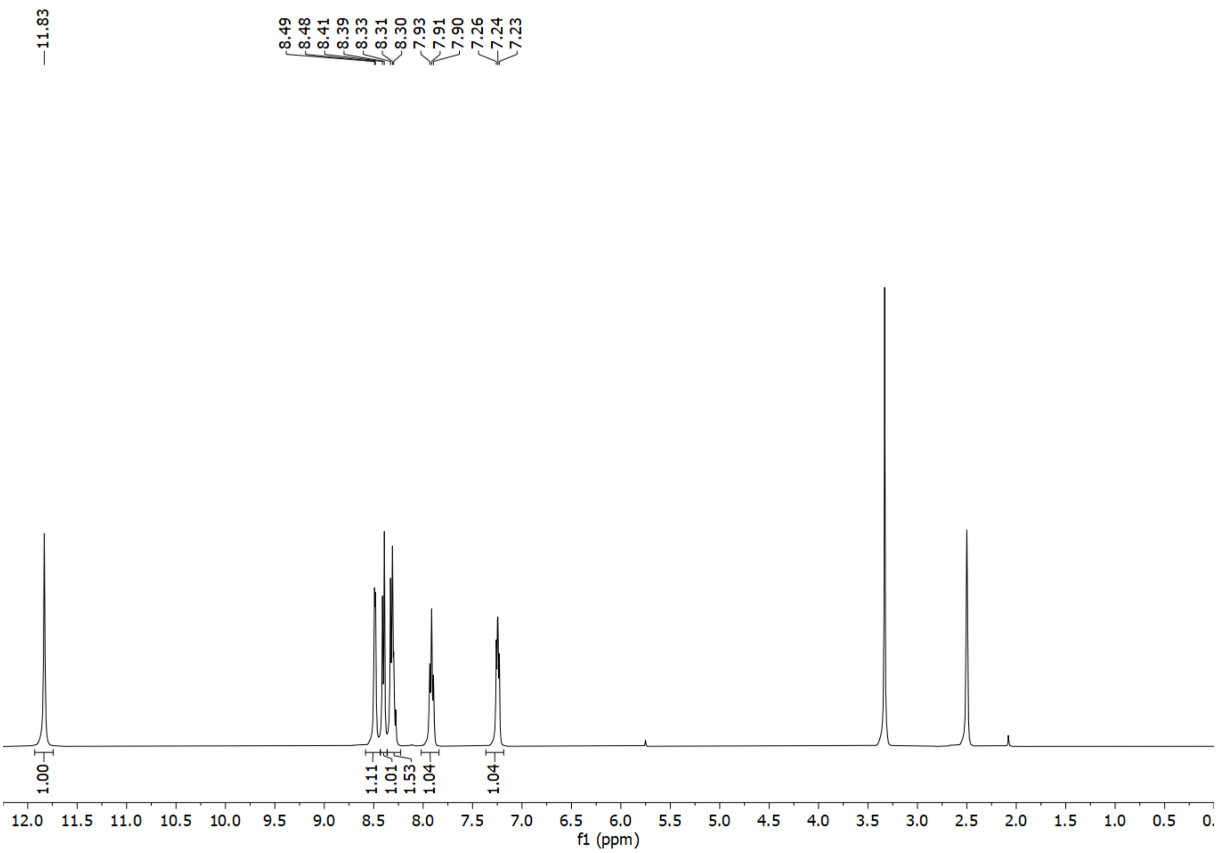

Figure S35. ^{1}H NMR spectrum of *N*[2],*N*[6]-di(pyridin-2-yl)pyridine-2,6-dicarboxamide (**18**).

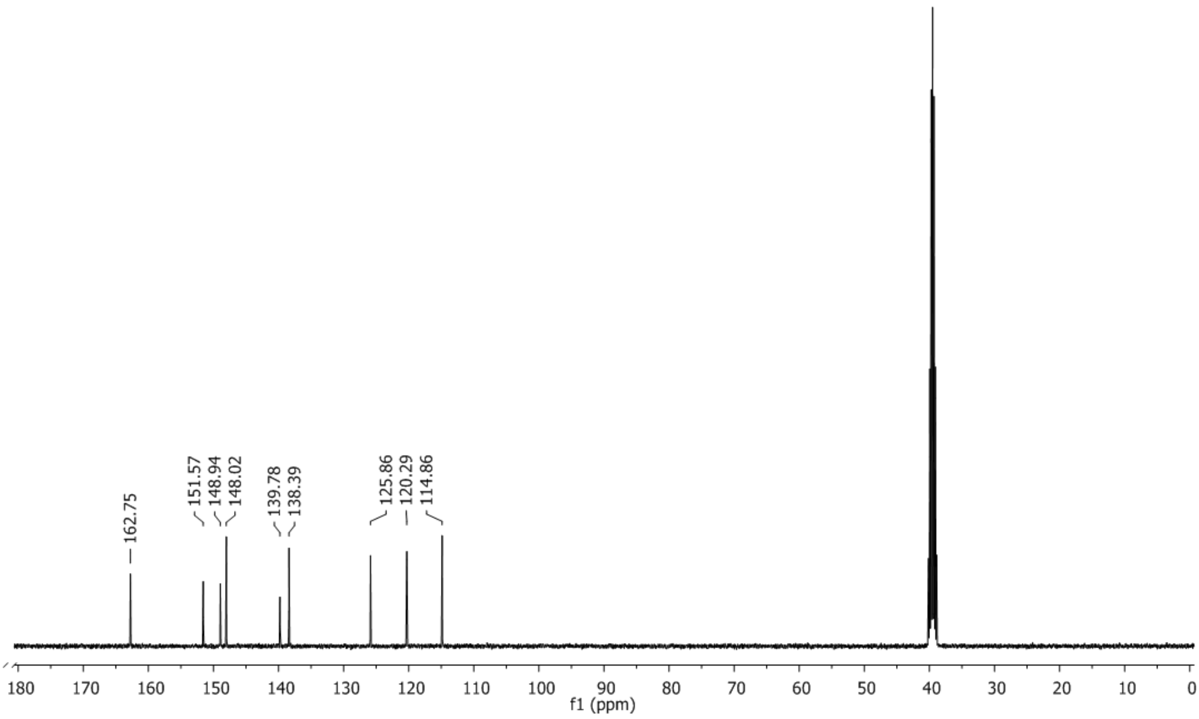

Figure S36. ^{13}C NMR spectrum of *N*[2],*N*[6]-di(pyridin-2-yl)pyridine-2,6-dicarboxamide (**18**).

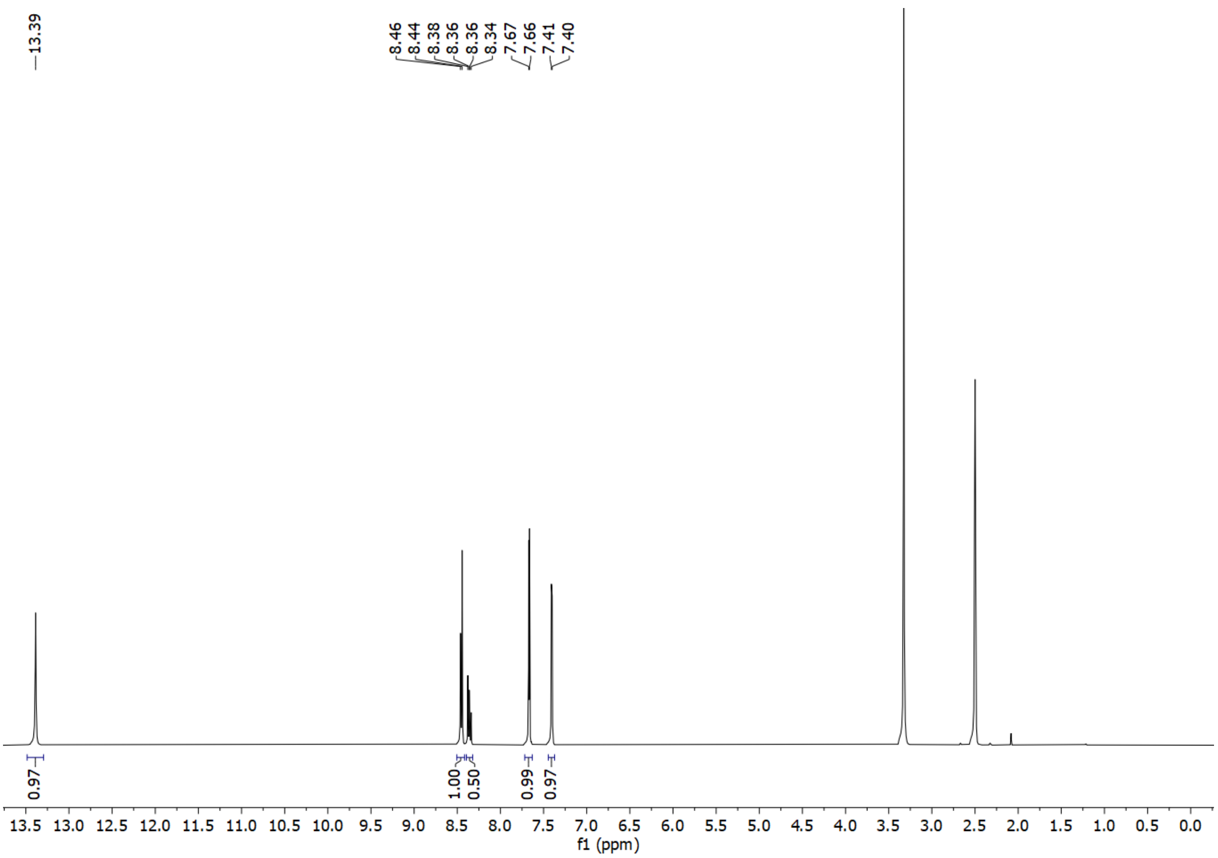

Figure S37. ^{1}H NMR spectrum of N^2,N^6-di(1,3-thiazol-2-yl)pyridine-2,6-dicarboxamide (**19**).

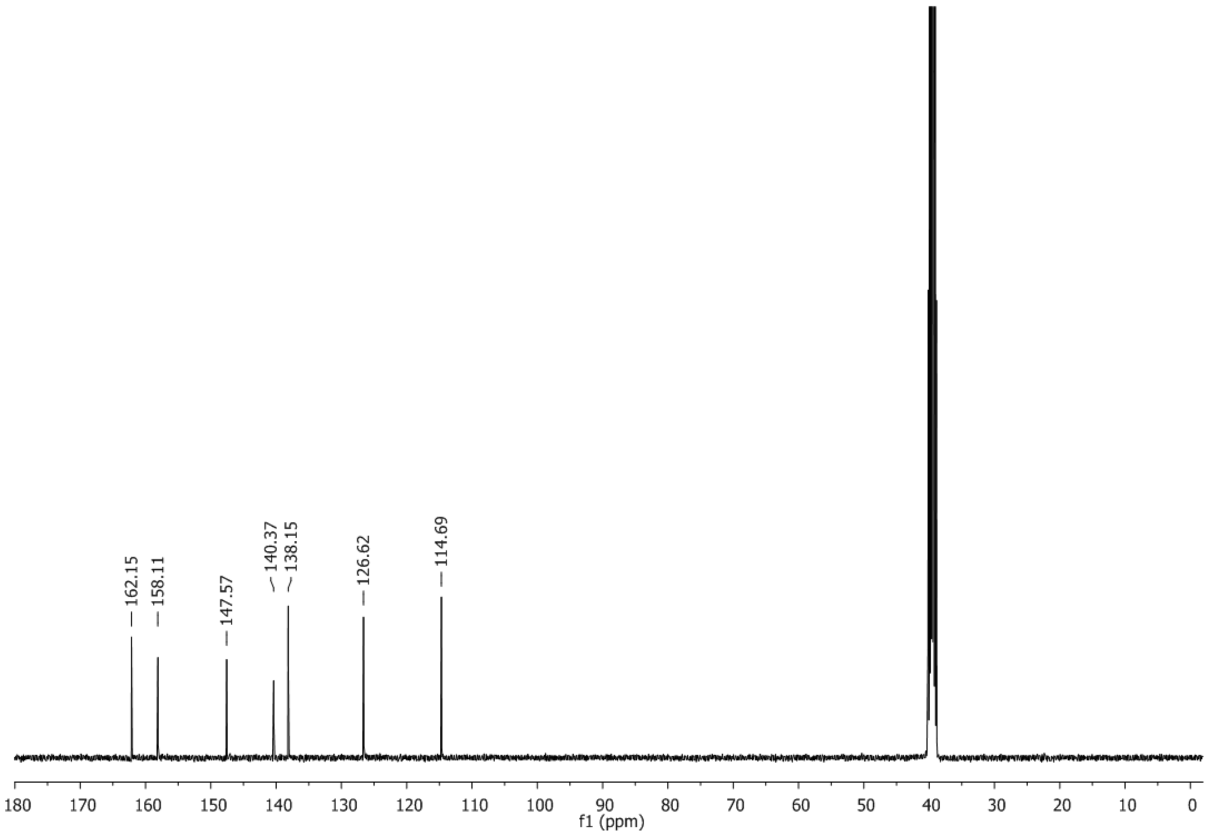

Figure S38. ^{13}C NMR spectrum of N^2,N^6-di(1,3-thiazol-2-yl)pyridine-2,6-dicarboxamide (**19**).

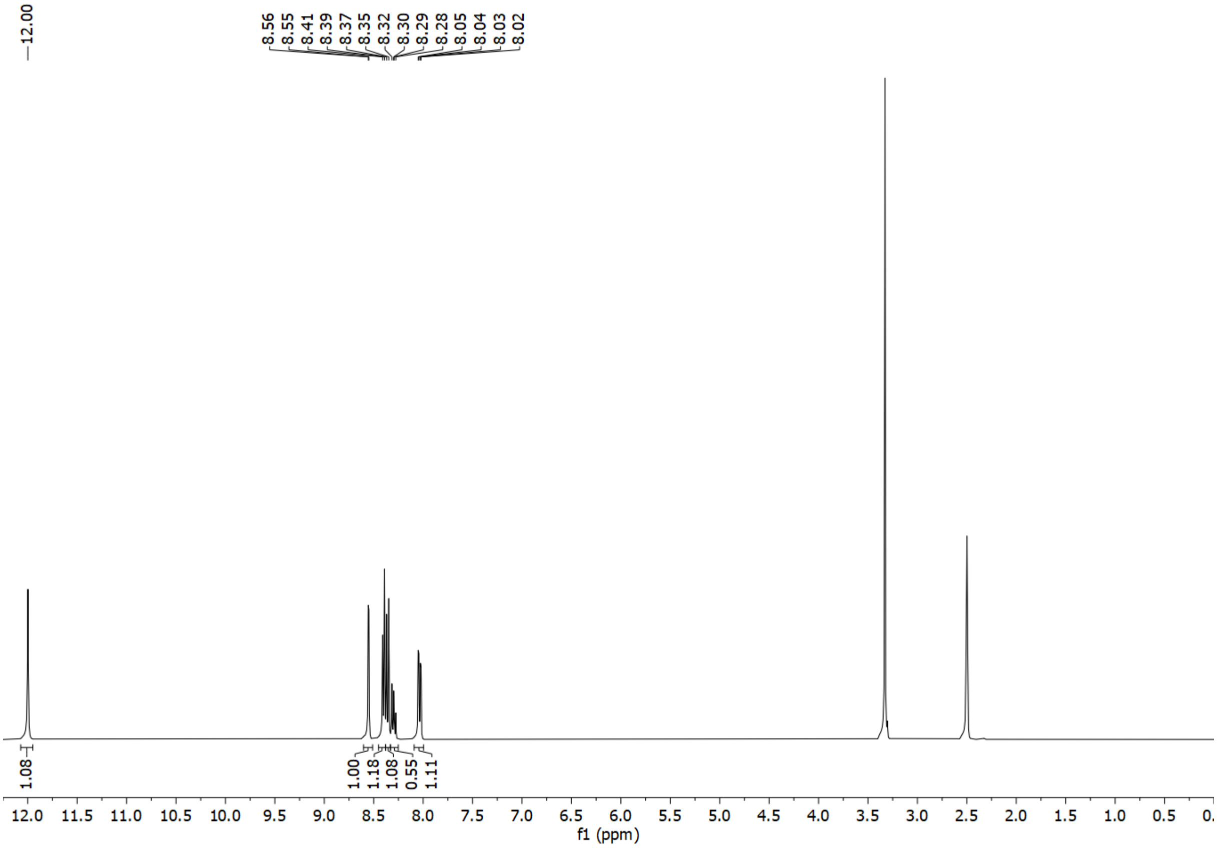

Figure S39. ^{1}H NMR spectrum of N^2,N^6-bis(5-chloropyridin-2-yl)pyridine-2,6-dicarboxamide (**20**).

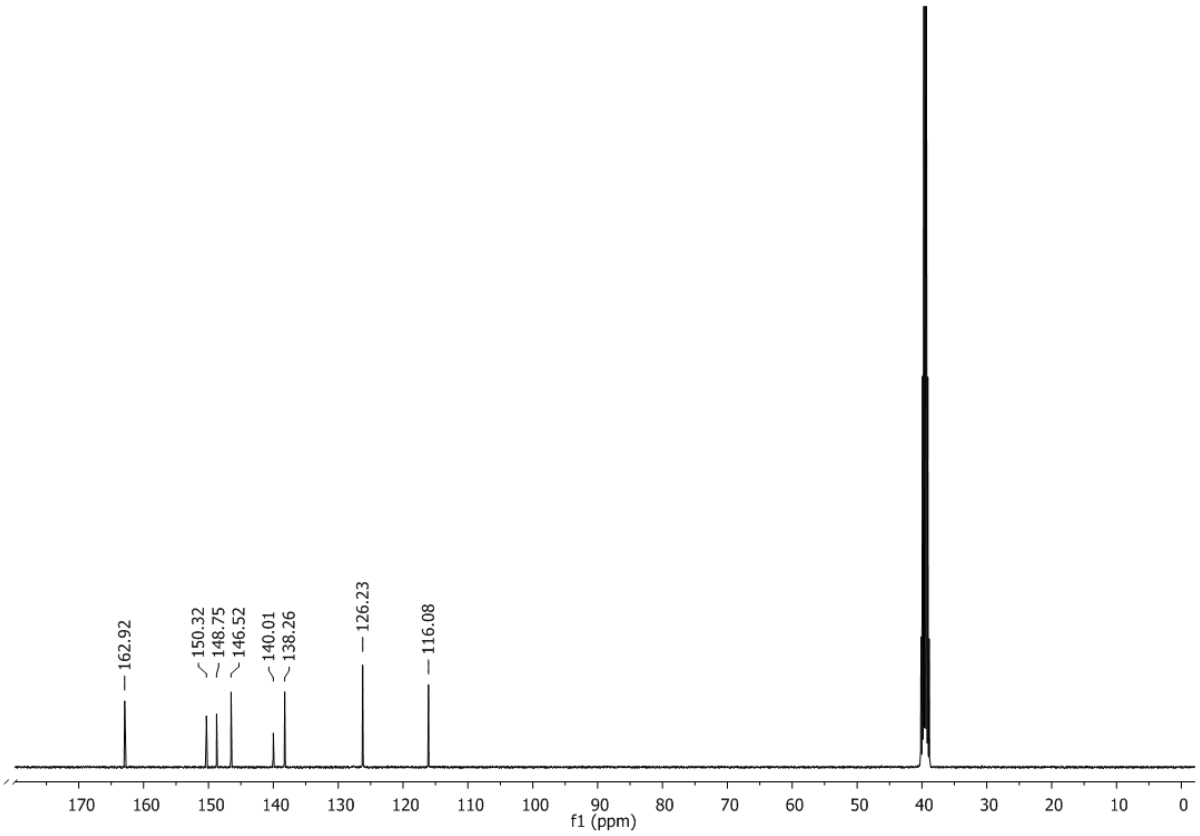

Figure S40. ^{13}C NMR spectrum of N^2,N^6-bis(5-chloropyridin-2-yl)pyridine-2,6-dicarboxamide (**20**).

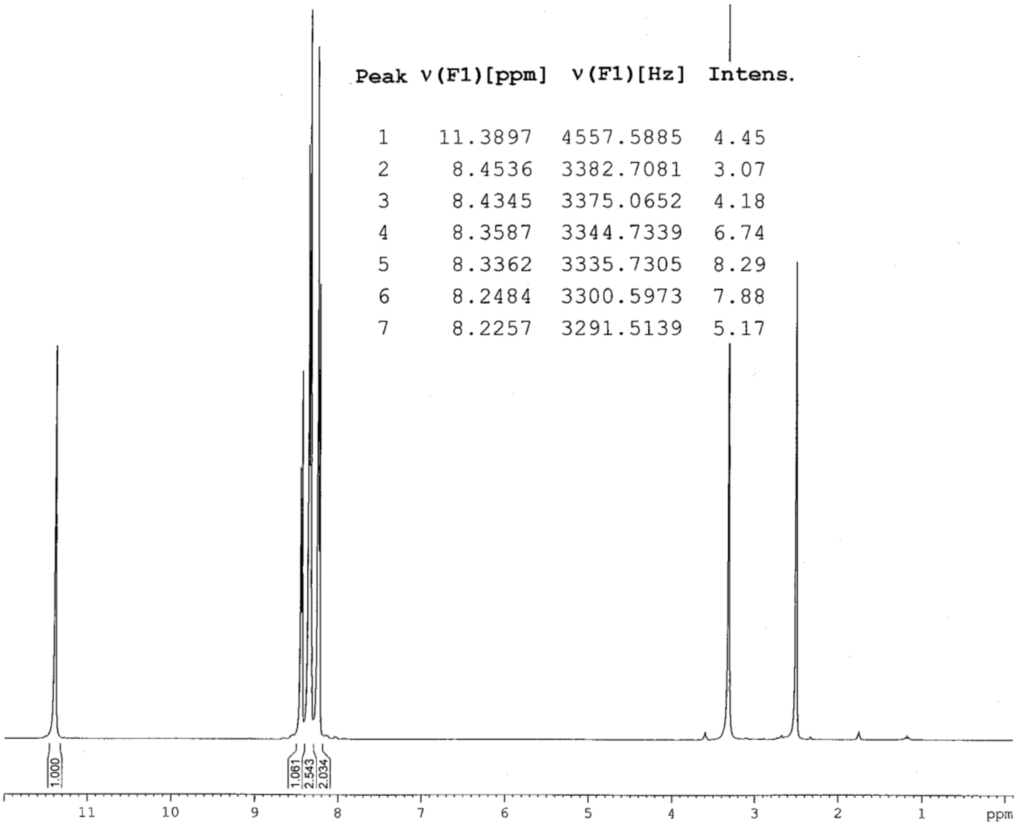

Figure S41. ^{1}H NMR spectrum of *N*^{2},*N*^{6}-di(4-nitrophenyl)pyridine-2,6-dicarboxamide (**21**).

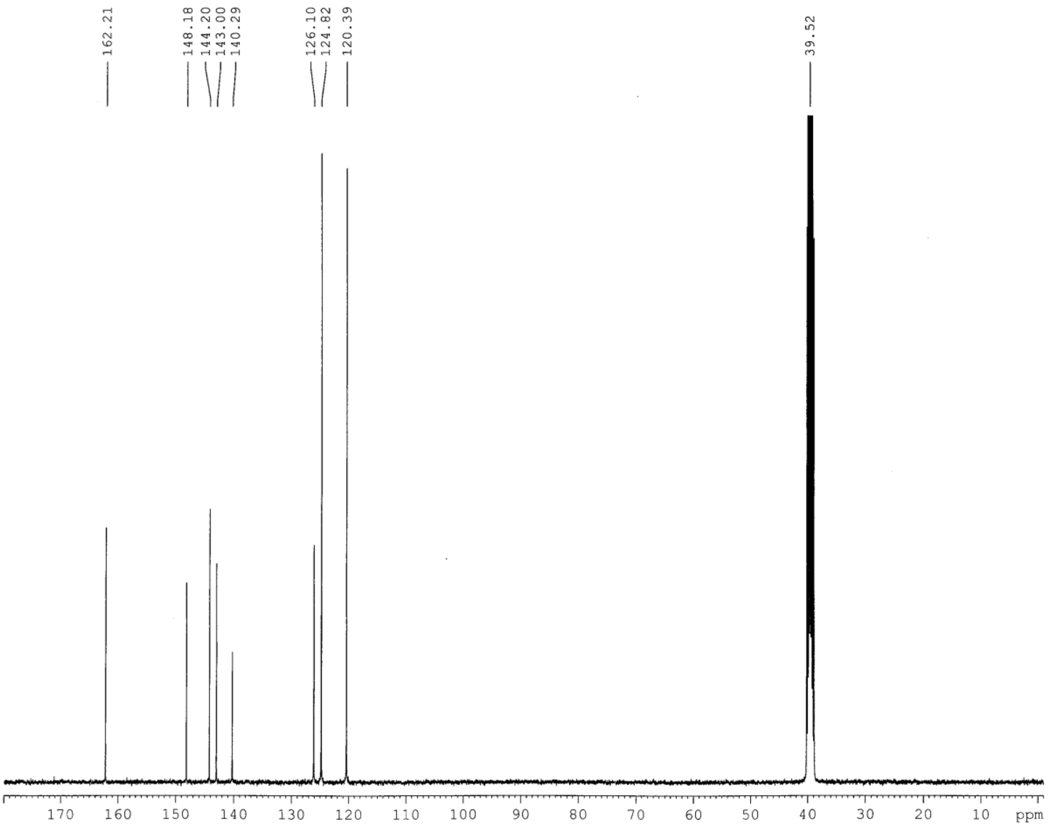

Figure S42. ^{13}C NMR spectrum of *N*^{2},*N*^{6}-di(4-nitrophenyl)pyridine-2,6-dicarboxamide (**21**).

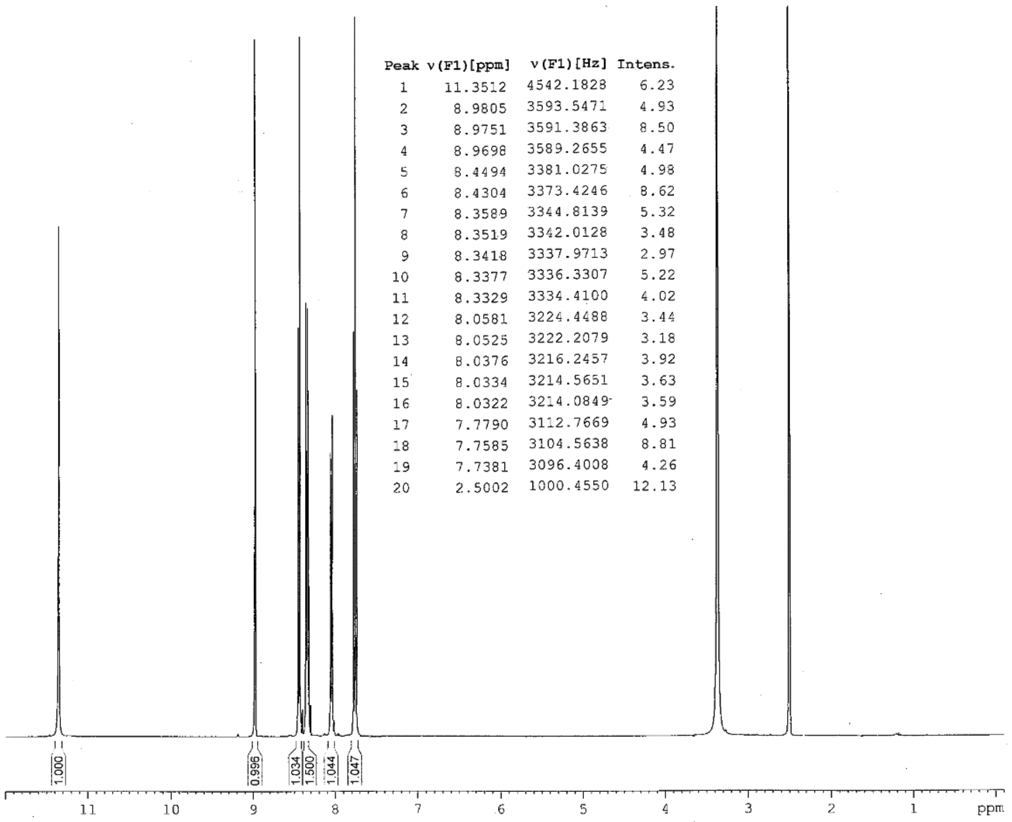

Figure S43. ^{1}H NMR spectrum of *N*[2],*N*[6]-di(3-nitrophenyl)pyridine-2,6-dicarboxamide (**22**).

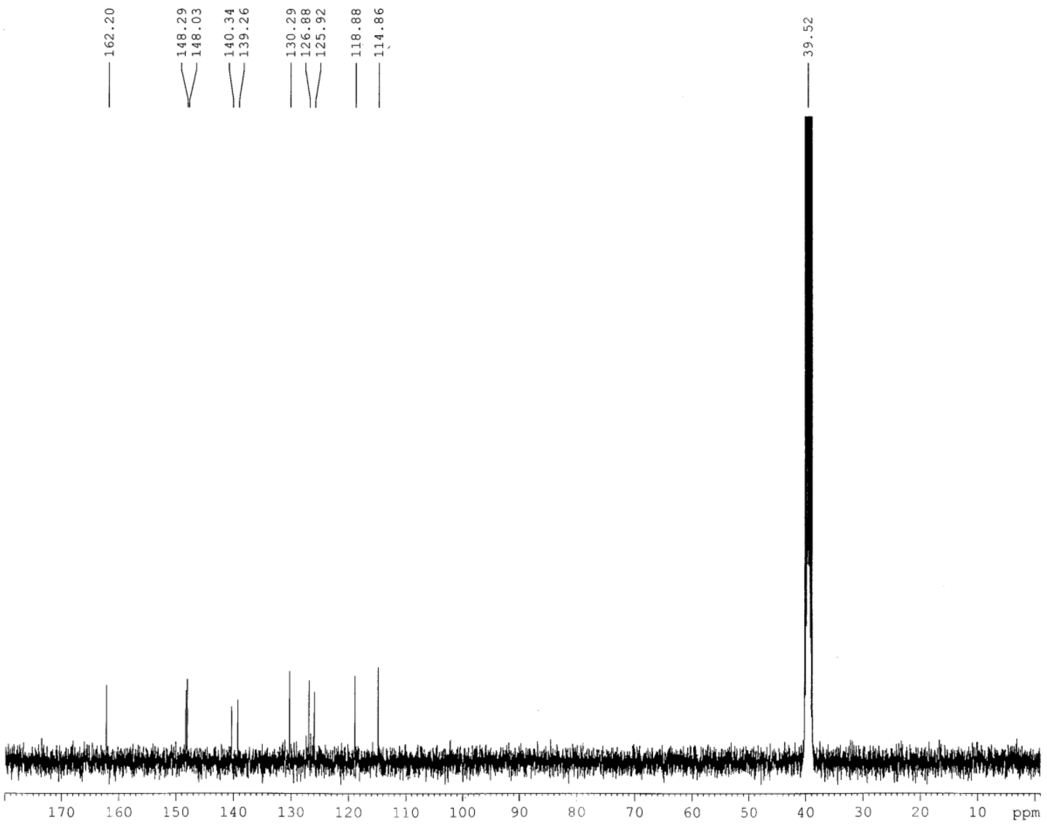

Figure S44. ^{13}C NMR spectrum of *N*[2],*N*[6]-di(3-nitrophenyl)pyridine-2,6-dicarboxamide (**22**).

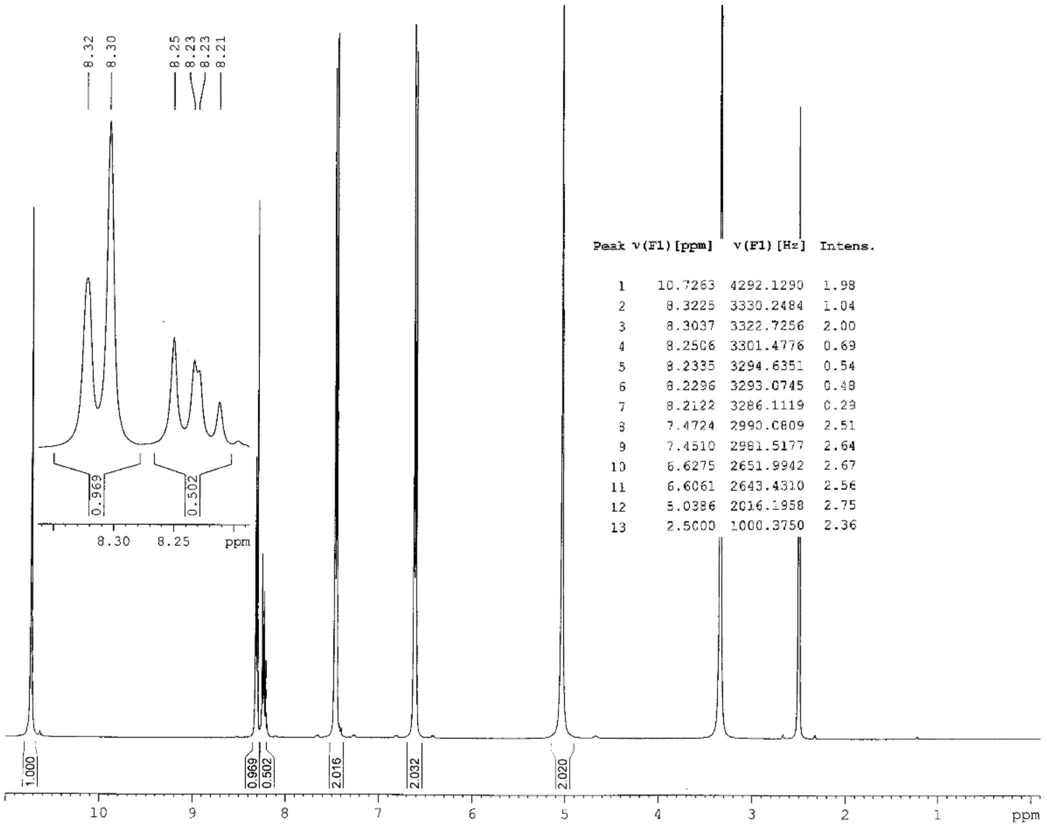

Figure S45. ^{1}H NMR spectrum of *N*[2],*N*[6]-di(4-aminophenyl)pyridine-2,6-dicarboxamide (**23**).

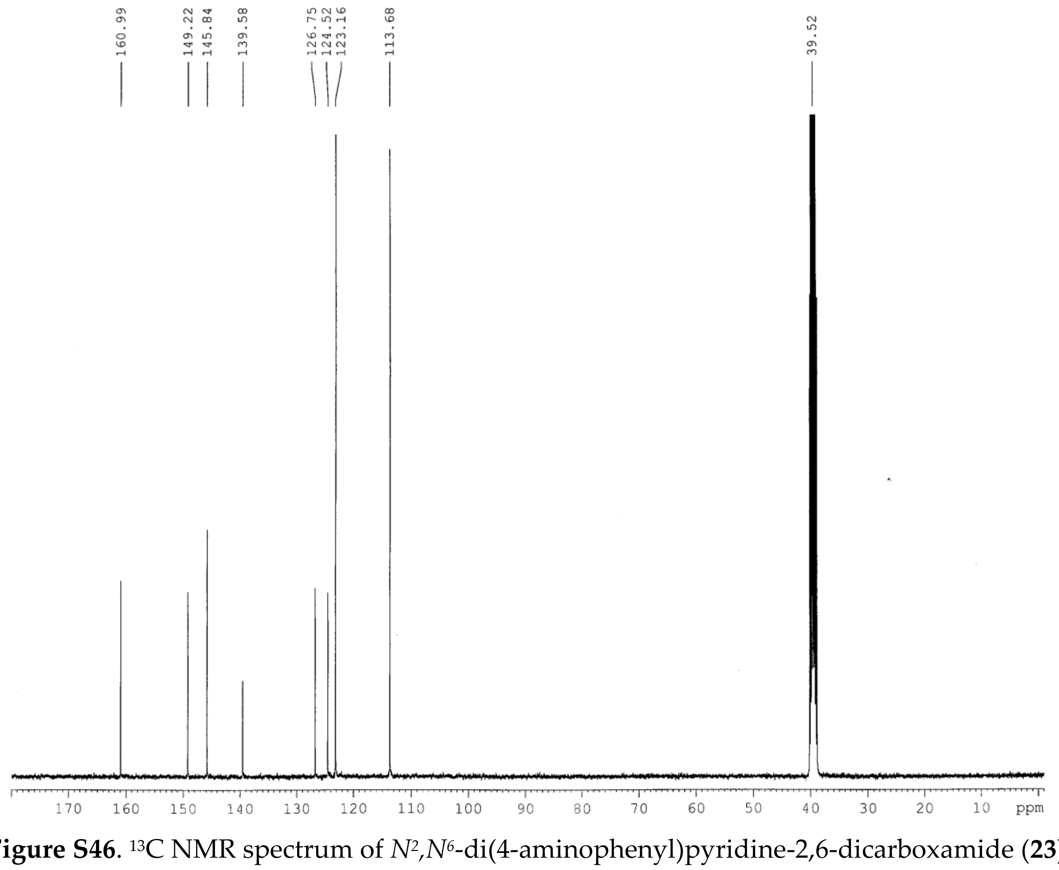

Figure S46. ^{13}C NMR spectrum of *N*[2],*N*[6]-di(4-aminophenyl)pyridine-2,6-dicarboxamide (**23**).

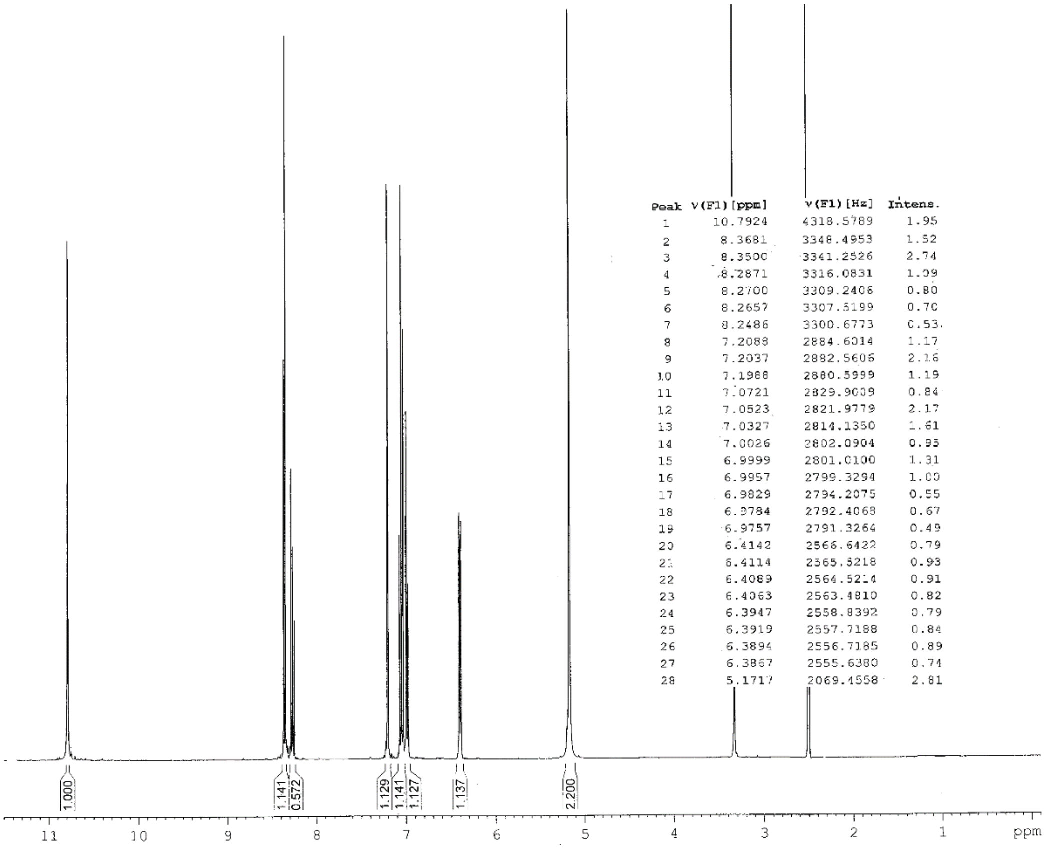

Figure S47. ^{1}H NMR spectrum of N^2,N^6-di(3-aminophenyl)pyridine-2,6-dicarboxamide (24).

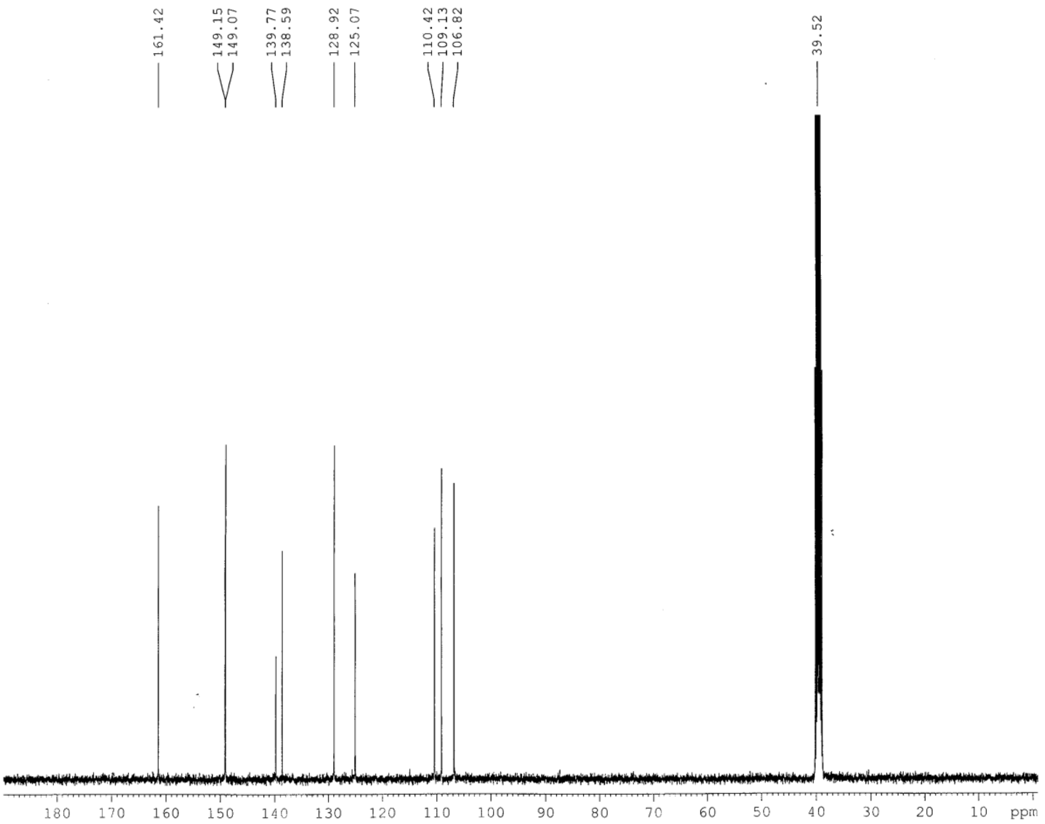

Figure S48. ^{13}C NMR spectrum of N^2,N^6-di(3-aminophenyl)pyridine-2,6-dicarboxamide (**24**).

Table S1. Crystal data and structure refinement details for compounds: **3**, **4**, **5**, **7**, **10**, **16** and **23**.

**Dataset**	**3**	**4**	**5**	**7**	**10**	**16**	**23**
Unit cell content	$C_{14}H_{10}N_6O_3{\cdot}H_2O$	$C_{16}H_{12}N_4O_3$	$C_{16}H_{12}N_4O_3{\cdot}H_2O$	$(C_{12}H_8N_4O_3S_2)_2$	$C_{18}H_{12}N_4O_7{\cdot}C_3H_6O{\cdot}H_2O$	$C_{17}H_{13}N_5O_2{\cdot}3H_2O$	$C_{19}H_{17}N_5O_2$
Molecular weight	328.30	308.30	326.31	640.68	472.41	373.37	347.38
Temperature [K]	100(2)	100(2)	100(2)	100(2)	100(2)	100(2)	100(2)
Wavelength [Å]	1.54184	1.54184	1.54184	1.54184	0.71073	0.71073	1.54184
Crystal system	monoclinic	monoclinic	monoclinic	monoclinic	monoclinic	monoclinic	monoclinic
Space group	$P2_1/n$	$P2_1/c$	$P2_1/n$	$P2_1/c$	$P2_1/c$	$P2_1/n$	$P2_1/n$
Unit cell parameters [Å, °]							
a	8.8800 (1)	13.0596 (2)	9.0320 (1)	7.094 (1)	6.8292 (3)	9.0360 (2)	8.2787 (1)
b	8.0526 (1)	8.5996 (1)	8.3075 (1)	10.648 (2)	24.0781 (11)	21.7801 (4)	14.0987 (2)
c	20.0986 (1)	13.6980 (3)	20.0242 (2)	34.551 (7)	13.2063 (7)	9.7786 (3)	17.2782 (2)
β	101.633 (1)	115.818 (2)	102.393 (1)	95.89 (3)	102.572 (5)	112.455 (3)	97.615 (1)
Volume [$Å^3$]	1407.68 (2)	1384.83 (5)	1467.46 (3)	2596.1 (9)	2119.50 (18)	1778.56 (8)	1998.91 (4)
Z	4	4	4	8	4	4	4
Absorption coefficient [mm^{-1}]	1.00	0.88	0.92	3.99	0.12	0.11	0.64
F(000)	680	640	680	1312	984	784	728
Crystal size [mm^3]	0.24×0.04×0.03	0.30×0.28×0.04	0.26×0.22×0.19	0.36×0.08×0.08	0.38×0.11×0.08	0.40×0.34×0.03	0.25×0.05×0.03
θ range for data collection [°]	4.5 to 76.5	3.8 to 76.5	4.5 to 77.2	4.4 to 68.2	3.0 to 28.7	2.4 to 33.2	4.1 to 76.8
Index ranges	−11←*h*←11<br>−10←*k*←10<br>−25←*l*←25	−16←*h*←16<br>−10←*k*←10<br>−15←*l*←17	−11←*h*←11<br>−10←*k*←10<br>−25←*l*←25	−6←*h*←8<br>−12←*k*←12<br>−41←*l*←40	−9←*h*←9<br>−32←*k*←32<br>−4←*l*←17	−13←*h*←6<br>−33←*k*←32<br>−14←*l*←14	−9←*h*←10<br>−17←*k*←17<br>−21←*l*←21
Reflections collected	55735	17723	38700	15940	16124	18398	25625
Independent reflections, R_{int}	2941, 0.053	2882, 0.030	3101, 0.031	4627, 0.021	5471, 0.030*	6226, 0.027	4196, 0.035
Data / restraints / parameters	2941 / 0 / 230	2882 / 0 / 215	3101 / 0 / 229	4627 / 0 / 391	5471 / 0 /322*	6226 / 0/ 268	4196 / 0 /242
Goodness-of-fit on *F*2	1.04	1.07	1.03	1.03	1.11*	1.06	1.06
Final *R* indices (all data)	*R*1 = 0.035<br>*wR*2 = 0.094	*R*1 = 0.033<br>*wR*2 = 0.087	*R*1 = 0.036<br>*wR*2 = 0.099	*R*1 = 0.027<br>*wR*2 = 0.072	*R*1 = 0.072*<br>*wR*2 = 0.195*	*R*1 = 0.044<br>*wR*2 = 0.128	*R*1 = 0.061<br>*wR*2 = 0.184
Largest diff. peak and hole [e·$Å^{-3}$]	0.28 and -0.21	0.27 and -0.21	0.19 and -0.27	0.30 and -0.23	0.43 and -0.43*	0.55 and -0.30	0.76 and -0.37
CCDC number	2065258	2065253	2065257	2065255	2065252	2065256	2065254

* values correspond to an analysis and refinement based on crystallographic data in HKLF 5 format generated by *TwinRotMat* program.
